# Efficacy and safety of Chinese herbal medicine for multiple sclerosis: a systematic review and meta-analysis of randomized controlled trials

**DOI:** 10.3389/fphar.2025.1635833

**Published:** 2025-09-22

**Authors:** Xiaorui Guan, Yufan Wu, Qiuyang Jia, Yilan Zheng, Mingrun Zou, Jia Liu, Kazuo Sugimoto, Ying Gao

**Affiliations:** ^1^ Department of Neurology, Dongzhimen Hospital, Beijing University of Chinese Medicine, Beijing, China; ^2^ Institute for Brain Disorders, Beijing University of Chinese Medicine, Beijing, China

**Keywords:** multiple sclerosis, Chinese herbal medicine, complementary and alternative medicine, randomized controlled trials, meta-analysis

## Abstract

**Background:**

Multiple sclerosis (MS) is an incurable, chronic, disabling disease that primarily affects young adults. Chinese herbal medicine (CHM) is increasingly recognized as a major form of complementary and alternative medicine; however, there is a limited number of systematic analyses regarding its therapeutic effects on MS.

**Aim of the study:**

This study aimed to evaluate whether CHM, when used alongside conventional treatment, offers additional therapeutic benefits for patients with MS, focusing on clinical efficacy and safety.

**Materials and methods:**

We searched eight databases from their inception until July 2025 to identify eligible randomized controlled trials (RCTs) involving CHM therapy for MS. Key outcomes assessed included the expanded disability status scale (EDSS), annual relapse rate (ARR), neurological signs scores, clinical symptoms scores, and modified fatigue impact scale (MFIS). The risk of bias was evaluated using the Cochrane Handbook for systematic reviews, while quantitative synthesis was performed with RevMan (version 5.4.1) and Stata (version 18.0) software. This review has been registered at the International Prospective Register of Systematic Reviews database (Registration No. CRD42024605890).

**Results:**

This systematic review and meta-analysis included 28 RCTs involving a total of 1,971 participants. The combination of CHM and conventional therapy was superior to conventional therapy alone for individuals with MS. This was evidenced by a decrease in the EDSS (mean difference (MD) = −0.65; 95% confidence interval (CI): −0.96, −0.35; *P* < 0.0001; MD = −0.65, ARR (relative risk (RR) = 0.48; 95% CI: 0.31, 0.73; *P* = 0.0007), neurological signs scores (MD = −2.96; 95% CI: −4.52, −1.39; *P* = 0.0002), and MFIS (MD = −8.55; 95% CI: −10.20, −6.89; *P* < 0.00001). Additionally, there was an improvement in clinical effects (RR = 1.25; 95% CI: 1.19, 1.32; *P* < 0.00001). No serious adverse effect was reported.

**Conclusion:**

Although the methodological quality of the included RCTs was relatively suboptimal, CHM therapy combined with conventional therapy manifests a promising effectiveness and safety for managing MS. Therefore, well-designed clinical studies are necessary to provide high-quality evidence.

**Systematic review registration:**

https://www.crd.york.ac.uk/PROSPERO/view/CRD42024605890, identifer CRD42024605890.

## 1 Introduction

Multiple sclerosis (MS) is an autoimmune disease characterized by demyelinating lesions in the white matter of the central nervous system. It typically manifests between the ages of 18 and 40 (Jakimovski et al., 2024). MS is one of the most common causes of neurological disability among young adults, affecting more females than males (Tian et al., 2020). Although the exact cause of MS remains unclear, factors such as genetic susceptibility, environmental influences, infections, and vitamin D deficiency could increase susceptibility (Waubant et al., 2019). Clinical manifestations include paralysis, numbness, painful spasms, aphasia, visual impairment, ataxia, psychiatric symptoms, or intellectual disabilities (McGinley et al., 2021). According to the type of disease course, MS is divided into relapsing remitting MS, secondary progressive MS, primary progressive MS, and other types (Kolčava et al., 2020).

Despite ongoing advancements in treatment methods, MS remains an incurable, chronic, disabling disease. The primary objectives of treatment are to reduce the frequency of recurrence, reduce life burden, and improve quality of life. During acute exacerbations, hormone therapies are the first-line treatment. In the remission phase, disease-modifying therapies and symptomatic treatments are recommended. However, these treatments may present drawbacks, including a risk of opportunistic infections, secondary autoimmune reactions, and hormone-related side effects (Rae-Grant et al., 2018).

Patients suffering from MS tend to seek help from complementary and alternative medicine (CAM), which is used in 33%–80% of patients with MS in developed countries (Yadav et al., 2014; Liu et al., 2022). In Huangdi Neijing (2005), numerous classical symptom patterns consistent with those observed in MS are documented, such as “wei zheng” (flaccidity syndrome) and “shizhan hunmiao” (blurred vision due to deficiency). Treatment principles in traditional Chinese medicine (TCM) are based on syndrome differentiation, with common patterns including kidney yin deficiency, internal accumulation of turbid toxins, and phlegm-blood stasis. Herbal formulas are selected accordingly to tonify deficiency, eliminate turbid toxins, promote blood circulation, or clear heat and dampness. Modern pharmacological studies suggest that Chinese herbal medicine (CHM), a major form of CAM, may exert immunomodulatory and anti-inflammatory effects, providing a potentially valuable adjunct to conventional therapies in MS management. Baicalin is a bioactive flavonoid compound derived from the root of *Scutellaria baicalensis* Georgi. Zhang et al. (2015) found that intraperitoneal administration of baicalin (100 mg/kg) for 15 consecutive days significantly improved disease severity in Experimental Autoimmune Encephalomyelitis (EAE) mice induced by Myelin Oligodendrocyte Glycoprotein 35–55, an established animal model of MS. Similarly, matrine is a quinolizidine alkaloid component derived from the root of *Sophora flavescens* Aiton. Chu et al. (2021) administered matrine at 100 mg/kg intraperitoneally for 11 consecutive days in EAE mice, resulting in significantly reduced disease severity, decreased inflammatory infiltration, and attenuated demyelination. Two meta-analyses (Song et al., 2017; Liu et al., 2012) published 8 years ago indicated that CHM therapy positively affects patients with MS. Given the number of randomized controlled trials (RCTs) conducted in recent years, this study aimed to update the evidence and re-evaluate the impact of CHM therapy on MS.

## 2 Methods

### 2.1 Protocol registration

The systematic review and meta-analysis have been registered at the International Prospective Register of Systematic Reviews database (registration No. CRD42024605890). This study was conducted following the Preferred Reporting Items for Systematic reviews and Meta-Analyses (PRISMA) statement (Page et al., 2021) and PRISMA-CHM checklist (Zhang et al., 2020).

### 2.2 Eligibility criteria

1. Types of studies: RCTs were included regardless of language and publication status.
2. Types of participants: Patients diagnosed with MS were included without restrictions of gender, age, nationality, and geographical residence. There were also no limitations regarding disease course, duration, neurological disability, or comorbidities.
3. Types of interventions: The studies that used CHM as the intervention in the experimental group were included. According to The World Health Organization (2021), herbal medicines (HMs) encompass herbs, herbal materials, herbal preparations, and finished herbal products containing active ingredients derived from plants or other plant sources. CHM is a specific subset of HMs that is formulated and prescribed based on TCM, including syndrome differentiation and individualized or standardized herbal formulas. CHM interventions could be administered in various forms such as decoctions, granules, tablets, capsules, or other oral formulations. There were no restrictions on the frequency or dosage of CHM. The treatment course needed to last a minimum of 3 weeks.
4. Types of comparators: The studies that used conventional treatment (CT) with or without placebo as the intervention of the control group were included. If the experimental group received a combined treatment of CHM and CT, the CT should be administered equally across all trial groups.
5. Types of outcome measures: In this study, the Expanded Disability Status Scale (EDSS) was selected as the primary outcome. EDSS is the most widely used and internationally recognized clinical measure for evaluating neurological disability in patients with MS. Developed by Kurtzke (Kurtzke, 1983), the scale comprehensively evaluates eight functional systems, with a strong emphasis on ambulation. Its broad application in clinical trials and sensitivity to disease progression make it a robust indicator for treatment efficacy. Secondary outcomes of interest included annual relapse frequency and annual relapse rate (ARR), neurological signs scores, clinical symptoms scores, modified fatigue impact scale (MFIS), magnetic resonance imaging (new T2 lesions or gadolinium-enhanced lesions, change in volume of T2 lesions), scores of TCM syndrome, cognitive function, the MS functional composite (MSFC), pharmacodynamic biomarkers, clinical effect, and adverse effect.

### 2.3 Search strategy

Two independent reviewers (GXR and WYF) searched various electronic databases from inception to July 2025. The databases included PubMed, Cochrane Library, Web of Science, EMBASE, the Chinese National Knowledge Infrastructure, Wanfang Database, the Chinese Biomedical Database (SinoMed), and the Chinese Science and Technology Journals Database (VIP). No restrictions were imposed on the language of publication. The search terms used included MS and its synonyms, in combination with CHM or their proprietary names, utilizing MeSH and as free-text words. Chinese databases were also searched using the above search terms in Chinese. The detailed searching strategies are listed in the Supplementary Text (Supplementary Appendix 1).

### 2.4 Study selection and data extraction

According to prespecified selection criteria, two authors (GXR and WYF) independently reviewed the titles and abstracts of articles after deduplication. The articles that did not fulfil the inclusion criteria were removed. The remaining articles were screened with full text by the same two authors independently. Any disagreements in primary screening were to be discussed and resolved. A third review author (LJ) was consulted if required. For articles that met the inclusion criteria, two authors (GXR and JQY) extracted study characteristics and relevant outcome data. This included the first author’s name, publication year, sample size, age, gender, interventions and comparisons, and the course of treatment. The primary outcome was the EDSS, while secondary outcomes included annual relapse frequency (ARR), neurological signs scores, clinical symptoms scores, MFIS, MRI, scores of TCM syndrome, cognitive function, MSFC, and pharmacodynamic biomarkers. Adverse effects were also recorded. If patient data were reported in multiple articles, only the one with the most complete information was included.

### 2.5 Quality assessment

We conducted a methodological quality assessment of randomized trials using the Cochrane Handbook for Systematic Reviews of Interventions (Sterne et al., 2019). Two authors (GXR and WYF) independently assessed the risk of bias across five domains: (1) bias arising from the randomization process; (2) bias due to deviations from intended interventions; (3) bias due to missing outcome data; (4) bias in outcome measurement; (5) bias in the selection of reported results. Each domain was classified as having a “High risk,” “Low risk,” or “Some concerns”. According to the ROB 2.0 guidelines, a study was judged to have an overall low risk of bias (RoB) if it was rated as low risk across all domains for a given outcome. Studies with a high risk rating in any domain, or with “some concerns” in three or more domains, were considered to have a high overall RoB. Studies that had “some concerns” in at least one domain, but were not rated as high risk in any domain, were judged to have an overall RoB of “some concerns”. Any disagreements between the reviewers were resolved through discussion or arbitration by a third investigator (JQY).

### 2.6 Data synthesis

We analyzed the data using the RevMan (version 5.4.1) software. Relative risk (RR) was used as the effect size for binary variables, while the mean difference (MD) or standard mean difference (SMD) was employed for continuous variables. We used 95% confidence intervals (CIs) to represent the effect size interval for both variables. The Cochrane Q test and I^2^ values were used to assess the heterogeneity between trials. If *P* < 0.05 or I^2^ > 50%, the random effect model was selected to calculate the pooled effect size; otherwise, we used the fixed effects model. To visually present the results of the syntheses, we constructed forest plots. Subgroup analysis was conducted to identify possible causes of heterogeneity. A sensitivity analysis was performed by iteratively omitting one study at a time to determine the stability of the pooled effects. Finally, publication bias was quantitatively assessed using funnel plots and Egger’s tests, employing RevMan (version 5.4.1) and Stata (version 18.0) software.

### 2.7 Quality of evidence

The Grading of Recommendations, Assessment, Development and Evaluation (GRADE) system was utilized by two independent reviewers to assess the quality of evidence for each outcome indicator. There are five evaluation aspects, including limitations, inconsistency, indirectness, imprecision, and other factors. The evidence was rated across four levels: Very low, low, medium, and high were rated for the above five domains.

## 3 Results

### 3.1 Study selection

**FIGURE 1:**
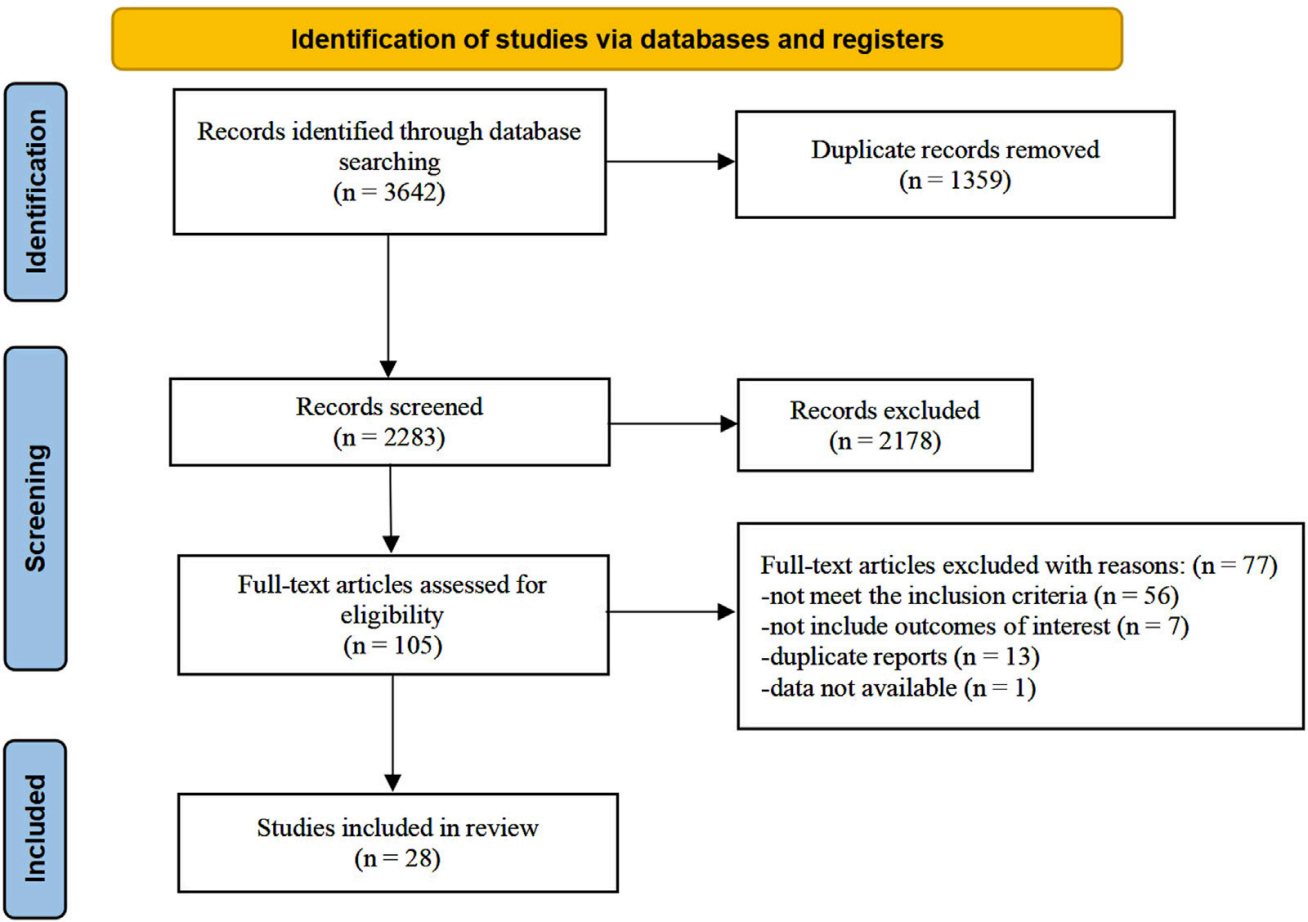
Flowchart of the study selection process.

The research selection process is illustrated in Figure 1. According to our strategy, 3,642 records were retrieved. Among these, 1,359 records were removed due to duplication. After screening titles and abstracts, we excluded 2,178 papers for various reasons, including that they were not clinical trials, did not involve Chinese medicine interventions, or did not include participants with MS. We further evaluated 105 full-text articles for eligibility. The following factors led to the exclusion of 77 studies based on the selection criteria: 56 studies did not meet the inclusion criteria; seven studies did not address the relevant outcomes; 13 studies were duplicate reports, and the data of one study were unavailable (Bertoglio et al., 2016). Finally, 28 trials were included in the final analysis.

### 3.2 Study characteristics

The characteristics of the 28 included RCTs are listed in Table 1. All 28 studies included were randomized clinical trials published between 2004 and 2025. A total of 27 studies were conducted in China, and one was conducted in Iran. Overall, 1971 patients were enrolled in this review, with a sample size ranging from 26 to 271. The intervention group comprised 1,013 patients, while the control group included 958 patients. Among the participants, 65.0% were female, and their ages ranged from 13 to 78 years. The medications used in the intervention group included herbal preparations and commercial Chinese polyherbal preparations. The composition of each herbal formula in each trial is listed in Table 2. The treatment duration varied from 3 weeks to 2 years, with most trials having a follow-up period of 3 months. In terms of outcome indicators, 23 RCTs reported EDSS, 3 RCTs provided ARR, 7 RCTs presented clinical symptom scores, and 5 RCTs reported neurological signs scores. Additionally, 18 RCTs evaluated clinical effects, 2 RCTs provided the MFIS, 3 RCTs presented MRI-related outcomes, 3 RCTs reported cognitive function outcomes, 4 RCTs presented scores of TCM syndrome, and 1 RCT reported MSFC. Furthermore, 6 RCTs presented pharmacodynamic biomarkers and 12 RCTs reported adverse effects.

**TABLE 1:** Characteristic of included RCTs.

First author, public year	Sample size (T/C)	Diagnostic criteria	Type of patients	Age (year)	Course of disease (years)
Ayoobi et al. (2019)	65 (43/22)	McDonald criteria 2010	RRMS (remission phase)	T: 250 mg 31.36 ± 9.859; 500 mg 34.00 ± 8.515 C: 34.68 ± 7.828	—
Bi (2016)	52 (26/26)	McDonald criteria 2010	RRMS	T: 33.6 ± 8.3 C: 32.6 ± 8.0	—
Chen and Fan (2016)	60 (30/30)	McDonald criteria 2005	MS (remission phase)	T: 39.20 ± 10.80 C: 37.46 ± 11.09	T: 3.64 ± 4.17 C: 4.43 ± 3.20
Chen et al. (2006)	90 (60/30)	Diagnosis and differential diagnosis of multiple sclerosis; 1983 poser revised diagnostic criteria for MS	RRMS + SPMS + PPMS + PRMS + benign MS + malignant MS	T: 32.1 ± 23.9 C: 31.6 ± 23.1	T: 61.5 ± 62.1 (months) C: 60.5 ± 61.6 (months)
Dong (2014)	271 (136/135)	—	RRMS + SPMS + PPMS + PRMS	—	T: 6 months–2 years C: 5 months–2 years
Duan (2013)	42 (20/22)	5th edition of Neurology	RRMS + SPMS + PPMS + PRMS	T: 38 ± 3 C: 39 ± 5	—
Fan et al. (2006)	65 (30/35)	1983 Poser revised diagnostic criteria for MS	MS (acute exacerbation phase)	T: 38.10 ± 12.48 C: 36.46 ± 14.13	—
Fan et al. (2018)	50 (24/26)	McDonald criteria 2005	RRMS (remission phase)	T: 31.63 ± 10.05 C: 32.73 ± 9.84	T: 65.63 ± 69.85 (months) C: 43.85 ± 51.37 (months)
Gao et al. (2008)	78 (38/40)	1983 Poser revised diagnostic criteria for MS	MS (acute exacerbation phase)	T: 37.10 ± 7.56 C: 36.35 ± 7.67	—
Jie (2024)	62 (31/31)	Chinese consensus on diagnosis and treatment on multiple sclerosis (2014 Version)	MS (acute exacerbation phase)	T: 42.51 ± 3.05 C: 42.71 ± 3.08	—
Li (2012)	49 (28/21)	1983 Poser revised diagnostic criteria for MS	MS	T: 32.1 ± 10.2 C: 33.5 ± 9.7	T: 2.4 ± 1.5 C: 2.2 ± 1.6
Li (2017)	90 (45/45)	—	MS	T: 34.24 ± 5.21 C: 34.21 ± 5.24	T: 1.24 ± 0.21 C: 1.21 ± 0.25
Li and Yan (2017)	26 (14/12)	McDonald criteria 2010	RRMS	T: 62.7 ± 8.6 C: 64.2 ± 5.6	—
Li and Zhao (2012)	60 (30/30)	McDonald criteria 2005	MS	T: 40.24 ± 2.53 C: 42.31 ± 1.24	T: 3.3 ± 1.25 C: 3.26 ± 1.08
Li et al. (2019)	82 (41/41)	McDonald criteria 2010	MS (acute exacerbation phase)	T: 32.5 ± 4.3 C: 32.8 ± 4.2	T: 2.2 ± 0.6 C: 2.4 ± 0.7
Li et al. (2024)	72 (36/36)	Diagnosis and Differential Diagnosis of Multiple Sclerosis	RRMS (remission phase)	T: 39.23 ± 6.95 C: 37.14 ± 5.39	T: 6.53 ± 1.49 C: 5.98 ± 1.14
Li et al. (2025)	86 (43/43)	Chinese consensus on diagnosis and treatment on multiple sclerosis (2018 Version)	RRMS(acute exacerbation phase)	T:38.86 ± 9.51 C: 37.65 ± 9.13	T: 4.16 ± 1.04 C: 4.28 ± 1.07
Lv (2015)	60 (30/30)	1983 Poser revised diagnostic criteria for MS	MS	T: 43 ± 8.4 C: 46.2 ± 9.0	T: 4.4 ± 3.5 C: 4.1 ± 3.2
Qian and Wang (2020)	60 (30/30)	McDonald criteria 2010	MS (acute exacerbation phase)	T: 33.47 ± 11.15 C: 34.07 ± 10.92	T: 19.38 ± 7.12 (months) C: 19.41 ± 6.11 (months)
Shi and Wang (2004)	38 (19/19)	1983 Poser revised diagnostic criteria for MS	RRMS + SPMS + PPMS + benign MS	T: 32.5 ± 11.6 C: 33.1 ± 10.2	T: 40.5 ± 37.6 (months) C: 41.3 ± 31.53 (months)
Wang et al. (2007)	58 (32/26)	1983 Poser revised diagnostic criteria for MS	MS	T: 34.5 C: 36.5	T: 2.7 C: 3.1
Wang et al. (2012)	60 (30/30)	McDonald criteria 2010	MS (acute exacerbation phase)	T: 58.7 ± 2.19 C: 59.8 ± 2.01	T: 59.5 ± 6.17 (months) C: 61.5 ± 6.21 (months)
Wu et al. (2020)	45 (22/23)	McDonald criteria 2010	MS	T: 35.4 C: 36.8	T: 4.3 ± 0.9 C: 3 ± 1.5
Yuan et al. (2025)	80 (40/40)	Chinese consensus on diagnosis and treatment on multiple sclerosis (2018 Version)	MS	T: 38.0 ± 4.5 C: 37.6 ± 8.3	T: 17.38 ± 7.02 (months) C: 18.27 ± 7.22 (months)
Zhao (2013)	36 (18/18)	—	MS	T: 40.2 C: 40.5	T: 25 (months) C: 22 (months)
Zhao et al. (2023)	90 (45/45)	Chinese consensus on diagnosis and treatment on multiple sclerosis (2018 Version)	MS	T: 43.28 ± 6.89 C: 43.76 ± 6.94	T: 4.45 ± 1.06 C: 4.58 ± 1.07
Zhang (2021)	84 (42/42)	Chinese consensus on diagnosis and treatment on multiple sclerosis (2018 Version)	MS	T: 32.76 ± 3.12 C: 32.15 ± 3.06	T: 1.12 ± 0.37 C: 1.06 ± 0.41
Zhang and Zhang (2006)	60 (30/30)	5th edition of Neurology	RRMS + SPMS + PPMS + benign MS	T: 33.41 ± 10.52 C: 31.93 ± 12.31	T: 17.32 ± 11.32 (months) C: 16.82 ± 12.14 (months)

**TABLE 2:** Intervention and outcomes of included studies.

First author, public year	Interventions	Comparisons	Course of treatment	TCM function(s) of CHM	Ingredients of CHM formula	Outcomes
Ayoobi et al. (2019)	*Achillea millefolium*	Placebo	12 months	—	• *Achillea millefolium* L. [Asteraceae] • Specifications: 250/500 mg per capsule	EDSS; ARR; Annual relapse frequency; MSFC; MRI
Bi (2016)	*Qishen Huanwu* capsule (2 capsules, bid) + CT	Placebo + CT	2 years	Tonify qi and activate blood, dispel stasis and unblock the channels	• Astragalus mongholicus Bunge [Fabaceae; Astragali radix] 120 g • Pseudostellaria heterophylla (Miq.) Pax [Caryophyllaceae; Pseudostellariae radix] 80 g • Prunus persica (L.) Batsch [Rosaceae; Persicae semen] 40 g • Carthamus tinctorius L. [Asteraceae; Carthami flos] 48 g • Angelica sinensis (Oliv.) Diels [Apiaceae; Angelicae sinensis radix] 40 g • Paeonia veitchii Lynch [Paeoniaceae; Paeoniae radix rubra] 48 g • Conioselinum anthriscoides ‘Chuanxiong’ [Apiaceae; Chuanxiong rhizoma] 40 g • Scrophularia ningpoensis Hemsl. [Scrophulariaceae; Scrophulariae radix] 48 g • Haliotis diversicolor Reeve [Haliotidae; Haliotidis concha] 48 g • Uncaria rhynchophylla (Miq.) Miq. ex Havil. [Rubiaceae; Uncariae ramulus cum uncis] 48 g • Ophiopogon japonicus (Thunb.) Ker Gawl. [Asparagaceae; Ophiopogonis radix] 40 g • Achyranthes bidentata Blume [Amaranthaceae; Achyranthis bidentatae radix] 40 g • Rheum palmatum L. [Polygonaceae; Rhei radix et rhizoma] 40 g • Forsythia suspensa (Thunb.) Vahl [Oleaceae; Forsythiae fructus] 48 g • Lumbricus [Oligochaeta; Lumbrici] 40 g • Whitmania pigra Whitman [Hirudinidae; Hirudo] 40 g • Arisaema amurense Maxim. [Araceae; arisaema cum bile] 60 g • Pinellia ternata (Thunb.) Makino [Araceae; Pinelliae rhizoma praeparatum] 32 g • Buthus martensii Karsch [Scorpionidae; Scorpio] 24 g • Specification: 0.4 g per capsule, equivalent to 3 g of crude herb	EDSS; ARR; MRI
Chen and Fan (2016)	*Bushen Yisui* decoctioin (1 dose/d) + CT	CT	3 months	Tonify kidneys and replenish essence, clear heat and transform phlegm, activate blood and unblock the channels	• Rehmannia glutinosa (Gaertn.) DC. [Orobanchaceae; Rehmanniae radix praeparata] 15 g • Rehmannia glutinosa (Gaertn.) DC. [Orobanchaceae; Rehmanniae radix] 15 g • Rheum palmatum L. or Rheum officinale Baill. [Polygonaceae; Rhei radix et rhizoma] 3 g • Polygonum multiflorum Thunb. [Polygonaceae; Polygoni multiflori radix] 12 g • Fritillaria thunbergii Miq. [Liliaceae; Fritillariae thunbergii bulbus] 9 g • Whitmania pigra Whitman [Hirudinidae; Hirudo] 3 g • Buthus martensii Karsch [Scorpionidae; Scorpio] 3 g • Gastrodia elata Blume [Orchidaceae; Gastrodiae rhizoma] 5 g • Forsythia suspensa (Thunb.) Vahl [Oleaceae; Forsythiae fructus] 9 g • Leonurus japonicus Houtt. [Lamiaceae; Leonuri herba] 15 g	MFIS
Chen et al. (2006)	*Guilu Yisui* capsule (8 capsules, tid) + CT	CT	3 months	Nourish essence and blood, tonify marrow and promote communication of the governor vessel, activate blood and unblock the channels	• Panax ginseng C.A.Mey. [Araliaceae; Ginseng radix et rhizoma] • Cervus nippon Temminck or *Cervus elaphus* Linnaeus [Cervidae; Cervi cornu pantotrichum] • *Chinemys reevesii* (Gray) [Geoemydidae; Testudinis plastra] • Cuscuta chinensis Lam. [Convolvulaceae; Cuscutae semen] • Polygonum multiflorum Thunb. [Polygonaceae; Polygoni multiflori radix] • Lycium barbarum L. [Solanaceae; Lycii fructus] • Buthus martensii Karsch [Scorpionidae; Scorpio] • Spatholobus suberectus Dunn [Fabaceae; Spatholobi caulis] • Specification: 0.4 g per capsule	EDSS; Clinical effect; Annual relapse frequency; Neurological signs score; Clinical symptom scores
Dong (2014)	*Shenqi Yangsui* formula (1 dose/d) + CT	CT	12 weeks	Clear heat and detoxify, warm the spleen and tonify the kidneys, transform phlegm and dispel stasis	• Astragalus mongholicus Bunge [Fabaceae; Astragali radix] 30 g • Epimedium brevicornum Maxim. [Berberidaceae; Epimedii herba] 30 g • Morinda officinalis How [Rubiaceae; Morindae officinalis radix] 30 g • Acorus tatarinowii Schott [Acoraceae; Acori tatarinowii rhizoma] 30 g • Paris polyphylla Smith [Melanthiaceae; Paridis rhizoma] 30 g • Rehmannia glutinosa (Gaertn.) DC. [Orobanchaceae; Rehmanniae radix praeparata] 25 g • Ligustrum lucidum W.T.Aiton [Oleaceae; Ligustri lucidi fructus] 25 g • Cuscuta chinensis Lam. [Convolvulaceae; Cuscutae semen] 20 g • Codonopsis pilosula (Franch.) Nannf. [Campanulaceae; Codonopsis radix] 20 g • Bombyx batryticatus [Bombycidae; Bombyx batryticatus] 20 g • Isatis indigotica Fort. [Brassicaceae; Isatidis folium] 20 g • Serissa japonica (Thunb.) Thunb. ex Tanaka [Rubiaceae; Serissae herba] 20 g • Whitmania pigra Whitman [Hirudinidae; Hirudo] 8 g	Clinical effect;
Duan (2013)	*Yiqi Huoxue Huatan* decoction (1 dose/d)+ CT	CT	1 month	Replenishing qi, activating blood, eliminating the Phlegm	• Achyranthes bidentata Blume [Amaranthaceae; Achyranthis bidentatae radix] 12 g • Astragalus mongholicus Bunge [Fabaceae; Astragali radix] 25 g • Pinellia ternata (Thunb.) Makino [Araceae; Pinelliae rhizoma praeparatum] 12 g • Citrus reticulata Blanco [Rutaceae; Citri reticulatae pericarpium] 20 g • Arisaema cum bile [Araceae; Arisaematis cum bile tuber] 15 g • Bombyx batryticatus [Bombycidae; Bombyx batryticatus] 5 g • Angelica sinensis (Oliv.) Diels [Apiaceae; Angelicae sinensis radix] 10 g • Paeonia veitchii Lynch [Paeoniaceae; Paeoniae radix rubra] 10 g • Conioselinum anthriscoides ‘Chuanxiong’ [Apiaceae; Chuanxiong rhizoma] 5 g • Coix lacryma-jobi L. var. ma-yuen (Roman.) Stapf [Poaceae; Coicis semen] 15 g • Glycyrrhiza uralensis Fisch. [Fabaceae; Glycyrrhizae radix et rhizoma] 10 g • Lumbricus [Oligochaeta; Lumbrici] 10 g • Poria cocos (Schw.) Wolf [Polyporaceae; Poria] 15 g • Bambusa tuldoides Munro [Poaceae; Bambusae caulis in taeniam] 15 g • Prunus persica (L.) Batsch [Rosaceae; Persicae semen] 10 g	EDSS; Clinical effect
Fan et al. (2006)	*Erhuang* formula (1 dose/d) + CT	CT	4 weeks	Nourish yin and tonify kidneys, transform phlegm and activate blood	• Rehmannia glutinosa (Gaertn.) DC. [Orobanchaceae; Rehmanniae radix praeparata] 15 g • Rehmannia glutinosa (Gaertn.) DC. [Orobanchaceae; Rehmanniae radix] 15 g • Rheum palmatum L. or Rheum officinale Baill. [Polygonaceae; Rhei radix et rhizoma] 3 g • Polygonum multiflorum Thunb. [Polygonaceae; Polygoni multiflori radix] 12 g • Fritillaria thunbergii Miq. [Liliaceae; Fritillariae thunbergii bulbus] 9 g • Whitmania pigra Whitman [Hirudinidae; Hirudo] 3 g • Buthus martensii Karsch [Scorpionidae; Scorpio] 3 g • Gastrodia elata Blume [Orchidaceae; Gastrodiae rhizoma] 5 g • Forsythia suspensa (Thunb.) Vahl [Oleaceae; Forsythiae fructus] 9 g • Leonurus japonicus Houtt. [Lamiaceae; Leonuri herba] 15 g	Annualized relapse frequency
Fan et al. (2018)	*Bushen Yisui* capsule (6 capsules, tid) + CT	Placebo + CT	3 months	Tonify the kidneys and enrich the marrow	• Rehmannia glutinosa (Gaertn.) DC. [Orobanchaceae; Rehmanniae radix praeparata] 15 g • Rehmannia glutinosa (Gaertn.) DC. [Orobanchaceae; Rehmanniae radix] 15 g • Rheum palmatum L. or Rheum officinale Baill. [Polygonaceae; Rhei radix et rhizoma] 3 g • Polygonum multiflorum Thunb. [Polygonaceae; Polygoni multiflori radix] 12 g • Fritillaria thunbergii Miq. [Liliaceae; Fritillariae thunbergii bulbus] 9 g • Whitmania pigra Whitman [Hirudinidae; Hirudo] 3 g • Buthus martensii Karsch [Scorpionidae; Scorpio] 3 g • Gastrodia elata Blume [Orchidaceae; Gastrodiae rhizoma] 5 g • Forsythia suspensa (Thunb.) Vahl [Oleaceae; Forsythiae fructus] 9 g • Leonurus japonicus Houtt. [Lamiaceae; Leonuri herba] 15 g • Specification: 0.5g/capsule	EDSS; Clinical effect; Scores of TCM syndrome
Gao et al. (2008)	*Dihuang Heji* capsule (3 capsules, tid) + CT	CT	3 weeks	Nourish the liver and kidneys, promote blood circulation and unblock collaterals	• Rehmannia glutinosa (Gaertn.) DC. [Orobanchaceae; Rehmanniae radix praeparata] • Cornus officinalis Siebold & Zucc. [Cornaceae; Corni fructus] • Ophiopogon japonicus (Thunb.) Ker Gawl. [Asparagaceae; Ophiopogonis radix] • Cistanche deserticola Y.C.Ma [Orobanchaceae; Cistanches herba] • Paeonia lactiflora Pall. [Paeoniaceae; Paeoniae radix alba] • Acorus tatarinowii Schott [Acoraceae; Acori tatarinowii rhizoma] • Arisaema cum bile [Araceae; Arisaematis cum bile tuber] • Lumbricus [Oligochaeta; Lumbrici] • Curcuma aromatica Salisb. or Curcuma wenyujin Y.H.Chen & C.Ling [Zingiberaceae; Curcumae radix] • Bombyx batryticatus [Bombycidae; Bombyx batryticatus] • Specification: Each capsule contains 1.5 g of crude herb	EDSS; Annual relapse frequency
Jie (2024)	*Yisui* granules (7.5g, bid) + CT	CT	3 months	Tonify the kidneys and benefit Qi, nourish Yin and tonify Yang	• Rehmannia glutinosa (Gaertn.) DC. [Orobanchaceae; Rehmanniae radix praeparata] • Lycium barbarum L. [Solanaceae; Lycii fructus] • Salvia miltiorrhiza Bunge [Lamiaceae; Salviae miltiorrhizae radix et rhizoma] • Morinda officinalis How [Rubiaceae; Morindae officinalis radix] • Cornus officinalis Siebold & Zucc. [Cornaceae; Corni fructus] • Paeonia suffruticosa Andrews [Paeoniaceae; Moutan cortex] • Astragalus mongholicus Bunge [Fabaceae; Astragali radix] • Cercis chinensis Bunge [Fabaceae; Cercidis chinensis flos] • Strychnos nux-vomica L. [Loganiaceae; Strychni semen pulvis] • Cordyceps sinensis (Berk.) Sacc. [Clavicipitaceae; Cordyceps] • Angelica sinensis (Oliv.) Diels [Apiaceae; Angelicae sinensis radix] • Ligusticum chuanxiong Hort. [Apiaceae; Chuanxiong rhizoma] • Cervus nippon Temminck or *Cervus elaphus* Linnaeus [Cervidae; Cervi cornu pantotrichum] • Polygonatum sibiricum Redouté [Asparagaceae; Polygonati rhizoma] • Dioscorea oppositifolia L. or Dioscorea opposita Thunb. [Dioscoreaceae; Dioscoreae rhizoma] • Spatholobus suberectus Dunn [Fabaceae; Spatholobi caulis] • Panax ginseng C.A.Mey. [Araliaceae; Ginseng radix et rhizoma] • Specification: 7.5 g per bag	EDSS; Pharmacodynamic biomarkers (IFN-γ, MMP-9, IL-12p40, TGF-β1,S-100,Tau, serum and cerebrospinal fluid T lymphocyte subsets (CD4^+^, CD8^+^ and CD4+/CD8+))
Li (2012)	*Jianpi Bushen Tongluo Jiedu* formula (1 dose/d) + CT	CT	3 months	Tonify the kidneys and strengthen the spleen, replenish essence and enrich the marrow, promote urination and resolve phlegm, invigorate blood, unblock the meridians, and detoxify	• Epimedium brevicornum Maxim. [Berberidaceae; Epimedii herba] 15 g • Morinda officinalis How [Rubiaceae; Morindae officinalis radix] 15 g • Astragalus mongholicus Bunge [Fabaceae; Astragali radix] 30 g • Cuscuta chinensis Lam. [Convolvulaceae; Cuscutae semen] 15 g • Atractylodes macrocephala Koidz. [Asteraceae; Atractylodis macrocephalae rhizoma] 15 g • Alisma plantago-aquatica subsp. orientale (Sam.) Sam. [Alismataceae; Alismatis rhizoma] 15 g • Ligusticum chuanxiong Hort. [Apiaceae; Chuanxiong rhizoma] 15 g • Gleditsia sinensis Lam. [Fabaceae; Gleditsiae sinensis spine] 10 g • Paris polyphylla Smith [Melanthiaceae; Paridis rhizoma] 15 g • Hedyotis diffusa Willd. [Rubiaceae; Hedyotis diffusa herba] 15 g • Buthus martensii Karsch [Scorpionidae; Scorpio] 10 g • Bombyx batryticatus [Bombycidae; Bombyx batryticatus] 10 g • Glycyrrhiza uralensis Fisch. [Fabaceae; Glycyrrhizae radix et rhizoma] 6 g	EDSS; Clinical symptom scores;
Li (2017)	*Tripterygium Wilfordii Polyglycosides* (1 mg/kg.d, bid) + CT	CT	3 months	Activate blood and unblock the channels, kill parasites and detoxify, dispel wind and eliminate dampness, reduce swelling and relieve pain	Tripterygium wilfordii Hook.f. [Celastraceae; Tripterygii wilfordii radix]	Clinical effect; Neurological signs score; Pharmacodynamic biomarkers (IL-6, IFN-γ)
Li and Yan (2017)	*Toufengling* decoction (1 dose/d) + CT	CT	3 months	—	• Glycyrrhiza uralensis Fisch. [Fabaceae; Glycyrrhizae radix et rhizoma] 6 g • Codonopsis pilosula (Franch.) Nannf. [Campanulaceae; Codonopsis radix] 30 g • Atractylodes macrocephala Koidz. [Asteraceae; Atractylodis macrocephalae rhizoma] 15 g • Cistanche deserticola Y.C.Ma [Orobanchaceae; Cistanches herba] 20 g • Epimedium brevicornum Maxim. [Berberidaceae; Epimedii herba] 20 g • Rehmannia glutinosa (Gaertn.) DC. [Orobanchaceae; Rehmanniae radix] 15 g • Salvia miltiorrhiza Bunge [Lamiaceae; Salviae miltiorrhizae radix et rhizoma] 15 g • Poria cocos (Schw.) Wolf [Polyporaceae; Poria] 15 g • Glycyrrhiza uralensis Fisch. [Fabaceae; Glycyrrhizae radix et rhizoma] 6 g • Alpinia oxyphylla Miq. [Zingiberaceae; Alpiniae oxyphyllae fructus] 20 g • Curcuma aromatica Salisb. or Curcuma wenyujin Y.H.Chen & C.Ling [Zingiberaceae; Curcumae radix] 15 g • Ligusticum chuanxiong Hort. [Apiaceae; Chuanxiong rhizoma] 15 g	EDSS; Annual relapse frequency; Clinical effect
Li and Zhao (2012)	Chinese medicine decoction (1 dose/d) + CT	CT	3 months	Nourish the spleen and kidneys, detoxify, remove blood stasis, dispel dampness, dispel wind and unblock collaterals	• Astragalus mongholicus Bunge [Fabaceae; Astragali radix] 30 g • Codonopsis pilosula (Franch.) Nannf. [Campanulaceae; Codonopsis radix] 20 g • Epimedium brevicornum Maxim. [Berberidaceae; Epimedii herba] 30 g • Cornus officinalis Siebold & Zucc. [Cornaceae; Corni fructus] 20 g • Morinda officinalis How [Rubiaceae; Morindae officinalis radix] 20 g • Ligustrum lucidum W.T.Aiton [Oleaceae; Ligustri lucidi fructus] 20 g • Cuscuta chinensis Lam. [Convolvulaceae; Cuscutae semen] 30 g • Buthus martensii Karsch [Scorpionidae; Scorpio] 10 g • Bombyx batryticatus [Bombycidae; Bombyx batryticatus] 15 g • Paeonia lactiflora Pall. [Paeoniaceae; Paeoniae radix rubra] 25 g • Curcuma zedoaria (Christm.) Roscoe [Zingiberaceae; Curcumae rhizoma] 30 g • Pinellia ternata (Thunb.) Makino [Araceae; Pinelliae rhizoma praeparatum] 10 g Arisaema cum bile [Araceae; Arisaematis cum bile tuber] 12 g • Alisma plantago-aquatica subsp. orientale (Sam.) Sam. [Alismataceae; Alismatis rhizoma] 30 g • Paris polyphylla Smith [Melanthiaceae; Paridis rhizoma] 30 g Serissa japonica (Thunb.) Thunb. ex Tanaka [Rubiaceae; Serissae herba] 30 g	EDSS; Clinical effect
Li et al. (2019)	*Yishen Busui Tongluo* decoction (200mL, bid) + CT	CT	12 weeks	Tonify kidneys and replenish marrow, dispel stasis and unblock the channels	• Astragalus mongholicus Bunge [Fabaceae; Astragali radix] 30 g • Epimedium brevicornum Maxim. [Berberidaceae; Epimedii herba] 30 g • Coix lacryma-jobi L. var. ma-yuen (Roman.) Stapf [Poaceae; Coicis semen] 25 g • Alisma plantago-aquatica subsp. orientale (Sam.) Sam. [Alismataceae; Alismatis rhizoma] 25 g • Cuscuta chinensis Lam. [Convolvulaceae; Cuscutae semen] 20 g • Codonopsis pilosula (Franch.) Nannf. [Campanulaceae; Codonopsis radix] 20 g • Rehmannia glutinosa (Gaertn.) DC. [Orobanchaceae; Rehmanniae radix praeparata] 20 g • Paeonia lactiflora Pall. [Paeoniaceae; Paeoniae radix rubra] 20 g • Curcuma zedoaria (Christm.) Roscoe [Zingiberaceae; Curcumae rhizoma] 20 g • Hedyotis diffusa Willd. [Rubiaceae; Hedyotis diffusa herba] 15 g • Ligusticum chuanxiong Hort. [Apiaceae; Chuanxiong rhizoma] 15 g • Paris polyphylla Smith [Melanthiaceae; Paridis rhizoma] 15 g • Sparganium stoloniferum Buch.-Ham. [Typhaceae; Sparganii rhizoma] 10 g • Bombyx batryticatus [Bombycidae; Bombyx batryticatus] 10 g • Buthus martensii Karsch [Scorpionidae; Scorpio] 10 g • Glycyrrhiza uralensis Fisch. [Fabaceae; Glycyrrhizae radix et rhizoma] 6 g	Clinical effect; MFIS; Pharmacodynamic biomarkers (IL-6, IFN-γ, IL-10, HMGB1)
Li et al. (2024)	*Si Miao* pills (6g, bid) + CT	Placebo + CT	12 weeks	Clear heat and dry dampness	• Phellodendron amurense Rupr. [Rutaceae; Phellodendri cortex] • Atractylodes lancea (Thunb.) DC. [Asteraceae; Atractylodis rhizoma] • Coix lacryma-jobi L. var. ma-yuen (Roman.) Stapf [Poaceae; Coicis semen] • Achyranthes bidentata Blume [Amaranthaceae; Achyranthis bidentatae radix] • Specification: 6 g per bag	EDSS; ARR; LOTCA-II; Scores of TCM syndrome
Li et al. (2025)	*Si Miao* pills (6g, bid) + CT	CT	4 weeks	Clear heat and dry dampness	• Phellodendron amurense Rupr. [Rutaceae; Phellodendri cortex] • Atractylodes lancea (Thunb.) DC. [Asteraceae; Atractylodis rhizoma] • Coix lacryma-jobi L. var. ma-yuen (Roman.) Stapf [Poaceae; Coicis semen] • Achyranthes bidentata Blume [Amaranthaceae; Achyranthis bidentatae radix] • Specification: 6 g per bag	EDSS; Clinical effect; Pharmacodynamic biomarkers (Th17, Treg, Th17/Treg, IL-17, IL-23, TNF-α); Adverse effect
Lv (2015)	*Yishen-Bushui-Tongluo* decoction (1 dose/d) + CT	CT	3 months	Warm and tonify the spleen and kidneys, nourish essence and marrow, resolve phlegm, eliminate stasis, and improve circulation	• Rehmannia glutinosa (Gaertn.) DC. [Orobanchaceae; Rehmanniae radix praeparata] 25 g • Rehmannia glutinosa (Gaertn.) DC. [Orobanchaceae; Rehmanniae radix] 25 g • Poria cocos (Schw.) Wolf [Polyporaceae; Poria] 20 g • Paeonia suffruticosa Andrews [Paeoniaceae; Moutan cortex] 10 g • Aconitum carmichaelii Debeaux [Ranunculaceae; Aconiti lateralis radix praeparata] 5 g • Epimedium brevicornum Maxim. [Berberidaceae; Epimedii herba] 30 g • Cistanche deserticola Y.C.Ma [Orobanchaceae; Cistanches herba] 15 g • Achyranthes bidentata Blume [Amaranthaceae; Achyranthis bidentatae radix] 20 g • Astragalus mongholicus Bunge [Fabaceae; Astragali radix] 30 g • Pueraria montana var. lobata (Willd.) Maesen & S.M.Almeida [Fabaceae; Puerariae lobatae radix] 15 g • Ligusticum chuanxiong Hort. [Apiaceae; Chuanxiong rhizoma] 15 g • Cuscuta chinensis Lam. [Convolvulaceae; Cuscutae semen] 20 g • Lumbricus [Oligochaeta; Lumbrici] 8 g • Bombyx batryticatus [Bombycidae; Bombyx batryticatus] 20 g	EDSS; Annual relapse frequency; Clinical effect; Neurological signs score; Clinical symptom scores
Qian and Wang (2020)	*Ziyin Guben* granules (6g, tid) + CT	CT	27 days	Nourish the liver and kidneys, strengthen the spleen and stomach, soothe the liver and clear heat	• Rehmannia glutinosa (Gaertn.) DC. [Orobanchaceae; Rehmanniae radix praeparata] • Rehmannia glutinosa (Gaertn.) DC. [Orobanchaceae; Rehmanniae radix] • Angelica sinensis (Oliv.) Diels [Apiaceae; Angelicae sinensis radix] • *Chinemys reevesii* (Gray) [Geoemydidae; Testudinis plastra] • Paeonia lactiflora Pall. [Paeoniaceae; Paeoniae radix alba] • Adenophora stricta Miq. or Adenophora triphylla (Thunb.) A.DC. [Campanulaceae; Adenophorae radix] • Ophiopogon japonicus (Thunb.) Ker Gawl. [Asparagaceae; Ophiopogonis radix] • Lycium barbarum L. [Solanaceae; Lycii fructus] • Citrus reticulata Blanco [Rutaceae; Citri reticulatae pericarpium] • Zingiber officinale Roscoe [Zingiberaceae; Zingiberis rhizoma] • Phellodendron amurense Rupr. or Phellodendron chinense C.K.Schneid. [Rutaceae; Phellodendri cortex] • Anemarrhena asphodeloides Bunge [Asparagaceae; Anemarrhenae rhizoma] • Cynomorium songaricum Rupr. [Cynomoriaceae; Cynomorii herba] • Achyranthes bidentata Blume [Amaranthaceae; Achyranthis bidentatae radix] • Melia toosendan Siebold & Zucc. [Meliaceae; Toosendan fructus] • Specification: 6 g per bag	EDSS; Clinical effect; Neurological signs score; Clinical symptom scores
Shi and Wang (2004)	*Jiannao Gusui decoction* (1 dose/d) + CT	CT	3 months	Warm and tonify kidney yang, tonify qi, invigorate blood, and unblock the meridians	• Codonopsis pilosula (Franch.) Nannf. [Campanulaceae; Codonopsis radix] 30 g • Atractylodes macrocephala Koidz. [Asteraceae; Atractylodis macrocephalae rhizoma] 15 g • Poria cocos (Schw.) Wolf [Polyporaceae; Poria] 15 g • Glycyrrhiza uralensis Fisch. [Fabaceae; Glycyrrhizae radix et rhizoma] 6 g • Salvia miltiorrhiza Bunge [Lamiaceae; Salviae miltiorrhizae radix et rhizoma] 15 g • Cistanche deserticola Y.C.Ma [Orobanchaceae; Cistanches herba] 20 g • Epimedium brevicornum Maxim. [Berberidaceae; Epimedii herba] 20 g • Alpinia oxyphylla Miq. [Zingiberaceae; Alpiniae oxyphyllae fructus] 20 g • Curcuma aromatica Salisb. [Zingiberaceae; Curcumae radix] 15 g • Rehmannia glutinosa (Gaertn.) DC. [Orobanchaceae; Rehmanniae radix] 15 g • Ligusticum chuanxiong Hort. [Apiaceae; Chuanxiong rhizoma] 15 g	EDSS; Annual relapse frequency; Clinical effect; Neurological signs score; Clinical symptom scores
Wang et al. (2007)	*Jiannao Bushen Gusui* formula (1 dose/d) + CT	CT	3 months	Tonify the liver and kidneys, tonify qi and strengthen the spleen, invigorate blood and remove stasis, clear heat, resolve dampness, and unblock the meridians	• Atractylodes macrocephala Koidz. [Asteraceae; Atractylodis macrocephalae rhizoma] 15 g • Glycyrrhiza uralensis Fisch. [Fabaceae; Glycyrrhizae radix et rhizoma] 6 g • Epimedium brevicornum Maxim. [Berberidaceae; Epimedii herba] 15 g • Poria cocos (Schw.) Wolf [Polyporaceae; Poria] 15 g • Cistanche deserticola Y.C.Ma [Orobanchaceae; Cistanches herba] 15 g • Rehmannia glutinosa (Gaertn.) DC. [Orobanchaceae; Rehmanniae radix] 15 g • Polygonum multiflorum Thunb. [Polygonaceae; Polygoni multiflori radix praeparata] 15 g • Curcuma aromatica Salisb. [Zingiberaceae; Curcumae radix] 10 g • Salvia miltiorrhiza Bunge [Lamiaceae; Salviae miltiorrhizae radix et rhizoma] 20 g • Ligusticum chuanxiong Hort. [Apiaceae; Chuanxiong rhizoma] 20 g	EDSS; Clinical effect
Wang et al. (2012)	*Dihuang Yinzi* decoction (1 dose/d) + CT	CT	3 months	Nourish kidney yin, supplement kidney yang, open orifices and resolve phlegm	• Rehmannia glutinosa (Gaertn.) DC. [Orobanchaceae; Rehmanniae radix praeparata] 20 g • Morinda officinalis How [Rubiaceae; Morindae officinalis radix] 10 g • Cornus officinalis Sieb. et Zucc. [Cornaceae; Corni fructus] 12 g • Dendrobium nobile Lindl. [Orchidaceae; Dendrobii caulis] 9 g • Cistanche deserticola Y.C.Ma [Orobanchaceae; Cistanches herba] 10 g • Aconitum carmichaelii Debeaux [Ranunculaceae; Aconiti lateralis radix praeparata] 6 g • Schisandra chinensis (Turcz.) Baill. [Schisandraceae; Schisandrae chinensis fructus] 9 g • Cinnamomum cassia (L.) J.Presl [Lauraceae; Cinnamomi cortex] 3 g • Poria cocos (Schw.) Wolf [Polyporaceae; Poria] 12 g • Ophiopogon japonicus (Thunb.) Ker Gawl. [Asparagaceae; Ophiopogonis radix] 9 g • Acorus tatarinowii Schott [Acoraceae; Acori tatarinowii rhizoma] 30 g • Polygala tenuifolia Willd. [Polygalaceae; Polygalae radix] 10 g	EDSS; Clinical effect; Annual relapse frequency; MRI
Wu et al. (2020)	*Nourishing Liver and Kidney* recipe (100mL, bid) + CT	CT	1 month	Nourish the liver and benefit the kidneys, strengthen the tendons and bones	• Eucommia ulmoides Oliv. [Eucommiaceae; Eucommiae cortex] 15 g • Dioscorea opposita Thunb. [Dioscoreaceae; Dioscoreae rhizoma] 15 g • Poria cocos (Schw.) Wolf [Polyporaceae; Poria] 15 g • Cornus officinalis Sieb. et Zucc. [Cornaceae; Corni fructus] 15 g • Phellodendron chinense Schneid. [Rutaceae; Phellodendri chinensis cortex] 8 g • *Cervus elaphus* Linnaeus [Cervidae; Cervi cornu colla] 15 g • Epimedium brevicornum Maxim. [Berberidaceae; Epimedii herba] 12 g • Boswellia sacra Flueck. [Burseraceae; Olibanum] 3 g • Commiphora myrrha (Nees) Engl. [Burseraceae; Myrrha] 3 g • Strychnos nux-vomica L. [Loganiaceae; Strychni semen praeparata] 0.1 g	EDSS; Clinical effect;
Yuan et al. (2025)	*Magui Zhuli* Decoction (220 mL, bid) + CT	CT	4 weeks	Relieves Lung Qi stagnation and expels wind, boosts vital energy and promotes blood circulation, and clears internal heat	• Ephedra sinica Stapf [Ephedraceae; Ephedrae Herba] 6 g • Cinnamomum cassia (L.) J. Presl [Lauraceae; Cinnamomi cassiae cortex] 10 g • Angelica sinensis (Oliv.) Diels [Apiaceae; Angelicae sinensis radix] 10 g • Panax ginseng C.A. Meyer [Araliaceae; Ginseng radix] 6 g • Gypsum Fibrosum [—; Gypsum Fibrosum] 20 g • Zingiber officinale Roscoe [Zingiberaceae; Zingiberis rhizoma] 9 g • Glycyrrhiza uralensis Fisch. ex DC. [Fabaceae; Liquiritiae radix] 9 g • Ligusticum chuanxiong Hort. [Apiaceae; Chuanxiong rhizoma] 9 g • Prunus armeniaca L. (seed) [Rosaceae; Armeniacae semen] 10 g • Phyllostachys nigra (Lodd. ex Lindl.) Munro [Poaceae; Bambusae caulis in taeniam] 20 mL	EDSS; Pharmacodynamic biomarkers (TNF-α, IL-1β, IFN-γ, IL-4, IL-17)
Zhao (2013)	*Bushen Gusui* pills (6 pills, tid) + CT	CT	3 months	Warm and tonify kidney yang, promote circulation and strengthen the marrow	• Epimedium brevicornum Maxim. [Berberidaceae; Epimedii herba] 15 g • Salvia miltiorrhiza Bunge [Lamiaceae; Salviae miltiorrhizae radix et rhizoma] 15 g • Cistanche deserticola Y.C.Ma [Orobanchaceae; Cistanches herba] 12 g • Curculigo orchioides Gaertn. [Hypoxidaceae; Curculiginis rhizoma] 10 g • Rehmannia glutinosa (Gaertn.) DC. [Orobanchaceae; Rehmanniae radix] 15 g • Polygonum multiflorum Thunb. [Polygonaceae; Polygoni multiflori radix praeparata] 15 g • Curcuma aromatica Salisb. [Zingiberaceae; Curcumae radix] 10 g • Specification: each pill contains 0.5 g of raw herb.	EDSS; Clinical effect; Neurological signs score; Clinical symptom scores
Zhao et al. (2023)	*Yishen Jiedu Tongluo* decoction (10g, qd) + CT	CT	4 weeks	Tonify the kidneys and transform turbid substances, detoxify and unblock the channels	• Rehmannia glutinosa (Gaertn.) DC. [Orobanchaceae; Rehmanniae radix praeparata] • Dioscorea hypoglauca Palib. [Dioscoreaceae; Dioscoreae hypoglaucae rhizoma] • Aconitum carmichaelii Debeaux [Ranunculaceae; Aconiti lateralis radix praeparata] • Notopterygium incisum Ting ex H.T.Chang [Apiaceae; Notopterygii rhizoma et radix] • Angelica sinensis (Oliv.) Diels [Apiaceae; Angelicae sinensis radix] • Anemarrhena asphodeloides Bunge [Asparagaceae; Anemarrhenae rhizoma] • Curcuma aromatica Salisb. [Zingiberaceae; Curcumae radix] • *Cervus elaphus* Linnaeus [Cervidae; Cervi cornu colla] • Acorus tatarinowii Schott [Acoraceae; Acori tatarinowii rhizoma] • Gardenia jasminoides Ellis [Rubiaceae; Gardeniae fructus] • Siegesbeckia orientalis L. [Asteraceae; Siegesbeckiae herba] • Glycyrrhiza uralensis Fisch. [Fabaceae; Glycyrrhizae radix et rhizoma] • Specification: 10 g per bag	EDSS; Clinical effect; Scores of TCM syndrome; MoCA; MMSE; Pharmacodynamic biomarkers (Th17, Treg, Th17/Treg)
Zhang (2021)	*Yisui* granules (7.5 g, bid) + CT	CT	3 months	Tonify the kidneys and boost qi, nourish yin and support yang	• Rehmannia glutinosa (Gaertn.) DC. [Orobanchaceae; Rehmanniae radix praeparata] • Lycium barbarum L. [Solanaceae; Lycii fructus] • Salvia miltiorrhiza Bunge [Lamiaceae; Salviae miltiorrhizae radix et rhizoma] • Morinda officinalis How [Rubiaceae; Morindae officinalis radix] • Cornus officinalis Siebold & Zucc. [Cornaceae; Corni fructus] • Paeonia suffruticosa Andrews [Paeoniaceae; Moutan cortex] • Astragalus mongholicus Bunge [Fabaceae; Astragali radix] • Cercis chinensis Bunge [Fabaceae; Cercidis chinensis flos] • Strychnos nux-vomica L. [Loganiaceae; Strychni semen pulvis] • Cordyceps sinensis (Berk.) Sacc. [Clavicipitaceae; Cordyceps] • Angelica sinensis (Oliv.) Diels [Apiaceae; Angelicae sinensis radix] • Ligusticum chuanxiong Hort. [Apiaceae; Chuanxiong rhizoma] • Cervus nippon Temminck or *Cervus elaphus* Linnaeus [Cervidae; Cervi cornu pantotrichum] • Polygonatum sibiricum Redouté [Asparagaceae; Polygonati rhizoma] • Dioscorea oppositifolia L. or Dioscorea opposita Thunb. [Dioscoreaceae; Dioscoreae rhizoma] • Spatholobus suberectus Dunn [Fabaceae; Spatholobi caulis] • Panax ginseng C.A.Mey. [Araliaceae; Ginseng radix et rhizoma] • Specification: 15 g per bag	EDSS; Scores of TCM syndrome
Zhang and Zhang (2006)	*Gusui Tongluo* formula (1 dose/d) + CT	CT	2 months	Tonify the kidneys and stabilize essence, resolve phlegm and dispel stasis, promote circulation and unblock the meridians	• Astragalus membranaceus (Fisch.) Bunge [Fabaceae; Astragali radix] 30 g • Codonopsis pilosula (Franch.) Nannf. [Campanulaceae; Codonopsis radix] 20 g • Rehmannia glutinosa (Gaertn.) DC. [Orobanchaceae; Rehmanniae radix praeparata] 20 g • Cuscuta chinensis Lam. [Convolvulaceae; Cuscutae semen] 20 g • Astragalus complanatus R.Br. [Fabaceae; Astragali complanati semen] 20 g • Epimedium brevicornum Maxim. [Berberidaceae; Epimedii herba] 30 g • Paeonia lactiflora Pall. [Paeoniaceae; Paeoniae radix alba] 25 g • Sparganium stoloniferum Buch.-Ham. [Typhaceae; Sparganii rhizoma] 12 g • Curcuma zedoaria (Christm.) Roscoe [Zingiberaceae; Curcumae rhizoma] 20 g • Alisma orientale (Sam.) Juzep. [Alismataceae; Alismatis rhizoma] 30 g • Coix lacryma-jobi var. ma-yuen [Poaceae; Coicis semen] 30 g • Buthus martensii Karsch [Buthidae; Scorpio] 10 g • *Bombyx mori* L. [Bombycidae; Bombyx batryticatus] 15 g	EDSS; Clinical effect; Neurological signs score; Clinical symptom scores

Abbreviations: EDSS, expanded disability status scale; ARR, annualized relapse rate; MRI, magnetic resonance imaging; MFIS, modified fatigue impact scale; MoCA, montreal cognitive assessment; MMSE, Mini-Mental State Examination; LOTCA-II, Loewenstein Occupational Therapy Cognitive Assessment-II; MSFC, multiple sclerosis functional composite; TCM, traditional chinese medicine; CT, conventional therapy; IFN-γ, interferon-γ; MMP-9, matrix metalloproteinase-9; IL-1β, interleukin-1β; IL-4, interleukin-4; IL-6, interleukin-6; IL-10, interleukin-10; IL-17, interleukin-17; IL-23, interleukin-23; HMGB1, high mobility group box-1, protein; IL-12p40, interleukin-12p40; TGF-β_1_, transforming growth factor-β_1_; TNF-α, tumor necrosis factor-α.

### 3.3 Risk of bias assessment

**FIGURE 2:**
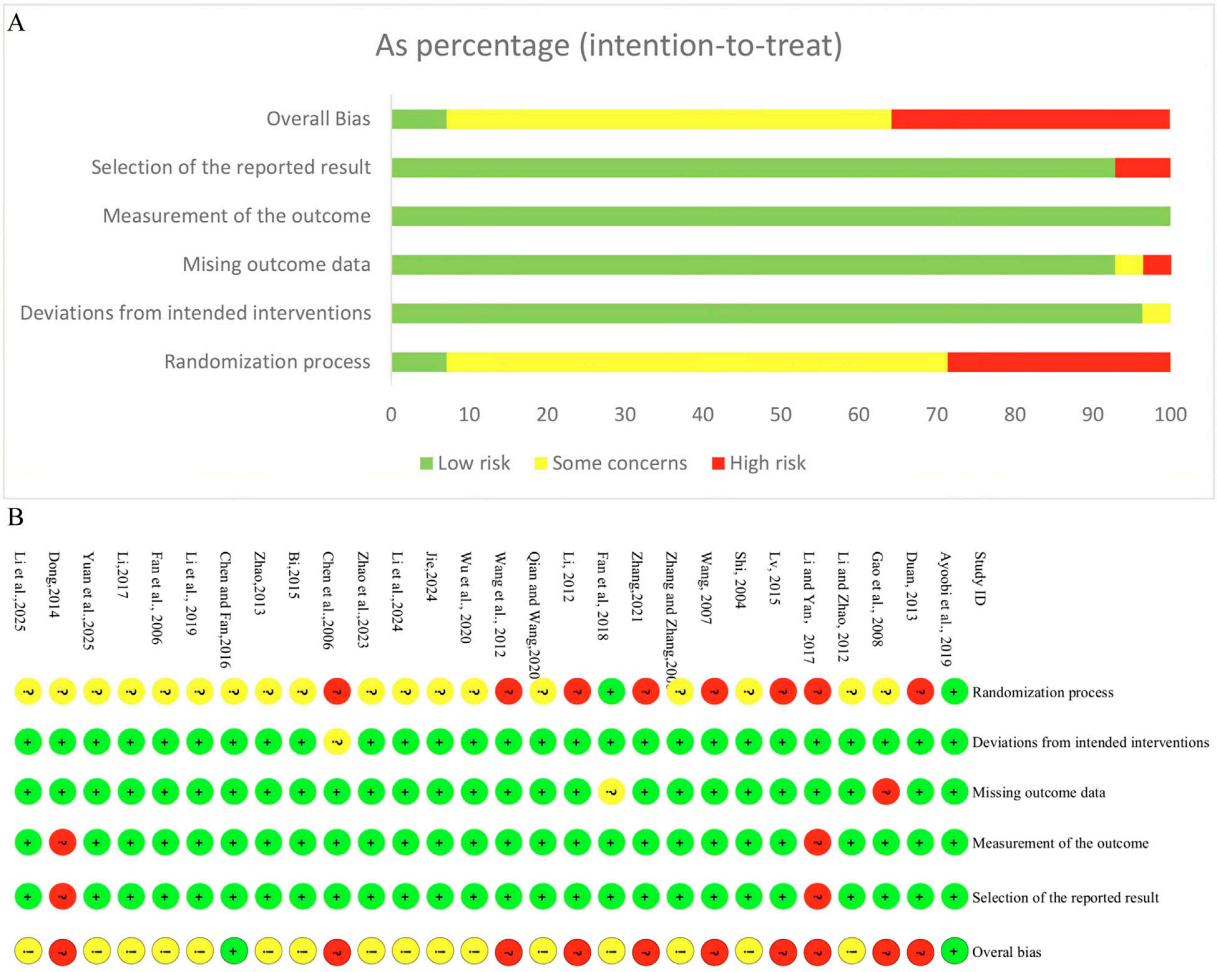
Risk of bias assessment for included RCTs. **(A)** Risk of bias graph for included RCTs. **(B)** Risk of bias summary for included RCTs.

The risk of bias assessment for the included RCTs is illustrated in Figure 2. In the domain of randomization process, only 7.1% of studies were judged as low risk, while 64.3% had some concerns, and 28.6% were assessed as high risk, mainly due to the use of inadequate sequence generation methods (such as hospital admission date, or the order of their visits) and insufficient reporting of allocation concealment. For deviations from intended interventions, 96.4% of studies were rated as low risk, indicating good adherence to the assigned interventions, with only 3.6% having some concerns. Regarding missing outcome data, 92.9% of studies were at low risk, 3.6% had some concerns, and 3.6% were judged as high risk due to incomplete outcome data. In the domain of measurement of the outcome, all studies (100%) were assessed as low risk. For selection of the reported result, 92.9% of studies were at low risk, while 7.1% were considered high risk because of potential selective reporting and lack of pre-specified analysis plans. Overall, only 7.1% of studies were rated as low risk of bias across all domains, while 57.1% had some concerns, and 35.7% were judged to be at high risk of bias.

### 3.4 Description of CHMs

A total of 107 botanical drugs were included in the 28 studies. The top 19 most frequently used botanical drugs were as follows: Rehmannia glutinosa (Gaertn.) DC. (Orobanchaceae; Rehmanniae radix praeparata), Angelica sinensis (Oliv.) Diels (Apiaceae; Angelicae sinensis radix), Polygonum multiflorum Thunb. (Polygonaceae; Polygoni multiflori radix), Epimedium brevicornum Maxim. (Berberidaceae; Epimedii herba), Cistanche deserticola Y.C.Ma (Orobanchaceae; Cistanches herba), Morinda officinalis How (Rubiaceae; Morindae officinalis radix), Cuscuta chinensis Lam. (Convolvulaceae; Cuscutae semen), Astragalus mongholicus Bunge (Fabaceae; Astragali radix), Glycyrrhiza uralensis Fisch. (Fabaceae; Glycyrrhizae radix et rhizome), Salvia miltiorrhiza Bunge (Lamiaceae; Salviae miltiorrhizae radix et rhizome), Cornus officinalis Siebold & Zucc. (Cornaceae; Corni fructus), Bombyx batryticatus (Bombycidae; Bombyx batryticatus), Buthus martensii Karsch (Scorpionidae; Scorpio), Ligusticum chuanxiong Hort. (Apiaceae; Chuanxiong rhizome), Salviae Miltiorrhizae Radix Et Rhizoma, Curcuma aromatica Salisb. or Curcuma wenyujin Y.H.Chen & C. Ling (Zingiberaceae; Curcumae radix), Poria cocos (Schw.) Wolf (Polyporaceae; Poria), Rehmannia glutinosa (Gaertn.) DC. (Orobanchaceae; Rehmanniae radix), Paeonia veitchii Lynch (Paeoniaceae; Paeoniae radix rubra). These drugs were used at least five times (Table 3) and encompassed 66 single botanicals (Supplementary Appendix 2).

**TABLE 3:** Top 19 botanical drugs and efficacy based on frequency of usage in the included studies.

Effect		Latin binomial with authority [Family; Pharmacopoeial drug name]	Chinese pinyin	Frequency
Tonifying Drugs	Blood-Tonifying Drugs	Rehmannia glutinosa (Gaertn.) DC. [Orobanchaceae; Rehmanniae radix praeparata]	Shu Di Huang	13
		Angelica sinensis (Oliv.) Diels [Apiaceae; Angelicae sinensis radix]	Dang Gui	7
		Polygonum multiflorum Thunb. [Polygonaceae; Polygoni multiflori radix]	Shou Wu	6
	Yang-Tonifying Drugs	Epimedium brevicornum Maxim. [Berberidaceae; Epimedii herba]	Yin Yang Huo	11
		Cistanche deserticola Y.C.Ma [Orobanchaceae; Cistanches herba]	Rou Cong Rong	7
		Morinda officinalis How [Rubiaceae; Morindae officinalis radix]	Ba Ji Tian	6
		Cuscuta chinensis Lam. [Convolvulaceae; Cuscutae semen]	Tu Si Zi	6
	Qi-Tonifying Drugs	Astragalus mongholicus Bunge [Fabaceae; Astragali radix]	Huang Qi	10
		Glycyrrhiza uralensis Fisch. [Fabaceae; Glycyrrhizae radix et rhizoma]	Gan Cao	8
		Salvia miltiorrhiza Bunge [Lamiaceae; Salviae miltiorrhizae radix et rhizoma]	Dang Shen	6
Astringent Drugs		Cornus officinalis Siebold & Zucc. [Cornaceae; Corni fructus]	Shan Zhu Yu	6
Liver-Calming and Wind-Soothing Drugs		Bombyx batryticatus [Bombycidae; Bombyx batryticatus]	Jiang Can	8
		Buthus martensii Karsch [Scorpionidae; Scorpio]	Quan Xie	8
Blood-Activating and Stasis-Dispelling Drugs		Ligusticum chuanxiong Hort. [Apiaceae; Chuanxiong rhizoma]	Chuan Xiong	11
		Salviae Miltiorrhizae Radix Et Rhizoma	Dan Shen	7
		ŸCurcuma aromatica Salisb. or Curcuma wenyujin Y.H.Chen & C.Ling [Zingiberaceae; Curcumae radix]	Yu Jin	6
Water-Draining and Dampness Transforming Drugs		Poria cocos (Schw.) Wolf [Polyporaceae; Poria]	Fu Lin	7
Heat-clearing and Blood-cooling Drugs		Rehmannia glutinosa (Gaertn.) DC. [Orobanchaceae; Rehmanniae radix]	Sheng Di Huang	9
		Paeonia veitchii Lynch [Paeoniaceae; Paeoniae radix rubra]	Chi Shao	5

### 3.5 Outcome measures

#### 3.5.1 EDSS

A total of 23 studies evaluated EDSS. Among these, 19 trials (Chen et al., 2006; Duan, 2013; Gao et al., 2008; Jie, 2024; Li and Yan, 2017; Li and Zhao, 2012; Li, 2012; Li et al., 2025; Lv, 2015; Qian and Wang, 2020; Shi and Wang, 2004; Wang et al., 2007; Wang et al., 2012; Wu et al., 2020; Yuan et al., 2025; Zhang and Zhang, 2006; Zhang, 2021; Zhao et al., 2023; Zhao, 2013) conducted conventional treatment add-on therapy. The qualitative summary replaced meta-analysis due to substantial heterogeneity (I^2^ = 99% in the pooling analysis of EDSS). Among the 19 trials, 18 reported that the combination of CHM with conventional therapy resulted in a significantly greater reduction in EDSS scores compared to conventional therapy alone. One trial (Li and Yan, 2017) also observed lower EDSS scores in the CHM group; however, there was no statistically significant difference between treatment and control groups.

**FIGURE 3:**
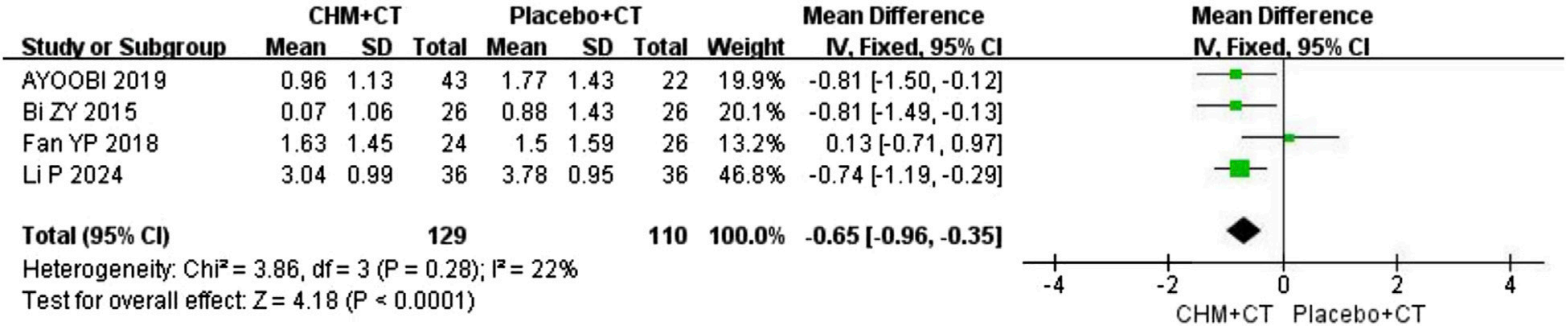
Effect of CHM with CT versus placebo with CT on EDSS.

Additionally, four trials (Ayoobi et al., 2019; Bi, 2016; Fan et al., 2018; Li et al., 2024) compared CHM with a placebo. A fixed effect model was used due to the low heterogeneity (Cochrane Q test = 3.86, I^2^ = 22%, *P* = 0.28). The pooled results revealed that CHM therapy could decrease EDSS scores compared to placebo (MD = −0.65; 95% CI: −0.96, −0.35; *P* < 0.0001) (Figure 3).

#### 3.5.2 Relapse

**FIGURE 4:**
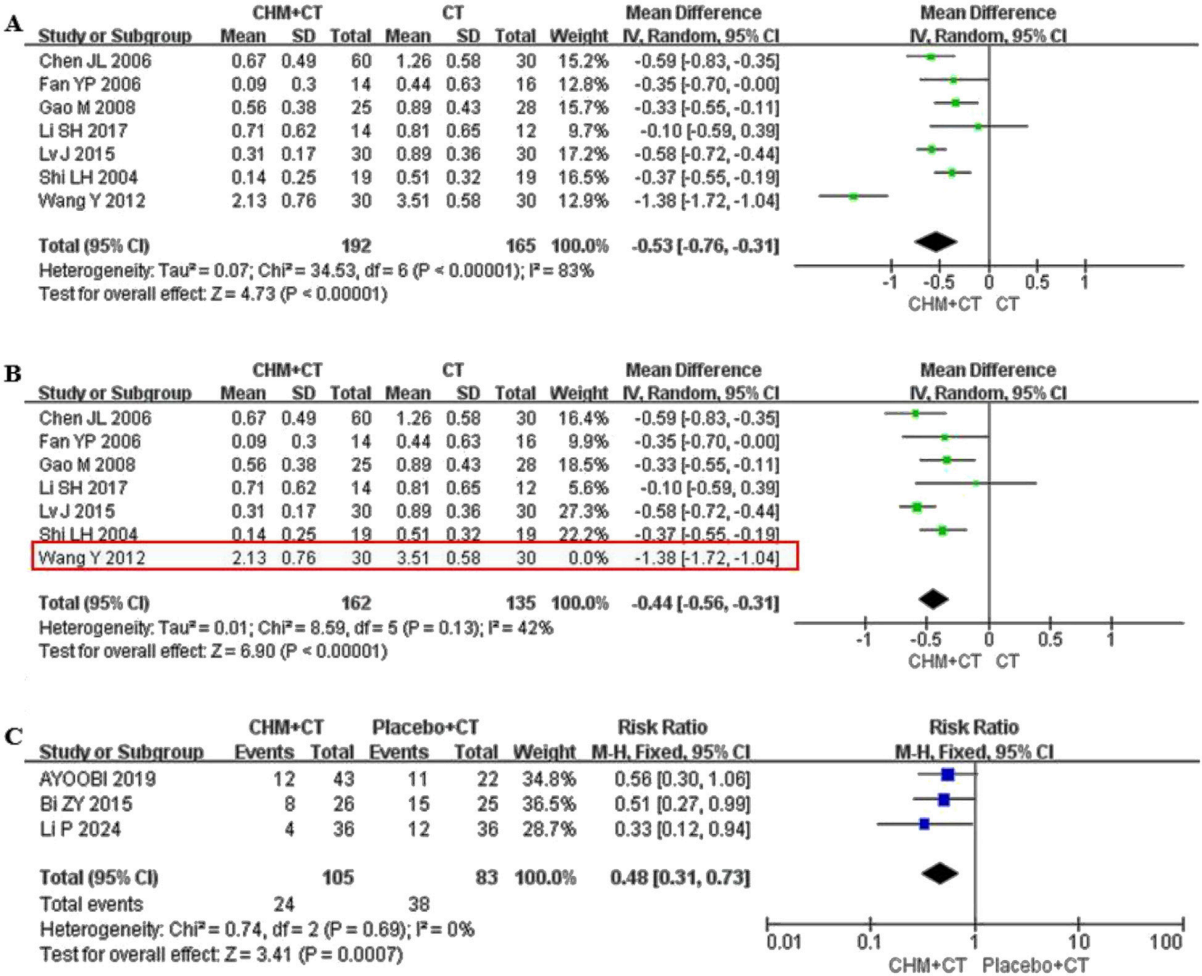
Effect of CHM on annual relapse frequency and ARR. **(A)** CHM + CT versus CT on annual relapse frequency. **(B)** Sensitivity analysis of the annual relapse frequency of CHM combined with CT and CT. **(C)** CHM + CT versus Placebo + CT on ARR.

Eight trials reported on annual relapse frequency (Ayoobi et al., 2019; Chen et al., 2006; Fan et al., 2006; Gao et al., 2008; Li and Yan, 2017; Lv, 2015; Shi and Wang, 2004; Wang et al., 2012). Ayoobi et al. (2019) compared CHM with placebo (MD = −0.811; 95% CI: −1.26, −0.36; *P* < 0.0045), while other seven trials conducted conventional treatment add-on therapy. A random effect model was used due to the high heterogeneity (Cochrane Q test = 34.53, I^2^ = 83%, *P* < 0.00001). The pooled results indicated that adding CHM could decrease annual relapse frequency compared to CT alone (MD = −0.53; 95% CI: −0.76, −0.31; *P* < 0.00001) (Figure 4A). Moreover, sensitivity analysis indicated that after excluding the study by Wang et al. (2012), the heterogeneity significantly decreased (Cochrane Q test = 8.59, I^2^ = 42%, *P* = 0.13) (Figure 4B). Three trials reported on ARR (Ayoobi et al., 2019; Bi, 2016; Li et al., 2024). A fixed effect model was selected due to the low heterogeneity (Cochrane Q test = 0.74, I^2^ = 0%, *P* = 0.69). The pooled results indicated that CHM could significantly decrease ARR compared to placebo (RR = 0.48; 95% CI: 0.31, 0.73; *P* = 0.0007) (Figure 4C).

#### 3.5.3 Neurological signs score

**FIGURE 5:**
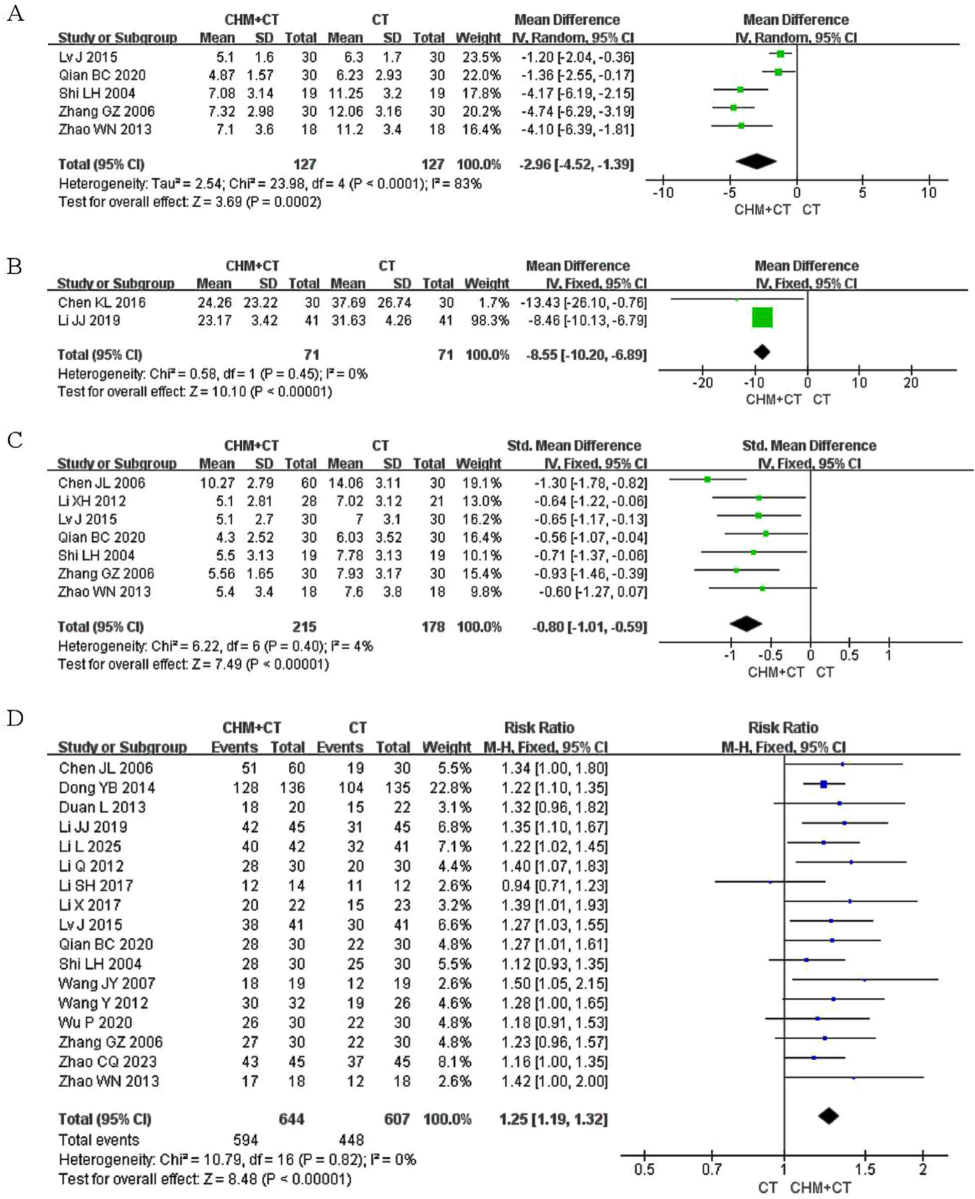
Effect of CHM on neurological signs score, MFIS, clinical symptom scores, and clinical effect. **(A)** CHM + CT versus CT on neurological signs score. **(B)** CHM + CT versus CT on MFIS. **(C)** CHM + CT versus CT on clinical symptom scores. **(D)** CHM + CT versus CT on clinical effect.

The degree of neurological dysfunction was evaluated by the neurological signs score (range: 0–45 points, with higher scores indicating more severe neurological dysfunction). The grading is as follows: mild (0–15), moderate (16–30), and severe (31–45). Eight aspects were evaluated: Consciousness, gaze function, facial paralysis, speech, upper limb muscle strength, lower limb muscle strength, hand muscle strength, and walking ability. Five trials evaluated the neurological signs score (Lv, 2015; Qian and Wang, 2020; Shi and Wang, 2004; Zhao, 2013; Zhang and Zhang, 2006). A random effect model was conducted due to the high heterogeneity (Cochrane Q test = 23.98, I^2^ = 83%, *P* < 0.0001). The pooled results revealed that integrating CHM with CT therapy led to significant improvements in neurological function compared to CT alone (MD = −2.96; 95% CI: −4.52, −1.39; *P* = 0.0002) (Figure 5A).

#### 3.5.4 MFIS

Two trials reported on MFIS (Chen and Fan, 2016; Li et al., 2019). A fixed effect model was conducted due to the low heterogeneity (Cochrane Q test = 0.58, I^2^ = 0%, *P* = 0.45). The pooled results indicated that CHM could significantly relieve fatigue symptoms compared to CT alone (MD = −8.55; 95% CI: −10.20, −6.89; *P* < 0.00001) (Figure 5B).

#### 3.5.5 Clinical symptom scores

Clinical symptoms were rated on a scale of 1–3 points based on their severity, classified as mild, moderate, and severe. Seven trials assessed clinical symptom scores (Chen et al., 2006; Li, 2012; Lv, 2015; Qian and Wang, 2020; Shi and Wang, 2004; Zhao, 2013; Zhang and Zhang, 2006). A fixed effect model was conducted due to the low heterogeneity (Cochrane Q test = 6.22, I^2^ = 4%, *P* = 0.40). The pooled results indicated that combining CHM with CT therapy could significantly improve clinical symptoms compared to CT alone (SMD = −0.80; 95% CI: −1.01, −0.59; *P* < 0.00001) (Figure 5C).

#### 3.5.6 Magnetic resonance imaging

Three trials (Ayoobi et al., 2019; Bi, 2016; Wang et al., 2012) assessed MRI-related outcomes. Ayoobi et al. (2019) identified that the mean volume changes of lesions on T2-weighted scans significantly decreased in the CHM group compared to the placebo group at month 12 (*p* = 0.004). Bi ZY (Bi, 2016) reported that the number and volume of MRI lesions in the CHM group were significantly lower than those in the CT-only group at months 12 and 24. Wang et al. (2012) identified that 21 out of 30 patients in the CHM group exhibited a decrease in the number or scope of lesions on MRI, compared to 16 out of 30 patients in the control group.

#### 3.5.7 Clinical effect

The clinical effect was assessed based on the improvement of symptoms (numbness or weakness in extremities, pain, difficulty walking or imbalance, dysarthria, dysphagia, and bladder dysfunction) and signs (spasticity, ataxia, sensory abnormalities, and cognitive impairment). This evaluation was reported in 17 trials following the intervention. Among these, 17 rials (Chen et al., 2006; Dong, 2014; Duan, 2013; Li, 2017; Li and Yan, 2017; Li and Zhao, 2012; Li et al., 2019; Li et al., 2025; Lv, 2015; Qian and Wang, 2020; Shi and Wang, 2004; Wang et al., 2007; Wang et al., 2012; Wu et al., 2020; Zhao, 2013; Zhao et al., 2023; Zhang and Zhang, 2006) conducted involved CT add-on therapy. A fixed effect model was carried out due to the low heterogeneity (Cochrane Q test = 10.79, I^2^ = 0%, *P* = 0.82). The pooled results indicated that integrated CHM with CT therapy could significantly improve clinical efficacy compared to CT alone (RR = 1.25; 95% CI: 1.19, 1.32; *P* < 0.00001) (Figure 5D). However, one trial (Fan et al., 2018) found non-significant improvement when compared with placebo.

#### 3.5.8 Cognitive function

Three studies assessed cognitive impairment-related outcomes. Ayoobi et al. (2019) found that there was no statistically significant difference between CHM and placebo groups on the Mini-Mental State Examination (MMSE) task. Conversely, Zhao et al. (2023) identified that CHM and CT groups exhibited an increase in Montreal Cognitive Assessment (MoCA) and MMSE scores (*P* < 0.01). Furthermore, the MoCA and MMSE scores in the CHM group were higher than those in the CT alone group (*P* < 0.01). Li et al. (2024) found that the Loewenstein Occupational Therapy Cognitive Assessment-II scores of the CHM group were higher than those of the placebo group at month 12 (*P* = 0.003).

#### 3.5.9 MSFC

Regarding the MSFC, only one study addressed this measure. Ayoobi et al. (2019) found that the MSFC z-score significantly increased in the group with CHM compared to placebo (*p* = 0.003) at month 12.

#### 3.5.10 Scores of TCM syndrome

**FIGURE 6:**
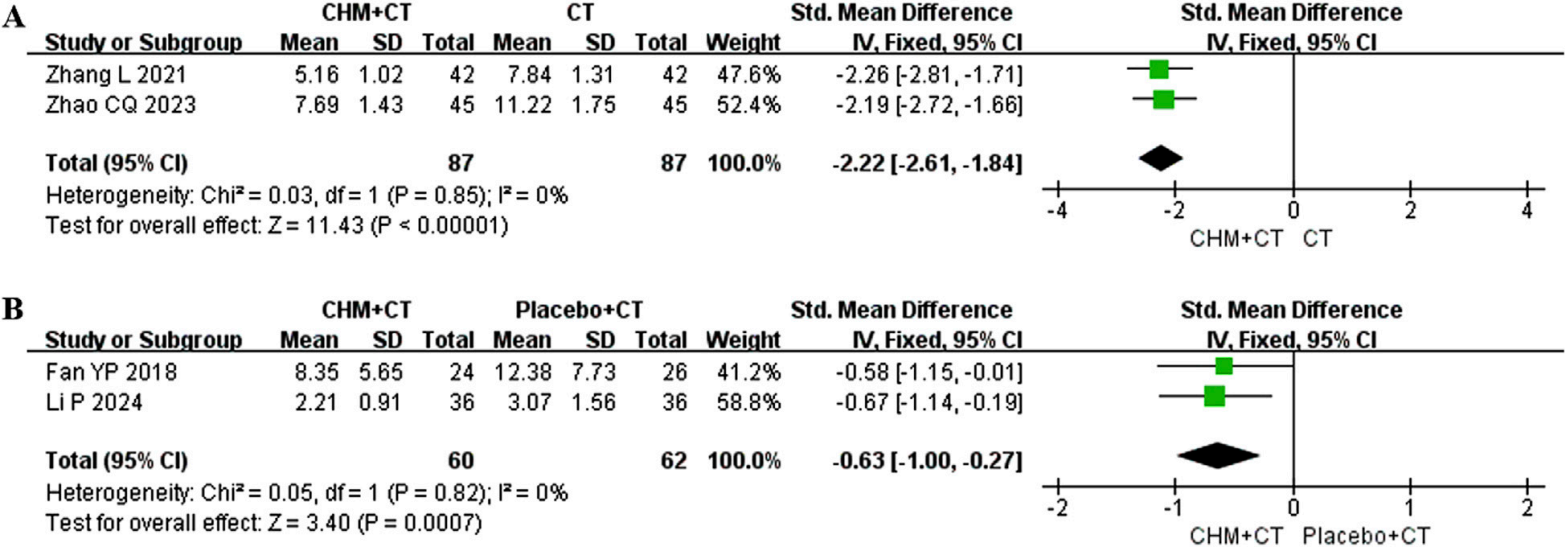
Effect of CHM on scores of TCM syndrome. **(A)** CHM + CT versus CT on scores of TCM syndrome. **(B)** CHM + CT versus placebo + CT on scores of TCM syndrome.

Four trials recorded the changes in symptoms (weakness, numbness, or stiffness in limbs, dizziness, pain, insomnia, fatigue, irritability, constipation, and shortness of breath) and signs (the appearance of tongue body and coating, the position, rate, and strength of pulse) based on TCM theory. Among these, two trials (Zhao et al., 2023; Zhang, 2021) conducted involved the CT add-on therapy. A fixed effect model was used due to the low heterogeneity (Cochrane Q test = 0.03, I^2^ = 0%, *P* = 0.85). The pooled results indicated that adding CHM could improve TCM syndrome compared to CT alone (SMD = −2.22; 95% CI: −2.61, −1.84; *P* < 0.00001) (Figure 6A). The other two trials (Fan et al., 2018; Li et al., 2024) compared CHM to placebo. Similarly, a fixed effect model was used due to the low heterogeneity (Cochrane Q test = 0.05, I^2^ = 0%, *P* = 0.82). The pooled results indicated that CHM could improve TCM syndrome compared to placebo (SMD = −0.63; 95% CI: −1.00, −0.27; *P* = 0.0007) (Figure 6B).

#### 3.5.11 Pharmacodynamic biomarkers

Six trials (Jie, 2024; Li, 2017; Li et al., 2019; Li et al., 2025; Yuan et al., 2025; Zhao et al., 2023) reported significant differences in biofluid markers (*P* < 0.05) in the CHM group when compared with the CT group. These markers included inflammatory factors (interferon-γ↓, tumor necrosis factor-α↓, matrix metalloproteinase-9↓, interleukin-1β↓, interleukin-4↓, interleukin-6↓, interleukin-10↑, interleukin-17↓, interleukin-23↓ high mobility group box-1 protein↓, and interleukin-12p40 ↑), neuro-factors (transforming growth factor-β_1_↑, S-100 protein↓, and Tau protein↓), serum T lymphocyte subsets (Th17↓, Treg↑, Th17/Treg↓, CD8^+^ ↑, and CD4^+^/CD8^+^↓), and cerebrospinal fluid T lymphocyte subsets (CD4^+^↓, CD8^+^↑, and CD4^+^/CD8^+^↓).

#### 3.5.12 Adverse effects

A total of 12 studies evaluated the safety of various treatments (Table 4). Among these, nine studies reported adverse events, while the remaining three reported none (Ayoobi et al., 2019; Fan et al., 2018; Shi and Wang, 2004). Six studies (Li et al., 2019; Li et al., 2025; Wang et al., 2007; Wang et al., 2012; Zhao, 2013; Zhao et al., 2023; Zhang, 2021) reported gastrointestinal reactions (abdominal discomfort, diarrhea, nausea and vomiting), anxiety, insomnia, edema, chest distress, taste disorders, headache, and rash. Two studies (Fan et al., 2006; Lv, 2015) reported hormone related adverse effect, including facial flushing, facial acne, obesity, abnormal laboratory tests (elevated transaminase, elevated urea nitrogen, elevated blood glucose, elevated blood lipids, decreased potassium, decreased blood calcium, elevated blood pressure, elevated white blood cells, and neutrophils). In these two studies, the incidence of elevated WBC or NE in the CHM group was significantly lower than that in the CT group (*P* < 0.05). There was no statistically significant difference in other hormone-related adverse effects between CHM and CT groups.

**TABLE 4:** Adverse effect of included studies.

Study	Treatment (N)	Control (N)	Treatment AEs (N)	Control AEs (N)
Ayoobi et al. (2019)	*Achillea millefolium* (43)	Placebo (22)	None (0)	None (0)
Fan et al. (2006)	*Erhuang formula* + CT (30)	CT (35)	Facial flushing (2), acne (4), insomnia (1),↑WBC/NE (16),↑Transaminase (8), ↑urea nitrogen level (2), ↑blood glucose (8), ↑blood lipids level (3), ↓blood potassium level (3), ↓blood calcium level (2)	Facial flushing (4), acne (2), insomnia (1),↑WBC/NE (27),↑Transaminase (14), ↑urea nitrogen level (3), ↑blood glucose (12), ↑blood lipids level (8), ↓blood potassium level (6), ↓blood calcium level (2)
Fan et al. (2018)	*Bushen Yisui* capsule + CT (24)	Placebo + CT (26)	None (0)	None (0)
Li et al. (2019)	*Yishen Busui Tongluo* decoction + CT (41)	CT (41)	Gastrointestinal discomfort (1), nausea (1), taste disorder (1), anxiety (1)	Gastrointestinal discomfort (2), nausea (1), taste disorder (2), anxiety (2), skin rash (1)
Li et al. (2025)	Si Miao pills + CT (42)	CT (41)	Gastrointestinal discomfort (1), nausea and vomiting (2), headache (2), acne (1)	Nausea and vomiting (1), headache (1), acne (1)
Lv (2105)	*Yishen-Bushui-Tongluo* decoction + CT (30)	CT (30)	Facial flushing (2), acne (3), insomnia (5),↑WBC/NE (9),↑Transaminase (8), ↑urea nitrogen level (2), ↑blood glucose (4), ↑blood lipids level (5), ↓blood potassium level (3), ↓blood calcium level (3)	Facial flushing (4), acne (6), insomnia (8),↑WBC/NE (18),↑Transaminase (9), ↑urea nitrogen level (2), ↑blood glucose (4), ↑blood lipids level (6), ↓blood potassium level (4), ↓blood calcium level (4)
Shi and Wang (2004)	*Jiannao Gusui decoction* + CT (19)	CT (19)	None (0)	None (0)
Wang et al. (2007)	*Jiannao Bushen Gusui* formula + CT (32)	CT (26)	Abdominal pain (1)	Obesity and acne (12)
Wang et al. (2012)	*Dihuang Yinzi* decoction + CT (30)	CT (30)	Gastrointestinal discomfort (1), ↑blood pressure (1), ↑blood glucose (2), insomnia (2)	Gastrointestinal discomfort (4), ↑blood glucose (5), insomnia (4)
Zhang (2021)	*Yisui* granules + CT (42)	CT (42)	Chest distress (2), skin rash (1), diarrhea (1), nausea (1)	Chest distress (1), nausea (2)
Zhao et al. (2023)	*Yishen Jiedu Tongluo* decoction + CT (45)	CT (45)	Vomiting (1), nausea (1), ↑blood pressure (1)	Edema (1), vomiting (1), nausea (2)
Zhao (2013)	*Bushen Gusui* pills + CT (18)	CT (18)	None (0)	Psychiatric symptom (1), acne and obesity (2), liver dysfunction (1)

### 3.6 Publication bias

**FIGURE 7:**
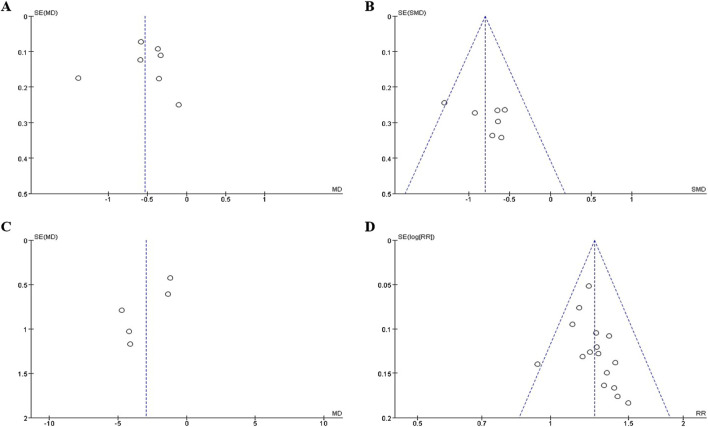
The funnel plot of outcomes: **(A)** annual relapse frequency; **(B)** clinical symptom scores; **(C)** neurological signs score; **(D)** clinical effect.

**FIGURE 8:**
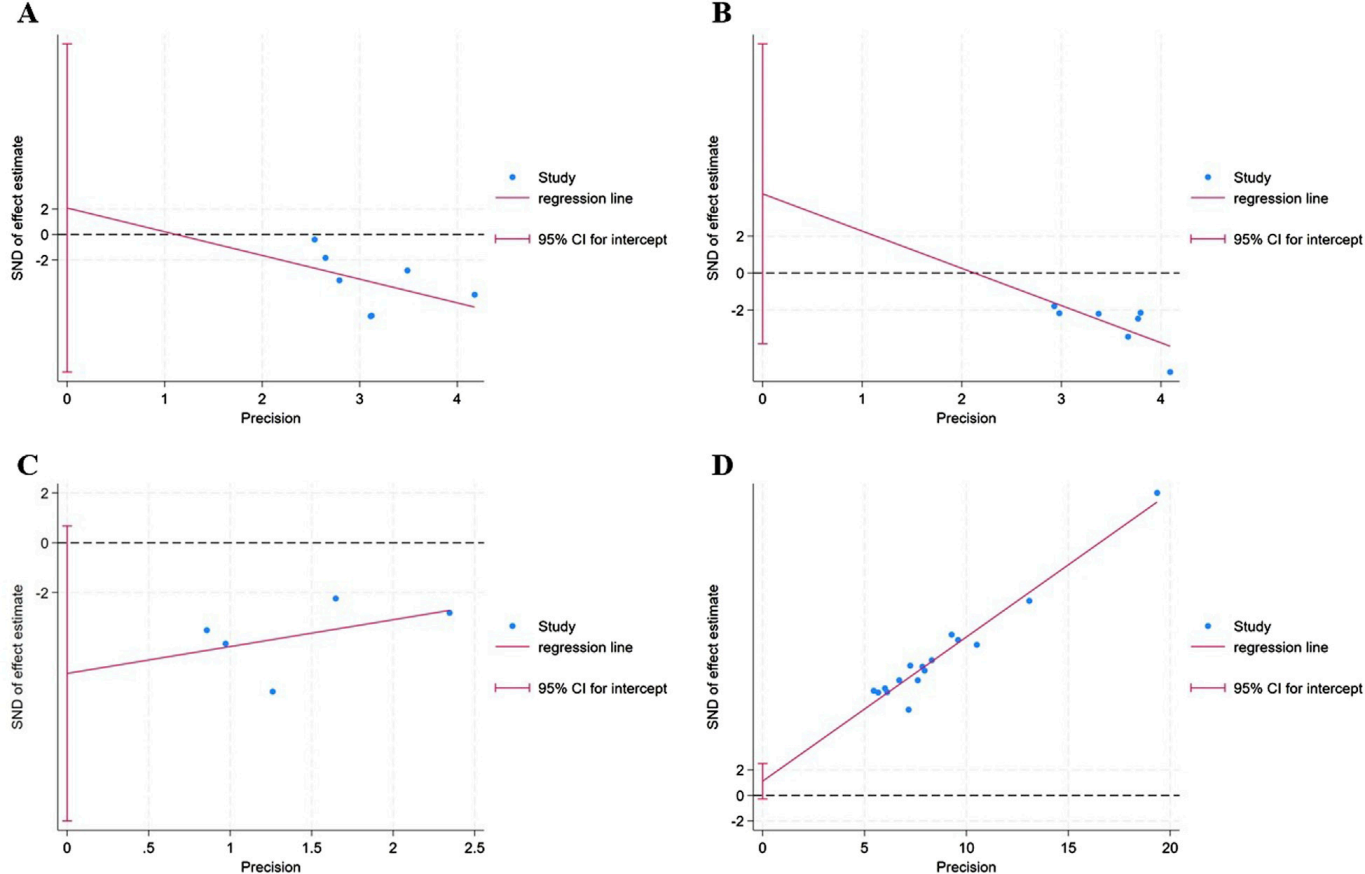
The Egger’s test of outcomes: **(A)** annual relapse frequency; **(B)** clinical symptom scores; **(C)** neurological signs score; **(D)** clinical effect.

The funnel plot (Figures 7A–D) of annual relapse frequency, clinical symptom scores, neurological signs score and clinical effect, indicated a slight publication bias, while no statistically significant publication bias was detected through Egger’s test (*P* = 0.696, 0.232, 0.067, and 0.109, respectively) (Figures 8A–D).

### 3.7 Quality of evidence

The GRADE system was used to assess the quality of evidence for all outcomes (Table 5).

**TABLE 5:** GRADE evidence profiles.

Outcomes	Certainty assessment								No. of patients			Effect		Certainty
	No. of studies	Study design	Risk of bias	Inconsistency	Indirectness	Imprecision	Other considerations		Experimental	Control		Relative (95% CI)	Absolute (95% CI)	
EDSS	4 (CHM + CT vs. Placebo + CT)	Randomised trials	Serious^a^	Not serious	Not serious	Not serious	None		129	110		-	MD 0.65 lower (0.96 lower to 0.35 lower)	⊕⊕⊕○ Moderate
Relapse	7 (annual relapse frequency)	Randomised trials	Serious^a^	Very serious^b^	Not serious	Not serious	None		192	165		-	MD 0.53 lower (0.76 lower to 0.31 lower)	⊕○○○ Very low
	3 (annual relapse rate)	Randomised trials	Serious^a^	Not serious	Not serious	Serious^c^	None		105	83		RR 0.48 (0.31–0.73)	-	⊕⊕○○ Low
Neurological signs score	5	Randomised trials	Serious^a^	Very serious^b^	Not serious	Not serious	None		127	127		-	MD 2.96 lower (4.52 lower to 1.39 lower)	⊕○○○ Very low
Modified fatigue impact scale	2	Randomised trials	Serious^a^	Not serious	Not serious	Serious^c^	None		71	71		-	MD 8.55 lower (10.20 lower to 6.89 lower)	⊕⊕○○ Low
Clinical symptom scores	7	Randomised trials	Serious^a^	Not serious	Not serious	Not serious	None		215	178		-	SMD 0.80 lower (1.01 lower to 0.59 lower)	⊕⊕⊕○ Moderate
Clinical effect	17	Randomised trials	Serious^a^	Not serious	Not serious	Not serious	None		644	607		RR 1.25 (1.19–1.32)	-	⊕⊕⊕○ Moderate
Scores of TCM syndrome	2 (CHM vs. CHM + CT)	Randomised trials	Serious^a^	Not serious	Not serious	Serious^c^	None		87	87		-	SMD 2.22 lower (2.61 lower to 1.84 lower)	⊕⊕○○ Low
	2 (CHM + CT vs. Placebo + CT)	Randomised trials	Serious^a^	Not serious	Not serious	Serious^c^	None		60	62		-	SMD 0.63 lower (1.00 lower to 0.27 lower)	⊕⊕○○ Low

^a^ Hiding or blinding was not used in most studies.

^b^ The heterogeneity test was significant, or I^2^ > 75%.

^c^ Sample size was less than 100 per group.

## 4 Discussion

The systematic review summarized the efficacy and safety of CHM therapy for the treatment of MS. A total of 28 trials, involving 1,971 participants, were included in the analysis. Our findings suggested that CHM therapy may be effective in reducing the EDSS and relapse frequency. Additionally, it appeared to improve clinical symptom scores, TCM syndrome scores, neurological signs, cognitive function, and fatigue levels. Moreover, quite a few studies indicated that the clinical effect of adding CHM therapy was significantly favorable than that of the CT. However, the limited quality of the supporting data constrains the degree of confidence that can be attributed to the results. Additionally, only 12 out of 26 trials reported adverse events. Among these, one trial involving patients in the acute phase of RRMS reported no adverse events despite the use of corticosteroids, which may suggest underreporting rather than a true absence of adverse effects, particularly considering the known side effects of conventional therapies such as corticosteroids. Therefore, enhanced monitoring and standardized reporting of adverse events are necessary to ensure a reliable evaluation of the safety of these interventions.

Through a systematic review of the CHM therapies addressed in the RCTs, we found that the clinical treatment of MS in TCM mainly focuses on nourishing kidney yin and warming kidney yang. Additionally, it removes dampness, dispelling turbidity, and unblocking collaterals. This is consistent with the current mainstream understanding of the pathogenesis, which suggests that MS is caused by insufficient innate endowment and kidney qi deficiency. This condition leads to dysfunction of the organs, accumulation of dampness or dampness heat infiltration, the production of turbid toxins, damage to the Du meridian, brain, and spinal cord (Gao et al., 2022). Currently, experimental evidence has supported that CHM with the above effects can improve neurological deficits, reduce pathological damage such as central nervous system inflammatory cell infiltration and myelin sheath loss. Additionally, it can improve blood-brain barrier permeability in mice with experimental autoimmune encephalomyelitis, a traditional animal model of MS (Wu et al., 2012; Zhu et al., 2016). Moreover, several studies suggest that MS has a prodromal period (Makhani and Tremlett, 2021; Marrie et al., 2022). Managing risk factors during this prodromal phase, before a formal diagnosis of MS, and implementing secondary prevention strategies after a diagnosis of clinically isolated syndrome have gradually become research hotspots. Accordingly, the intervention time of CHM could potentially be initiated earlier, particularly during the stage when turbidity and toxicity are not abundant and healthy qi is not deficient. However, such attempts to treat MS using TCM must be supported by rigorous scientific evaluations in well-designed clinical studies.

Although the present study concluded that CHM demonstrates a good efficacy and safety profile in treating MS based on the results of available RCTs, the poor methodological quality and high clinical heterogeneity of the included trials require special attention. It is well-established that there are significant differences in pathological mechanisms, disease progression, and clinical manifestations among different clinical types of MS. When conducting clinical research, stratified analysis is usually necessary to evaluate the treatment efficacy more accurately, avoid bias and confounding, and increase the accuracy and reliability of the results. More precise and restrictive inclusion criteria for clinical trials should be established, as some RCTs included in this study did not adequately define the clinical characteristics necessary for participation, such as disease type, duration, and neurological function of patients with MS. Recruiting homogeneous subjects is necessary, particularly in clinical trials with a small sample size. Alternatively, the concept of treatment with syndrome differentiation guides the process of TCM diagnosis and treatment. The diagnostic criteria for syndrome differentiation play a crucial role in guiding medication choices. Consequently, a reliable TCM syndrome diagnosis and evaluation scale for MS is needed in the future to ensure consistency and reproducibility in research. Several included studies did not report the source or concentration of CHM preparations, contributing to increased heterogeneity of interventions. The uncertainty regarding herbal constituents and manufacturing processes may compromise the objective evaluation of CHM efficacy and reduce the credibility and reproducibility of the findings.

Regarding the selection of outcome measures, some included studies did not evaluate MRI and relapse rate. Currently, MS is mainly monitored and evaluated regularly through clinical, imaging, and biomarker dimensions to achieve no evidence of disease activity (NEDA) (Mayssam et al., 2020). This includes evaluating clinical recurrence (assessed by ARR), disability deterioration (assessed by EDSS), active lesions (evaluated through MRI), and biomarkers such as neurofilament light chain. We recommend incorporating NEDA as an efficacy indicator in future research on CHM. When evaluating the efficacy of CHM for MS, common measures include clinical symptom scores, TCM syndrome scores, and clinical effects. However, these assessments are subjective and influenced by evaluator experience and inconsistent standards, limiting comparability and reliability. Therefore, it is vital to establish standardized, objective criteria and improve evaluator training to enhance the accuracy and consistency of efficacy evaluations, thereby strengthening the foundation for clinical research and practice.

This systematic review has several limitations. First, the methodological quality of the included studies was assessed as low according to the Cochrane Handbook. The risk of bias assessment revealed that essential components of trial design—such as random sequence generation, allocation concealment, and blinding of participants and assessors—were either inadequately described or absent in a significant proportion of the studies. These deficiencies raise serious concerns about the internal validity of the reported outcomes. Particularly, the lack of blinding may introduce performance and detection biases, specifically in trials that rely heavily on subjective outcome measures. Second, the diagnostic criteria for some of the included studies were not specified, which may lead to bias. Third, the clinical types of MS population, the disability status of participants, the course of treatment, compositions of herbal formula, and dosage of CHM therapy varied among the included studies. This variability could further introduce bias. Moreover, incomplete reporting of relevant data further limited the feasibility of subgroup analyses and hindered comprehensive evidence synthesis. Fourth, the small sample size of included studies affected the pooled results. Lastly, although we did not restrict the language of the published literature, most included studies were from China, which limits the applicability of results to populations with different genetic and environmental risk factors for MS. Consequently, further rigorous RCTs that are well-designed, multi-center, and feature appropriate participant selection, long-term follow-up, and effective outcome measures are still required.

## 5 Conclusion

This systematic review and meta-analysis provide an updated and comprehensive evaluation of the clinical efficacy of CHM interventions for MS. The findings suggest that CHM therapy may play a role in alleviating the disability status and clinical symptoms, while also reducing relapse frequency with a low incidence of adverse effects. However, further well-designed RCTs with high-quality, appropriate participants and efficient outcome measures are warranted to strengthen clinical evidence of the efficacy and safety of CHM on MS management.

## Data Availability

The original contributions presented in the study are included in the article/Supplementary Material, further inquiries can be directed to the corresponding authors.

## Abbreviations

ARR, Annualized relapse rate; CAM, Complementary and alternative medicine; CHM, Chinese herbal medicine; CI, Confidence intervals; CNKI, The Chinese National Knowledge Infrastructure; CT, Conventional therapy; DMTs, Disease-modifying therapies; EDSS, Expanded disability status scale; GRADE, The Grading of Recommendations, Assessment, Development and Evaluation system; HMGB1, High mobility group box-1 protein; IFN-γ, Interferon-γ; IL-10, Interleukin-10; IL-12p40, Interleukin-12p40; IL-6, Interleukin-6; LOTCA-II, Loewenstein Occupational Therapy Cognitive Assessment-II; MD, Mean difference; MFIS, Modified Fatigue Impact Scale; MMP-9, Matrix metalloproteinase-9; MMSE, Mini-Mental State Examination; MoCA, Montreal Cognitive Assessment; MRI, Magnetic resonance imaging; MS, Multiple sclerosis; MSFC, Multiple sclerosis functional composite; NE, Neutrophil; NEDA, No evidence of disease activity; PPMS, Primary progressive MS; PRISMA, Preferred Reporting Items for Systematic reviews and Meta-Analyses; RCT, Randomized controlled trials; RR, Relative risk; RRMS, Relapsing remitting multiple sclerosis; SinoMed, The Chinese Biomedical Database; SMD, Standard mean difference; SPMS, Secondary progressive multiple sclerosis; TCM, Traditional Chinese Medicine; TGF-β_1_, Transforming growth factor-β_1_; VIP, The Chinese Science and Technology Journals Database; WBC, White blood cell.
