# Supplementary material for: Efficacy and safety of Chinese herbal medicine for multiple sclerosis: a systematic review and meta-analysis of randomized controlled trials
Source: Front Pharmacol. 2025 Sep 22;16:1635833. doi: 10.3389/fphar.2025.1635833 (PMC12497746; doi:10.3389/fphar.2025.1635833)
Supplement: Supplementary file 1 [file Supplementaryfile1.docx]

***Supplementary Material***

# Appendix 1. Search strategies

# Appendix 2.The chemical and botanical characterization of the involved preparation according to the “Four Pillars of Best Practice”.

# Appendix 1. Search strategies

## <inception to the October 2024>

**Pubmed 568**

((((((((((((((((((((((((((Essences, Flower[Title/Abstract]) OR (Bach Rescue Remedy[Title/Abstract])) OR (Remedy, Bach Rescue[Title/Abstract])) OR (Rescue Remedy, Bach[Title/Abstract])) OR (Bach Flower Remedies[Title/Abstract])) OR (Flower Remedies, Bach[Title/Abstract])) OR (Remedies, Bach Flower[Title/Abstract])) OR (Bach Flowers[Title/Abstract])) OR (Flowers, Bach[Title/Abstract])) OR (Bach Flower Essences[Title/Abstract])) OR (Essences, Bach Flower[Title/Abstract])) OR (Flower Essences, Bach[Title/Abstract])) OR (Aromatherapies[Title/Abstract])) OR (Aroma Therapy[Title/Abstract])) OR (Aroma Therapies[Title/Abstract])) OR (Therapies, Aroma[Title/Abstract])) OR (Therapy, Aroma[Title/Abstract])) OR (Volatile Oils[Title/Abstract])) OR (Oil, Essential[Title/Abstract])) OR (Essential Oil[Title/Abstract])) OR (Oils, Essential[Title/Abstract])) OR (Essential Oils[Title/Abstract])) OR (Volatile Oil[Title/Abstract])) OR (Oil, Volatile[Title/Abstract])) OR ((("Flower Essences"[Mesh]) OR "Aromatherapy"[Mesh]) OR "Oils, Volatile"[Mesh])) OR (((((((((("Plants, Medicinal"[Mesh]) OR "Phytotherapy"[Mesh]) OR "Drugs, Chinese Herbal"[Mesh]) OR "Medicine, Traditional"[Mesh]) OR "Herbal Medicine"[Mesh]) OR "Plant Extracts"[Mesh]) OR "Plant Preparations"[Mesh]) OR "Medicine, Chinese Traditional"[Mesh]) OR "Medicine, Tibetan Traditional"[Mesh]) OR ((((((((((((((((((((((((((((((((((((((((((((((((((((((((((((((Medicinal Plant[Title/Abstract]) OR (Plant, Medicinal[Title/Abstract])) OR (Medicinal Plants[Title/Abstract])) OR (Medicinal Herbs[Title/Abstract])) OR (Herb, Medicinal[Title/Abstract])) OR (Medicinal Herb[Title/Abstract])) OR (Herbs, Medicinal[Title/Abstract])) OR (Pharmaceutical Plants[Title/Abstract])) OR (Pharmaceutical Plant[Title/Abstract])) OR (Plant, Pharmaceutical[Title/Abstract])) OR (Plants, Pharmaceutical[Title/Abstract])) OR (Healing Plants[Title/Abstract])) OR (Healing Plant[Title/Abstract])) OR (Plant, Healing[Title/Abstract])) OR (Plants, Healing[Title/Abstract])) OR (Herbal Therapy[Title/Abstract])) OR (Herb Therapy[Title/Abstract])) OR (Chinese Drugs, Plant[Title/Abstract])) OR (Chinese Herbal Drugs[Title/Abstract])) OR (Herbal Drugs, Chinese[Title/Abstract])) OR (Plant Extracts, Chinese[Title/Abstract])) OR (Chinese Plant Extracts[Title/Abstract])) OR (Extracts, Chinese Plant[Title/Abstract])) OR (Remedy, Home[Title/Abstract])) OR (Medicine, Primitive[Title/Abstract])) OR (Primitive Medicine[Title/Abstract])) OR (Medicine, Folk[Title/Abstract])) OR (Folk Medicine[Title/Abstract])) OR (Medicine, Indigenous[Title/Abstract])) OR (Indigenous Medicine[Title/Abstract])) OR (Folk Remedies[Title/Abstract])) OR (Folk Remedy[Title/Abstract])) OR (Remedies, Folk[Title/Abstract])) OR (Remedy, Folk[Title/Abstract])) OR (Ethnomedicine[Title/Abstract])) OR (Medicine, Herbal[Title/Abstract])) OR (Hawaiian Herbal Medicine[Title/Abstract])) OR (Herbal Medicine, Hawaiian[Title/Abstract])) OR (Medicine, Hawaiian Herbal[Title/Abstract])) OR (Herbalism[Title/Abstract])) OR (Extracts, Plant[Title/Abstract])) OR (Plant Extract[Title/Abstract])) OR (Extract, Plant[Title/Abstract])) OR (Herbal Preparation[Title/Abstract])) OR (Plant Preparation[Title/Abstract])) OR (Preparation, Plant[Title/Abstract])) OR (Traditional Chinese Medicine[Title/Abstract])) OR (Chung I Hsueh[Title/Abstract])) OR (Traditional Medicine, Chinese[Title/Abstract])) OR (Zhong Yi Xue[Title/Abstract])) OR (Chinese Traditional Medicine[Title/Abstract])) OR (Chinese Medicine, Traditional[Title/Abstract])) OR (Traditional Tongue Diagnosis[Title/Abstract])) OR (Tongue Diagnos*[Title/Abstract])) OR (Tongue Assessment, Traditional[Title/Abstract])) OR (Tibetan Traditional Medicine[Title/Abstract])) OR (Tibetan Medicine, Traditional[Title/Abstract])) OR (Medicine, Traditional Tibetan[Title/Abstract])) OR (Traditional Tibetan Medicine[Title/Abstract])) OR (Tibetan Medicine[Title/Abstract])) OR (Medicine, Tibetan[Title/Abstract])) OR (Traditional Medicine, Tibetan[Title/Abstract])))) AND (((((((Multiple sclerosis[Title/Abstract]) OR (Sclerosis, Multiple[Title/Abstract])) OR (Sclerosis, Disseminated[Title/Abstract])) OR (Disseminated Sclerosis[Title/Abstract])) OR (MS (Multiple Sclerosis[Title/Abstract]))) OR (Multiple Sclerosis, Acute Fulminating[Title/Abstract])) OR ("Multiple Sclerosis"[MeSH Terms]))

**Web of science 532**

(AB=(Essences, Flower OR Bach Rescue Remedy OR Remedy, Bach Rescue OR Rescue Remedy, Bach OR Bach Flower Remedies OR Flower Remedies, Bach OR Remedies, Bach Flower OR Bach Flowers OR Flowers, Bach OR Bach Flower Essences OR Essences, Bach Flower OR Flower Essences, Bach OR Aromatherapies OR Aroma Therapy OR Aroma Therapies OR Therapies, Aroma OR Therapy, Aroma OR Volatile Oils OR Oil, Essential OR Essential Oil OR Oils, Essential OR Essential Oils OR Volatile Oil OR Oil, Volatile OR Medicinal Plant OR Plant, Medicinal OR Medicinal Plants OR Medicinal Herbs OR Herb, Medicinal OR Medicinal Herb OR Herbs, Medicinal OR Pharmaceutical Plants OR Pharmaceutical Plant OR Plant, Pharmaceutical OR Plants, Pharmaceutical OR Healing Plants OR Healing Plant OR Plant, Healing OR Plants, Healing OR Herbal Therapy OR Herb Therapy OR Chinese Drugs, Plant OR Chinese Herbal Drugs OR Herbal Drugs, Chinese OR Plant Extracts, Chinese OR Chinese Plant Extracts OR Extracts, Chinese Plant OR Remedy, Home OR Medicine, Primitive OR Primitive Medicine OR Medicine, Folk OR Folk Medicine OR Medicine, Indigenous OR Indigenous Medicine OR Folk Remedies OR Folk Remedy OR Remedies, Folk OR Remedy, Folk OR Ethnomedicine OR Medicine, Herbal OR Hawaiian Herbal Medicine OR Herbal Medicine, Hawaiian OR Medicine, Hawaiian Herbal OR Herbalism OR Extracts, Plant OR Plant Extract OR Extract, Plant OR Herbal Preparation OR Plant Preparation OR Preparation, Plant OR Traditional Chinese Medicine OR Chung I Hsueh OR Traditional Medicine, Chinese OR Zhong Yi Xue OR Chinese Traditional Medicine OR Chinese Medicine, Traditional OR Traditional Tongue Diagnosis OR Tongue Diagnosis OR Tongue Assessment, Traditional OR Tibetan Traditional Medicine OR Tibetan Medicine, Traditional OR Medicine, Traditional Tibetan OR Traditional Tibetan Medicine OR Tibetan Medicine OR Medicine, Tibetan OR Traditional Medicine, Tibetan) OR TS=(Flower Essences OR Aromatherapy OR Oils, Volatile OR Plants, Medicinal OR Phytotherapy OR Drugs, Chinese Herbal OR Medicine, Traditional OR Herbal Medicine OR Plant Extracts OR Plant Preparations OR Medicine, Chinese Traditional OR Medicine, Tibetan Traditional)) AND (AB=(Multiple sclerosis OR Sclerosis, Multiple OR Sclerosis, Disseminated OR Disseminated Sclerosis OR MS Multiple Sclerosis OR Multiple Sclerosis, Acute Fulminating) OR TS=(Multiple Sclerosis))

**EMBASE 393**

| No. | Query | Results |
| --- | --- | --- |
| #8 | #6 AND #7 | 393 |
| #7 | #4 OR #5 | 180514 |
| #6 | 'essences, flower':ab,ti OR 'bach rescue remedy':ab,ti OR 'remedy, bach rescue':ab,ti OR 'rescue remedy, bach':ab,ti OR 'bach flower remedies':ab,ti OR 'flower remedies, bach':ab,ti OR 'remedies, bach flower':ab,ti OR 'bach flowers':ab,ti OR 'flowers, bach':ab,ti OR 'bach flower essences':ab,ti OR 'essences, bach flower':ab,ti OR 'flower essences, bach':ab,ti OR aromatherapies:ab,ti OR 'aroma therapy':ab,ti OR 'aroma therapies':ab,ti OR 'therapies, aroma':ab,ti OR 'therapy, aroma':ab,ti OR 'volatile oils':ab,ti OR 'oil, essential':ab,ti OR 'essential oil':ab,ti OR 'oils, essential':ab,ti OR 'essential oils':ab,ti OR 'volatile oil':ab,ti OR 'oil, volatile':ab,ti OR 'medicinal plant':ab,ti OR 'plant, medicinal':ab,ti OR 'medicinal plants':ab,ti OR 'medicinal herbs':ab,ti OR 'herb, medicinal':ab,ti OR 'medicinal herb':ab,ti OR 'herbs, medicinal':ab,ti OR 'pharmaceutical plants':ab,ti OR 'pharmaceutical plant':ab,ti OR 'plant, pharmaceutical':ab,ti OR 'plants, pharmaceutical':ab,ti OR 'healing plants':ab,ti OR 'healing plant':ab,ti OR 'plant, healing':ab,ti OR 'plants, healing':ab,ti OR 'herbal therapy':ab,ti OR 'herb therapy':ab,ti OR 'chinese drugs, plant':ab,ti OR 'chinese herbal drugs':ab,ti OR 'herbal drugs, chinese':ab,ti OR 'plant extracts, chinese':ab,ti OR 'chinese plant extracts':ab,ti OR 'extracts, chinese plant':ab,ti OR 'remedy, home':ab,ti OR 'medicine, primitive':ab,ti OR 'primitive medicine':ab,ti OR 'medicine, folk':ab,ti OR 'folk medicine':ab,ti OR 'medicine, indigenous':ab,ti OR 'indigenous medicine':ab,ti OR 'folk remedies':ab,ti OR 'folk remedy':ab,ti OR 'remedies, folk':ab,ti OR 'remedy, folk':ab,ti OR ethnomedicine:ab,ti OR 'medicine, herbal':ab,ti OR 'hawaiian herbal medicine':ab,ti OR 'herbal medicine, hawaiian':ab,ti OR 'medicine, hawaiian herbal':ab,ti OR herbalism:ab,ti OR 'extracts, plant':ab,ti OR 'plant extract':ab,ti OR 'extract, plant':ab,ti OR 'herbal preparation':ab,ti OR 'plant preparation':ab,ti OR 'preparation, plant':ab,ti OR 'traditional chinese medicine':ab,ti OR 'chung i hsueh':ab,ti OR 'traditional medicine, chinese':ab,ti OR 'zhong yi xue':ab,ti OR 'chinese traditional medicine':ab,ti OR 'chinese medicine, traditional':ab,ti OR 'traditional tongue diagnosis':ab,ti OR 'tongue diagnosis':ab,ti OR 'tongue assessment, traditional':ab,ti OR 'tibetan traditional medicine':ab,ti OR 'tibetan medicine, traditional':ab,ti OR 'medicine, traditional tibetan':ab,ti OR 'traditional tibetan medicine':ab,ti OR 'tibetan medicine':ab,ti OR 'medicine, tibetan':ab,ti OR 'traditional medicine, tibetan':ab,ti OR 'flower essences':ab,ti OR aromatherapy:ab,ti OR 'oils, volatile':ab,ti OR 'plants, medicinal':ab,ti OR phytotherapy:ab,ti OR 'drugs, chinese herbal':ab,ti OR 'medicine, traditional':ab,ti OR 'herbal medicine':ab,ti OR 'plant extracts':ab,ti OR 'plant preparations':ab,ti OR 'medicine, chinese traditional':ab,ti OR 'medicine, tibetan traditional':ab,ti | 178056 |
| #5 | ('chariot disease':ab,ti OR 'disseminated sclerosis':ab,ti OR 'insular sclerosis':ab,ti OR ms:ab,ti) AND 'multiple sclerosis':ab,ti OR 'sclerosis multiplex':ab,ti OR 'sclerosis, disseminated':ab,ti OR 'sclerosis, insular':ab,ti OR 'sclerosis, multiple':ab,ti OR 'multiple sclerosis':ab,ti | 146849 |
| #4 | 'multiple sclerosis'/exp | 166189 |

**The Cochrane Central Register of Controlled Trials (CENTRAL) in the Cochrane Library 74**

ID Search Hits

#1 (multiple sclerosis):ti,ab,kw (Word variations have been searched) 13519

#2 MeSH descriptor: [Multiple Sclerosis] explode all trees 5371

#3 #1 or #2 13519

#4 MeSH descriptor: [Phytotherapy] explode all trees 5108

#5 MeSH descriptor: [Medicine, Traditional] explode all trees 2328

#6 MeSH descriptor: [Plants, Medicinal] explode all trees 1152

#7 MeSH descriptor: [Herbal Medicine] explode all trees 100

#8 MeSH descriptor: [Plant Preparations] explode all trees 15765

#9 MeSH descriptor: [Plant Extracts] explode all trees 11324

#10 MeSH descriptor: [Drugs, Chinese Herbal] explode all trees 4745

#11 #4 or #5 or #6 or #7 or #8 or #9 or #10 18478

#12 MeSH descriptor: [Medicine, Chinese Traditional] explode all trees 1829

#13 MeSH descriptor: [Plants, Medicinal] explode all trees 1152

#14 #11 or #12 or #13 18478

#15 (Sclerosis, Disseminated):ti,ab,kw (Word variations have been searched) 161

#16 (Sclerosis, Multiple):ti,ab,kw (Word variations have been searched) 13519

#17 (Disseminated Sclerosis):ti,ab,kw (Word variations have been searched) 161

#18 (Multiple Sclerosis, Acute Fulminating):ti,ab,kw (Word variations have been searched) 0

#19 #3 or #15 or #16 or #17 or #18 13554

#20 #19 and #14 74

**The VIP information resource integration service platform (cqvip) 295**

M=多发性硬化 AND M=(植物 or 草药 or 中草药 or 中药 or 中医药 or 中草药提取物 or 中药提取物 or 中成药 or 复方 or 汤 or 丸 or 方剂 or 民间医学 or 民间治疗 or 家庭治疗 or 植物提取物 or 植物制剂 or 中国医学 or 传统医学 or 中医 or 西藏 or 藏药 or 花精华素 or 精油 or 香薰 or 芳香疗法)

**SinoMed 559**

(("汤"[常用字段] OR "丸"[常用字段] OR ("方剂"[常用字段] OR "汤头"[常用字段] OR "方剂"[主题词]) OR ("民间医学"[常用字段] OR "传统医学"[常用字段] OR "民族医学"[常用字段] OR "民间治疗"[常用字段] OR "家庭治疗"[常用字段] OR "本土医学"[常用字段] OR "原始医学"[常用字段] OR "医学, 传统"[主题词]) OR ("民间治疗"[常用字段] OR "传统医学"[常用字段] OR "民族医学"[常用字段] OR "民间医学"[常用字段] OR "家庭治疗"[常用字段] OR "本土医学"[常用字段] OR "原始医学"[常用字段] OR "医学, 传统"[主题词]) OR ("家庭治疗"[常用字段] OR "传统医学"[常用字段] OR "民族医学"[常用字段] OR "民间医学"[常用字段] OR "民间治疗"[常用字段] OR "本土医学"[常用字段] OR "原始医学"[常用字段] OR "医学, 传统"[主题词]) OR ("植物提取物"[常用字段] OR "植物提取物"[主题词]) OR ("植物制剂"[常用字段] OR "中草药制剂"[常用字段] OR "植物制剂"[主题词])) OR (("植物"[常用字段] OR "植物"[主题词]) OR ("草药"[常用字段] OR "药用植物"[常用字段] OR "医用草药"[常用字段] OR "植物, 药用"[主题词]) OR ("中草药"[常用字段] OR "中药"[常用字段] OR "中草药提取物"[常用字段] OR "中草药"[主题词]) OR ("中药"[常用字段] OR "中草药"[常用字段] OR "中草药提取物"[常用字段] OR "中草药"[主题词]) OR "中医药"[常用字段] OR ("中草药提取物"[常用字段] OR "中草药"[常用字段] OR "中药"[常用字段] OR "中草药"[主题词]) OR "中药提取物"[常用字段] OR ("中成药"[常用字段] OR "中成药"[主题词]) OR ("复方"[常用字段] OR "复方"[主题词])) OR (("芳香疗法"[常用字段] OR "芳香疗法"[主题词]) OR "香薰"[常用字段] OR "精油"[常用字段] OR ("花精华素"[常用字段] OR "巴哈花精疗法"[常用字段] OR "花精华素"[主题词]) OR ("藏药"[常用字段] OR "藏药"[主题词]) OR "西藏"[常用字段] OR "中医"[常用字段] OR "传统医学"[常用字段] OR "中国医学"[常用字段])) AND ("多发性硬化"[常用字段] OR "MS(多发性硬化)"[常用字段] OR "急性暴发性多发性硬化"[常用字段] OR "弥漫性硬化"[常用字段] OR "多发性硬化"[主题词])

**Chinese National Knowledge Infrastructure Database (CNKI): 337**

**
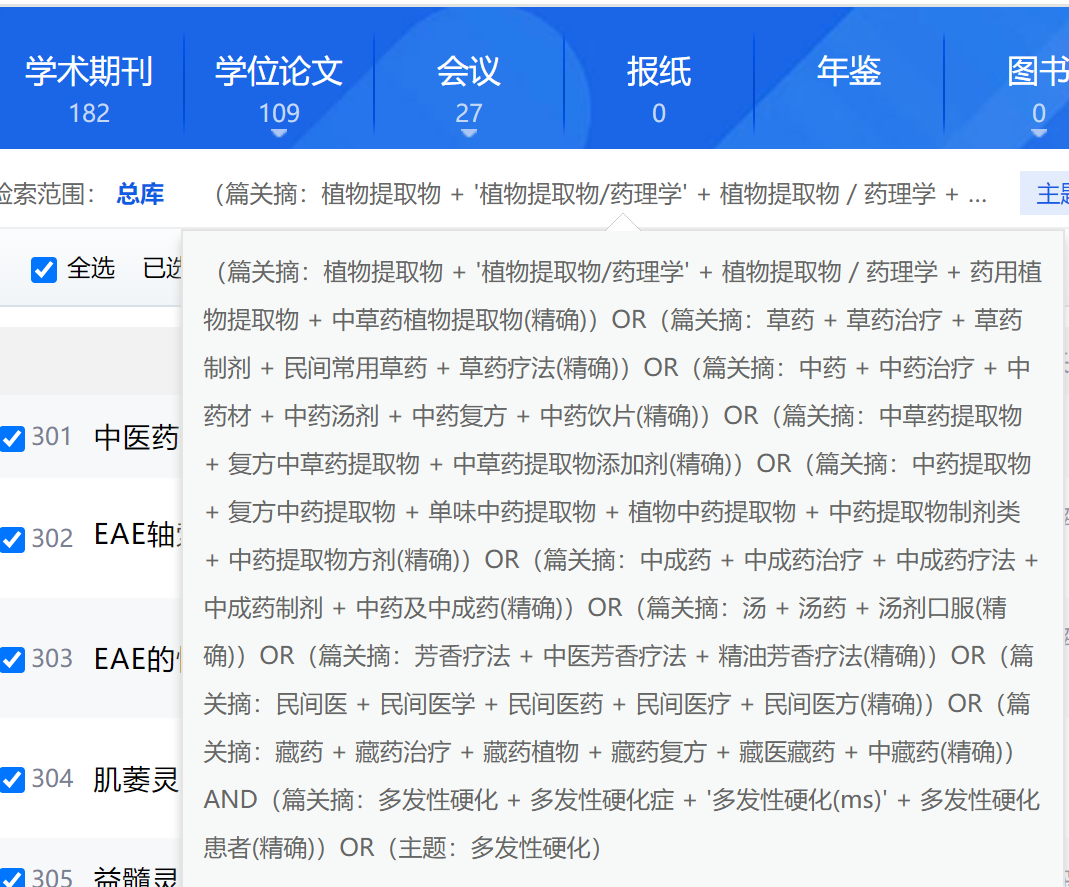
**

**Wanfang data 656**

(主题:(多发性硬化) or 题名或关键词:(多发性硬化)) and (主题:(中医 or 中药 or 草药 or 植物) or 题名或关键词:(植物 or 草药 or 中草药 or 中药 or 中医药 or 中草药提取物 or 中药提取物 or 中成药 or 复方 or 汤 or 丸 or 方剂 or 民间医学 or 民间治疗 or 家庭治疗 or 植物提取物 or 植物制剂 or 中国医学 or 传统医学 or 中医 or 西藏 or 藏药 or 花精华素 or 精油 or 香薰 or 芳香疗法))

## <October 2024 to July 2025>

An initial systematic search was conducted up to October 2024. To enhance the currency of the review, the search was updated in July 2025, and newly identified studies were screened and incorporated accordingly.

**Pubmed: 27**

((((((((((((((((((((((((((Essences, Flower[Title/Abstract]) OR (Bach Rescue Remedy[Title/Abstract])) OR (Remedy, Bach Rescue[Title/Abstract])) OR (Rescue Remedy, Bach[Title/Abstract])) OR (Bach Flower Remedies[Title/Abstract])) OR (Flower Remedies, Bach[Title/Abstract])) OR (Remedies, Bach Flower[Title/Abstract])) OR (Bach Flowers[Title/Abstract])) OR (Flowers, Bach[Title/Abstract])) OR (Bach Flower Essences[Title/Abstract])) OR (Essences, Bach Flower[Title/Abstract])) OR (Flower Essences, Bach[Title/Abstract])) OR (Aromatherapies[Title/Abstract])) OR (Aroma Therapy[Title/Abstract])) OR (Aroma Therapies[Title/Abstract])) OR (Therapies, Aroma[Title/Abstract])) OR (Therapy, Aroma[Title/Abstract])) OR (Volatile Oils[Title/Abstract])) OR (Oil, Essential[Title/Abstract])) OR (Essential Oil[Title/Abstract])) OR (Oils, Essential[Title/Abstract])) OR (Essential Oils[Title/Abstract])) OR (Volatile Oil[Title/Abstract])) OR (Oil, Volatile[Title/Abstract])) OR ((("Flower Essences"[Mesh]) OR "Aromatherapy"[Mesh]) OR "Oils, Volatile"[Mesh])) OR (((((((((("Plants, Medicinal"[Mesh]) OR "Phytotherapy"[Mesh]) OR "Drugs, Chinese Herbal"[Mesh]) OR "Medicine, Traditional"[Mesh]) OR "Herbal Medicine"[Mesh]) OR "Plant Extracts"[Mesh]) OR "Plant Preparations"[Mesh]) OR "Medicine, Chinese Traditional"[Mesh]) OR "Medicine, Tibetan Traditional"[Mesh]) OR ((((((((((((((((((((((((((((((((((((((((((((((((((((((((((((((Medicinal Plant[Title/Abstract]) OR (Plant, Medicinal[Title/Abstract])) OR (Medicinal Plants[Title/Abstract])) OR (Medicinal Herbs[Title/Abstract])) OR (Herb, Medicinal[Title/Abstract])) OR (Medicinal Herb[Title/Abstract])) OR (Herbs, Medicinal[Title/Abstract])) OR (Pharmaceutical Plants[Title/Abstract])) OR (Pharmaceutical Plant[Title/Abstract])) OR (Plant, Pharmaceutical[Title/Abstract])) OR (Plants, Pharmaceutical[Title/Abstract])) OR (Healing Plants[Title/Abstract])) OR (Healing Plant[Title/Abstract])) OR (Plant, Healing[Title/Abstract])) OR (Plants, Healing[Title/Abstract])) OR (Herbal Therapy[Title/Abstract])) OR (Herb Therapy[Title/Abstract])) OR (Chinese Drugs, Plant[Title/Abstract])) OR (Chinese Herbal Drugs[Title/Abstract])) OR (Herbal Drugs, Chinese[Title/Abstract])) OR (Plant Extracts, Chinese[Title/Abstract])) OR (Chinese Plant Extracts[Title/Abstract])) OR (Extracts, Chinese Plant[Title/Abstract])) OR (Remedy, Home[Title/Abstract])) OR (Medicine, Primitive[Title/Abstract])) OR (Primitive Medicine[Title/Abstract])) OR (Medicine, Folk[Title/Abstract])) OR (Folk Medicine[Title/Abstract])) OR (Medicine, Indigenous[Title/Abstract])) OR (Indigenous Medicine[Title/Abstract])) OR (Folk Remedies[Title/Abstract])) OR (Folk Remedy[Title/Abstract])) OR (Remedies, Folk[Title/Abstract])) OR (Remedy, Folk[Title/Abstract])) OR (Ethnomedicine[Title/Abstract])) OR (Medicine, Herbal[Title/Abstract])) OR (Hawaiian Herbal Medicine[Title/Abstract])) OR (Herbal Medicine, Hawaiian[Title/Abstract])) OR (Medicine, Hawaiian Herbal[Title/Abstract])) OR (Herbalism[Title/Abstract])) OR (Extracts, Plant[Title/Abstract])) OR (Plant Extract[Title/Abstract])) OR (Extract, Plant[Title/Abstract])) OR (Herbal Preparation[Title/Abstract])) OR (Plant Preparation[Title/Abstract])) OR (Preparation, Plant[Title/Abstract])) OR (Traditional Chinese Medicine[Title/Abstract])) OR (Chung I Hsueh[Title/Abstract])) OR (Traditional Medicine, Chinese[Title/Abstract])) OR (Zhong Yi Xue[Title/Abstract])) OR (Chinese Traditional Medicine[Title/Abstract])) OR (Chinese Medicine, Traditional[Title/Abstract])) OR (Traditional Tongue Diagnosis[Title/Abstract])) OR (Tongue Diagnos*[Title/Abstract])) OR (Tongue Assessment, Traditional[Title/Abstract])) OR (Tibetan Traditional Medicine[Title/Abstract])) OR (Tibetan Medicine, Traditional[Title/Abstract])) OR (Medicine, Traditional Tibetan[Title/Abstract])) OR (Traditional Tibetan Medicine[Title/Abstract])) OR (Tibetan Medicine[Title/Abstract])) OR (Medicine, Tibetan[Title/Abstract])) OR (Traditional Medicine, Tibetan[Title/Abstract])))) AND (((((((Multiple sclerosis[Title/Abstract]) OR (Sclerosis, Multiple[Title/Abstract])) OR (Sclerosis, Disseminated[Title/Abstract])) OR (Disseminated Sclerosis[Title/Abstract])) OR (MS (Multiple Sclerosis[Title/Abstract]))) OR (Multiple Sclerosis, Acute Fulminating[Title/Abstract])) OR ("Multiple Sclerosis"[MeSH Terms]))

**Web of science: 46**

(AB=(Essences, Flower OR Bach Rescue Remedy OR Remedy, Bach Rescue OR Rescue Remedy, Bach OR Bach Flower Remedies OR Flower Remedies, Bach OR Remedies, Bach Flower OR Bach Flowers OR Flowers, Bach OR Bach Flower Essences OR Essences, Bach Flower OR Flower Essences, Bach OR Aromatherapies OR Aroma Therapy OR Aroma Therapies OR Therapies, Aroma OR Therapy, Aroma OR Volatile Oils OR Oil, Essential OR Essential Oil OR Oils, Essential OR Essential Oils OR Volatile Oil OR Oil, Volatile OR Medicinal Plant OR Plant, Medicinal OR Medicinal Plants OR Medicinal Herbs OR Herb, Medicinal OR Medicinal Herb OR Herbs, Medicinal OR Pharmaceutical Plants OR Pharmaceutical Plant OR Plant, Pharmaceutical OR Plants, Pharmaceutical OR Healing Plants OR Healing Plant OR Plant, Healing OR Plants, Healing OR Herbal Therapy OR Herb Therapy OR Chinese Drugs, Plant OR Chinese Herbal Drugs OR Herbal Drugs, Chinese OR Plant Extracts, Chinese OR Chinese Plant Extracts OR Extracts, Chinese Plant OR Remedy, Home OR Medicine, Primitive OR Primitive Medicine OR Medicine, Folk OR Folk Medicine OR Medicine, Indigenous OR Indigenous Medicine OR Folk Remedies OR Folk Remedy OR Remedies, Folk OR Remedy, Folk OR Ethnomedicine OR Medicine, Herbal OR Hawaiian Herbal Medicine OR Herbal Medicine, Hawaiian OR Medicine, Hawaiian Herbal OR Herbalism OR Extracts, Plant OR Plant Extract OR Extract, Plant OR Herbal Preparation OR Plant Preparation OR Preparation, Plant OR Traditional Chinese Medicine OR Chung I Hsueh OR Traditional Medicine, Chinese OR Zhong Yi Xue OR Chinese Traditional Medicine OR Chinese Medicine, Traditional OR Traditional Tongue Diagnosis OR Tongue Diagnosis OR Tongue Assessment, Traditional OR Tibetan Traditional Medicine OR Tibetan Medicine, Traditional OR Medicine, Traditional Tibetan OR Traditional Tibetan Medicine OR Tibetan Medicine OR Medicine, Tibetan OR Traditional Medicine, Tibetan) OR TS=(Flower Essences OR Aromatherapy OR Oils, Volatile OR Plants, Medicinal OR Phytotherapy OR Drugs, Chinese Herbal OR Medicine, Traditional OR Herbal Medicine OR Plant Extracts OR Plant Preparations OR Medicine, Chinese Traditional OR Medicine, Tibetan Traditional)) AND (AB=(Multiple sclerosis OR Sclerosis, Multiple OR Sclerosis, Disseminated OR Disseminated Sclerosis OR MS Multiple Sclerosis OR Multiple Sclerosis, Acute Fulminating) OR TS=(Multiple Sclerosis))

**Embase:36**


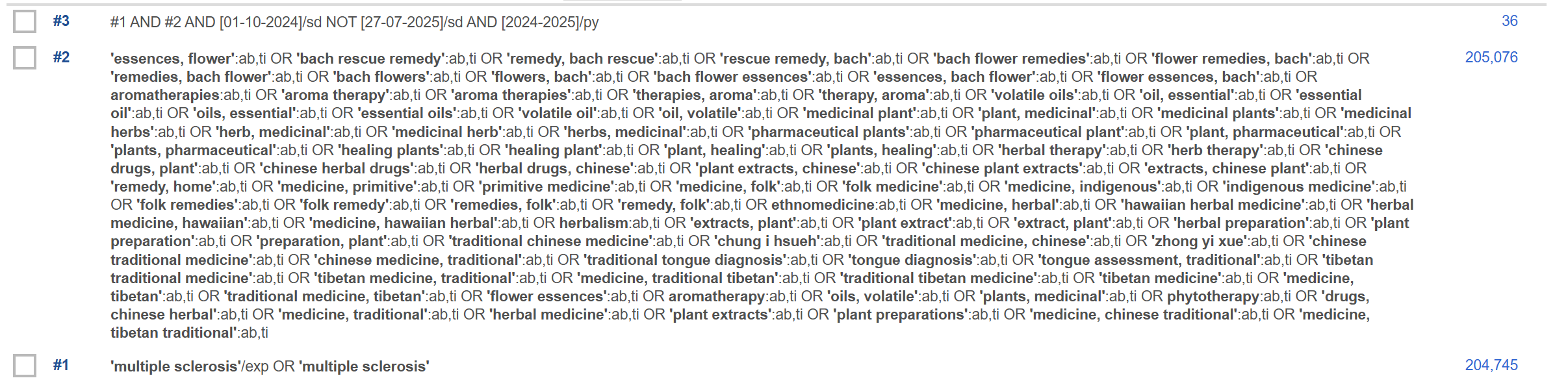


**The Cochrane Central Register of Controlled Trials (CENTRAL) in the Cochrane Library: 2**

ID Search Hits

#1 (multiple sclerosis):ti,ab,kw (Word variations have been searched) 13646

#2 MeSH descriptor: [Multiple Sclerosis] explode all trees 5405

#3 #1 or #2 13646

#4 MeSH descriptor: [Phytotherapy] explode all trees 4981

#5 MeSH descriptor: [Medicine, Traditional] explode all trees 2317

#6 MeSH descriptor: [Plants, Medicinal] explode all trees 1125

#7 MeSH descriptor: [Herbal Medicine] explode all trees 96

#8 MeSH descriptor: [Plant Preparations] explode all trees 15284

#9 MeSH descriptor: [Plant Extracts] explode all trees 11038

#10 MeSH descriptor: [Drugs, Chinese Herbal] explode all trees 4692

#11 #4 or #5 or #6 or #7 or #8 or #9 or #10 17958

#12 MeSH descriptor: [Medicine, Chinese Traditional] explode all trees 1830

#13 MeSH descriptor: [Plants, Medicinal] explode all trees 1125

#14 #11 or #12 or #13 17958

#15 (Sclerosis, Disseminated):ti,ab,kw (Word variations have been searched) 165

#16 (Sclerosis, Multiple):ti,ab,kw (Word variations have been searched) 13646

#17 (Disseminated Sclerosis):ti,ab,kw (Word variations have been searched) 165

#18 #3 or #15 or #16 or #17 13680

#19 #18 and #14 with Publication Year from 2024 to 2025, in Trials 2

**Chinese National Knowledge Infrastructure Database (CNKI): 9**


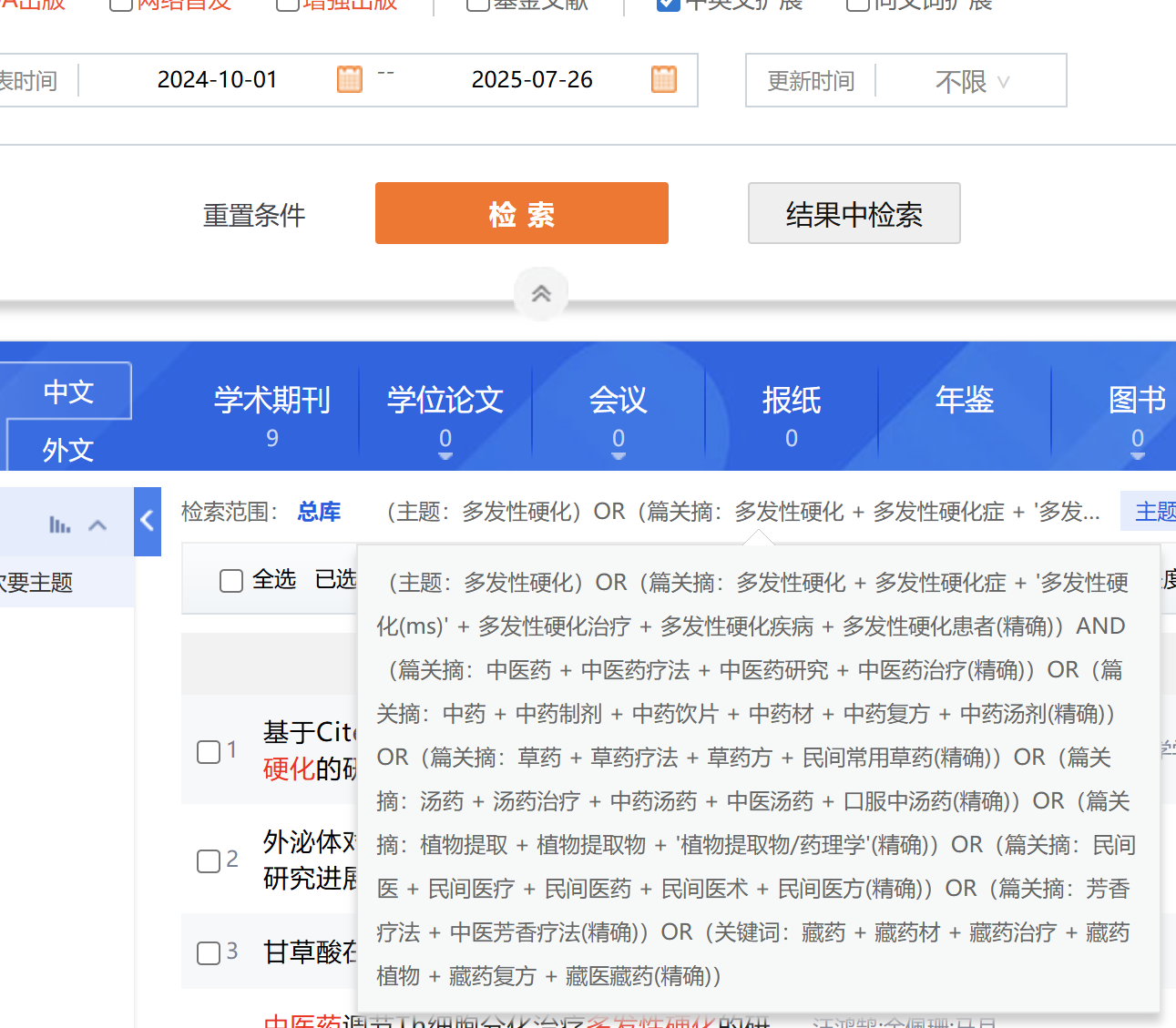


**Wanfang data：56**


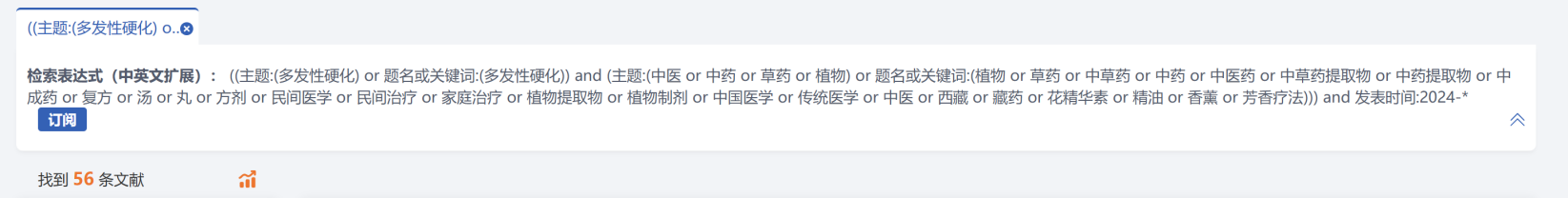


**The VIP information resource integration service platform (cqvip) :16**

M=多发性硬化 AND M=(植物 or 草药 or 中草药 or 中药 or 中医药 or 中草药提取物 or 中药提取物 or 中成药 or 复方 or 汤 or 丸 or 方剂 or 民间医学 or 民间治疗 or 家庭治疗 or 植物提取物 or 植物制剂 or 中国医学 or 传统医学 or 中医 or 西藏 or 藏药 or 花精华素 or 精油 or 香薰 or 芳香疗法)

**SinoMed: 36**


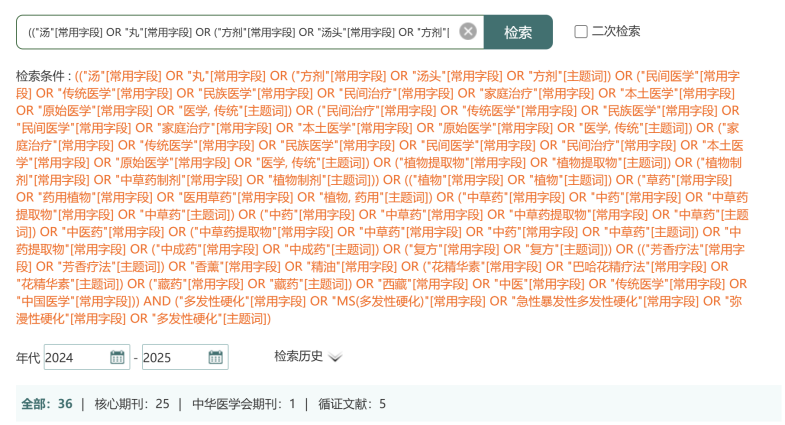


# Appendix 2. The chemical and botanical characterization of the involved preparation according to the “Four Pillars of Best Practice”.

**(A)
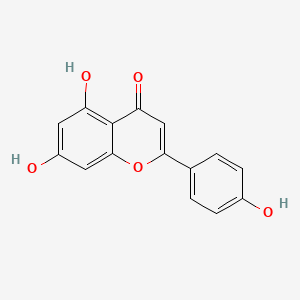
(B)
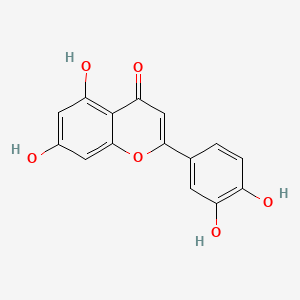
(C)
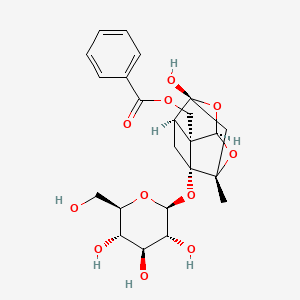
(D)
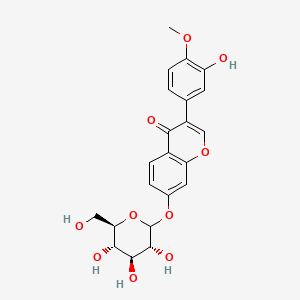
**

**(E)
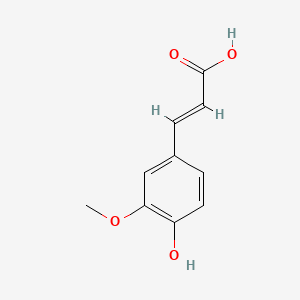
(F)
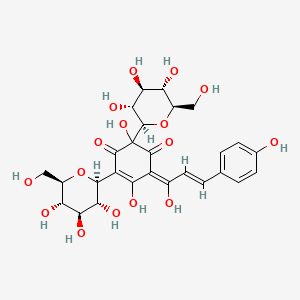
(G)
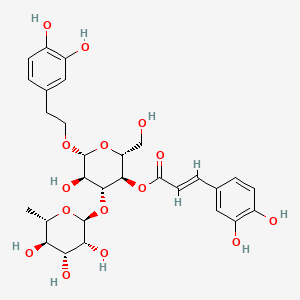
(H)
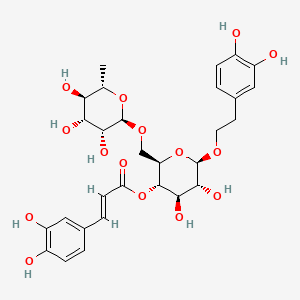
**

**(I)
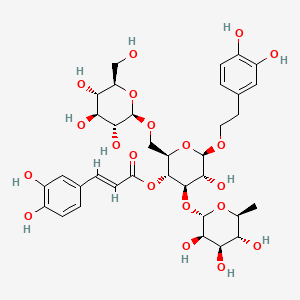
(J)
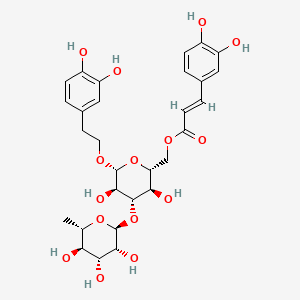
(K)
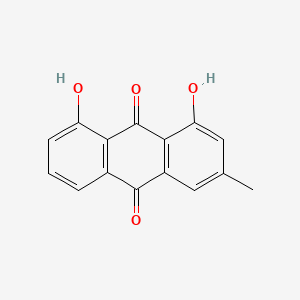
(L)
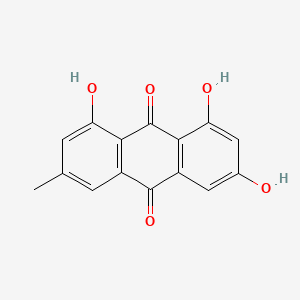
**

**(M)
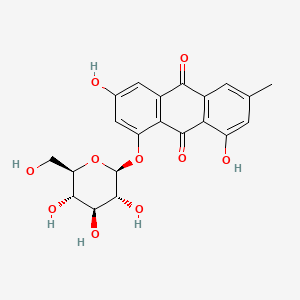
(N)
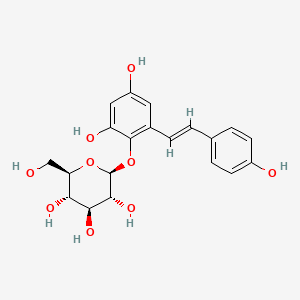
(O)
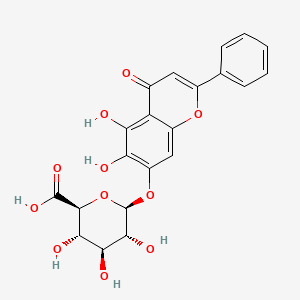
(P)
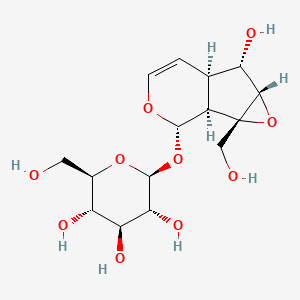
**

**(Q)
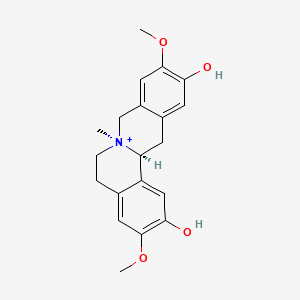
(R)
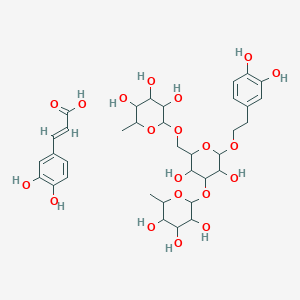
(S)
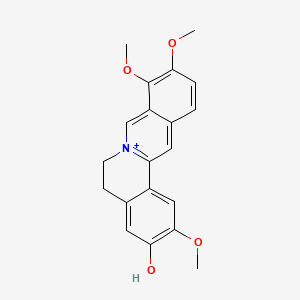
(T)
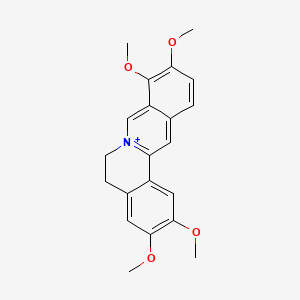
**

**(U)
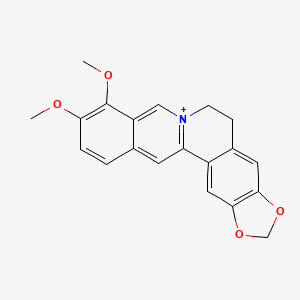
(V)
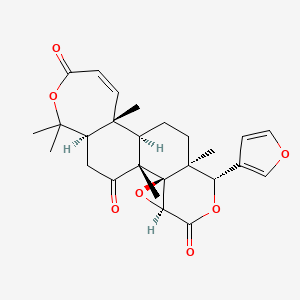
(W)
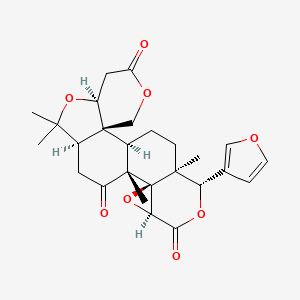
(X)
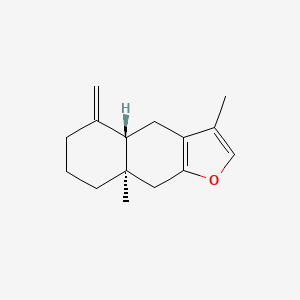
**

**(Y)
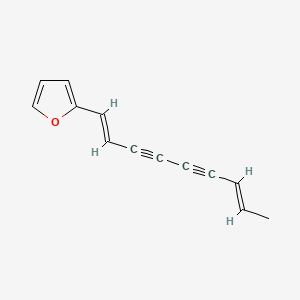
(Z)
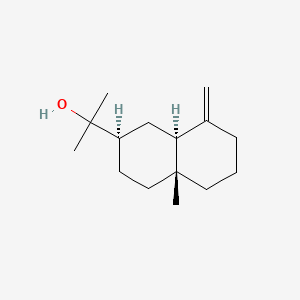
(AA)
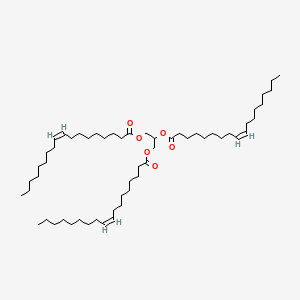
(AB)
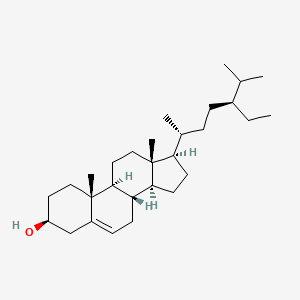
**

**(AC)
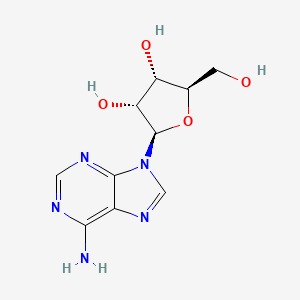
(AD)
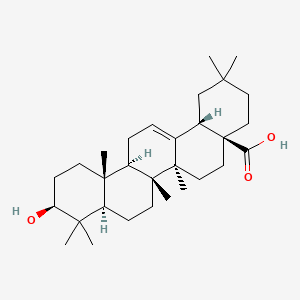
(AE)
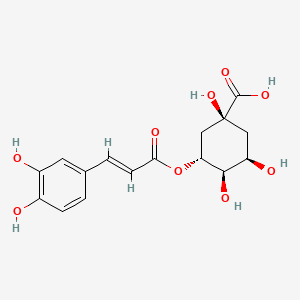
(AF)
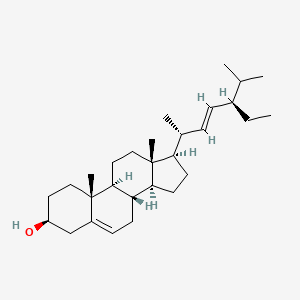
**

**(AG)
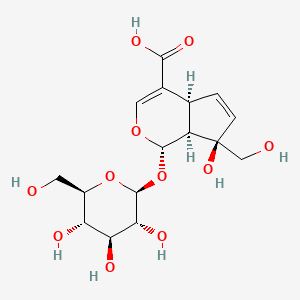
(AH)
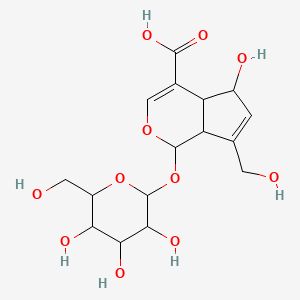
(AI)
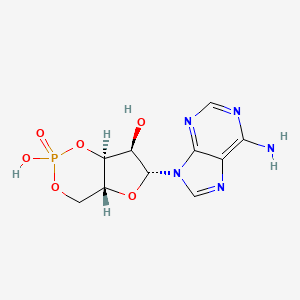
(AJ)
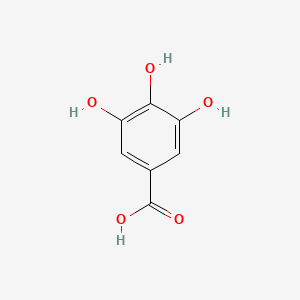
**

**(AK)
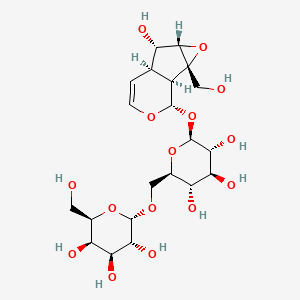
(AL)
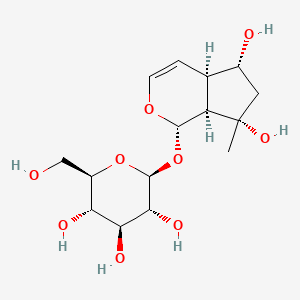
(AM)
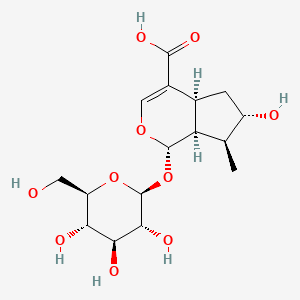
(AN)
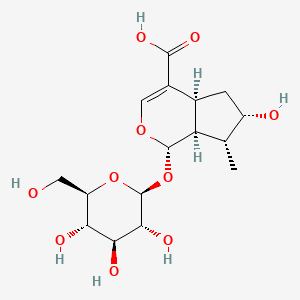
**

**(AO)
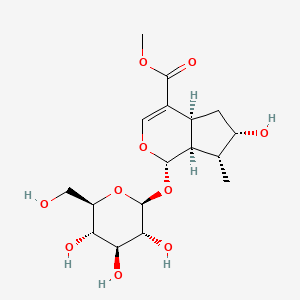
(AP)
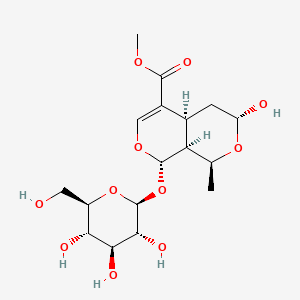
(AQ)
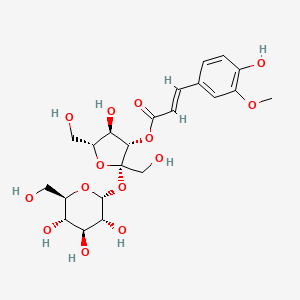
(AR)
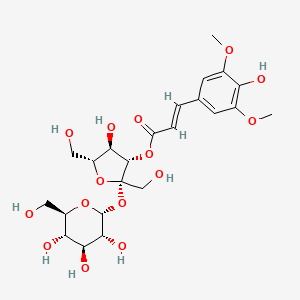
**

**(AS)
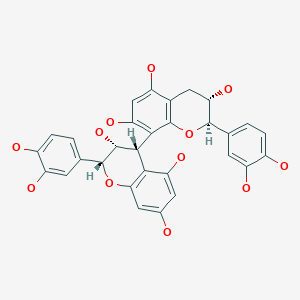
(AT)
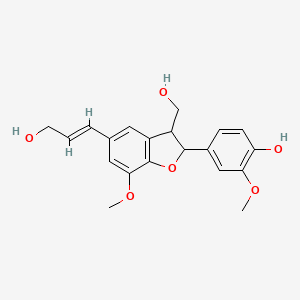
(AU)
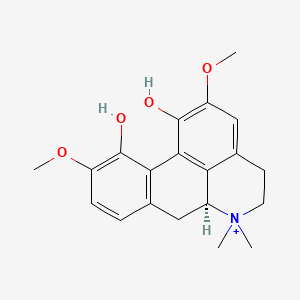
(AV)
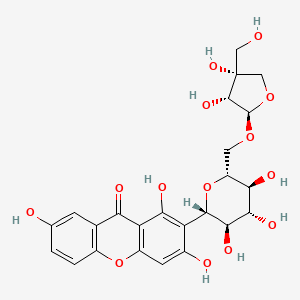
**

**(AW)
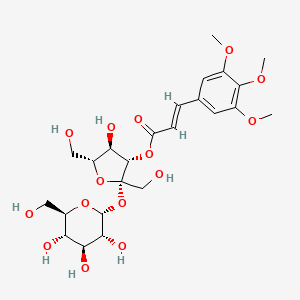
(AX)
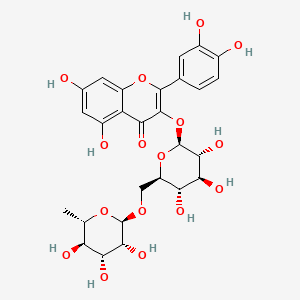
(AY)
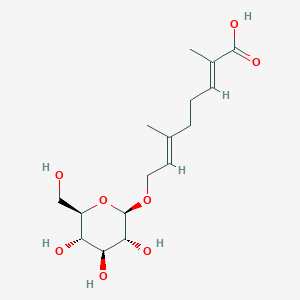
(AZ)
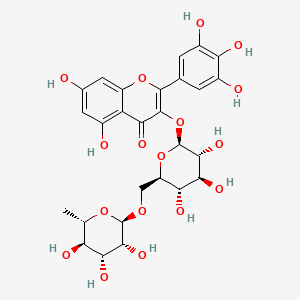
**

**(BA)
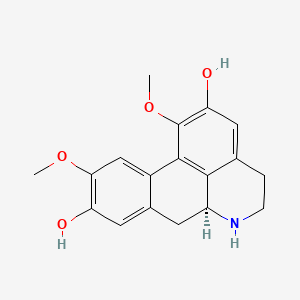
(BB)
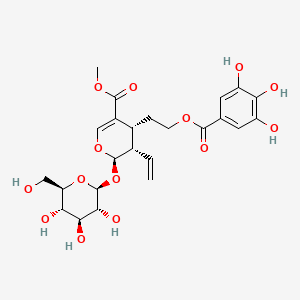
(BC)
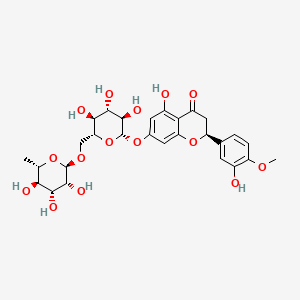
(BD)
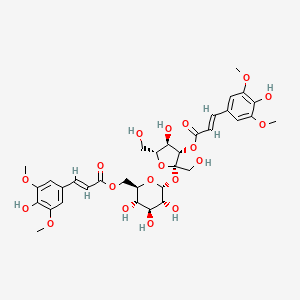
**

**(BE)
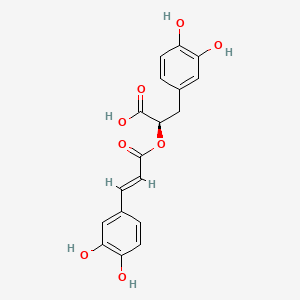
(BF)
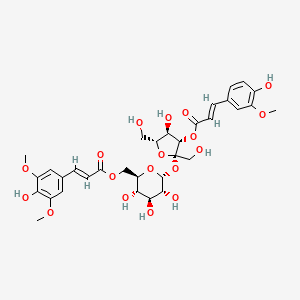
(BG)
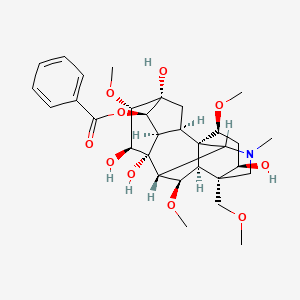
(BH)
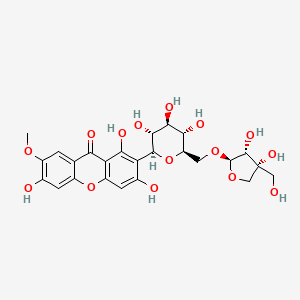
**

**(BI)
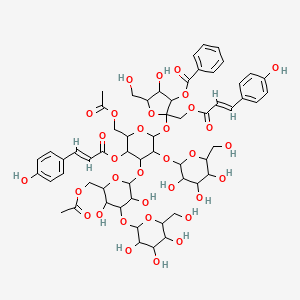
(BJ)
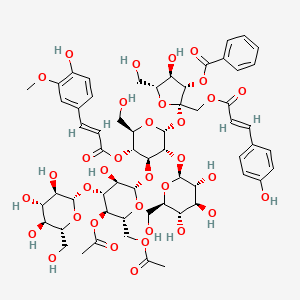
(BK)
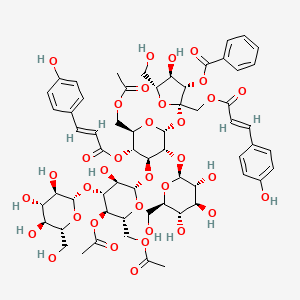
(BL)
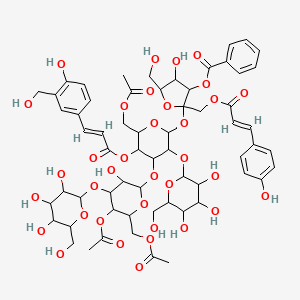
**

**(BM)
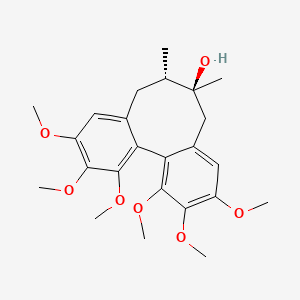
(BP)
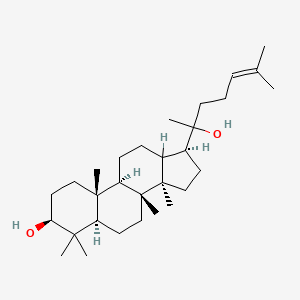
**

Figure 1. The structure of active compounds included in this systematic review. (A) Apigenin (CID:[5280443](https://pubchem.ncbi.nlm.nih.gov/compound/5280443)) in Achillea millefolium L. (B) Luteolin (CID:[5280445](https://pubchem.ncbi.nlm.nih.gov/compound/5280445)) in Achillea millefolium L. (C) Paeoniflorin (CID:[442534](https://pubchem.ncbi.nlm.nih.gov/compound/442534)) in Qishen Huanwu capsule (D) Calycosin-7-glucoside (CID:[71571502](https://pubchem.ncbi.nlm.nih.gov/compound/71571502)) in Qishen Huanwu capsule (E) Ferulicacid (CID:[445858](https://pubchem.ncbi.nlm.nih.gov/compound/445858)) in Qishen Huanwu capsule (F) Hydroxysafflor Yellow A (CID:[6443665](https://pubchem.ncbi.nlm.nih.gov/compound/6443665)) in Qishen Huanwu capsule (G) Acteoside (CID:[5281800](https://pubchem.ncbi.nlm.nih.gov/compound/5281800)) in Bu Shen Yi Sui capsule (Er Huang Fang). (H) Forsythiaside (CID:[5281773](https://pubchem.ncbi.nlm.nih.gov/compound/5281773)) in Bu Shen Yi Sui capsule (Er Huang Fang). (I) Echinacoside (CID:[5281771](https://pubchem.ncbi.nlm.nih.gov/compound/5281771)) in Bu Shen Yi Sui capsule (Er Huang Fang) and Dihuang Yinzi decoction. (J) Isoacteoside (CID:[6476333](https://pubchem.ncbi.nlm.nih.gov/compound/6476333)) in Bu Shen Yi Sui capsule (Er Huang Fang). (K) Chrysophanol (CID:[10208](https://pubchem.ncbi.nlm.nih.gov/compound/10208)) in Bu Shen Yi Sui capsule (Er Huang Fang). (L) Emodin (CID:[3220](https://pubchem.ncbi.nlm.nih.gov/compound/3220)) in Bu Shen Yi Sui capsule (Er Huang Fang). (M) Emodin-8-β-D-glucoside (CID:[99649](https://pubchem.ncbi.nlm.nih.gov/compound/99649)) in Bu Shen Yi Sui capsule (Er Huang Fang). (N) 2,3,5,4'-Tetrahydroxystilbene 2-O-beta-D-glucoside (CID:[5321884](https://pubchem.ncbi.nlm.nih.gov/compound/5321884)) in Bu Shen Yi Sui capsule (Er Huang Fang). (O) Baicalin (CID:[64982](https://pubchem.ncbi.nlm.nih.gov/compound/64982)) in Dihuang Heji capsule. (P) Catalpol (CID:[91520](https://pubchem.ncbi.nlm.nih.gov/compound/91520)) in Toufengling decoction and Ziyin Guben Granule. (Q) Phellodendrine (CID:[3081405](https://pubchem.ncbi.nlm.nih.gov/compound/3081405)) in Si Miao pills. (R) Magnolidin (CID:[5477035](https://pubchem.ncbi.nlm.nih.gov/compound/5477035)) in Si Miao pills. (S) Jatrorrhizine (CID:[72323](https://pubchem.ncbi.nlm.nih.gov/compound/72323)) in Si Miao pills. (T) Palmatine (CID:[19009](https://pubchem.ncbi.nlm.nih.gov/compound/19009)) in Si Miao pills. (U) Berberine (CID:[2353](https://pubchem.ncbi.nlm.nih.gov/compound/2353)) in Si Miao pills. (V) Obacunone (CID:[119041](https://pubchem.ncbi.nlm.nih.gov/compound/119041)) in Si Miao pills. (W) Limonin (CID:[179651](https://pubchem.ncbi.nlm.nih.gov/compound/179651)) in Si Miao pills. (X) Atractylon (CID:[3080635](https://pubchem.ncbi.nlm.nih.gov/compound/3080635)) in Si Miao pills. (Y) Atractylodin (CID:[5321047](https://pubchem.ncbi.nlm.nih.gov/compound/5321047)) in Si Miao pills. (Z) Beta-Eudesmol (CID:[91457](https://pubchem.ncbi.nlm.nih.gov/compound/91457)) in Si Miao pills. (AA) Glycerol trioleate (CID:[5497163](https://pubchem.ncbi.nlm.nih.gov/compound/5497163)) in Si Miao pills. (AB) Gamma-Sitosterol (CID:[457801](https://pubchem.ncbi.nlm.nih.gov/compound/457801)) in Si Miao pills. (AC) Adenosine (CID:[60961](https://pubchem.ncbi.nlm.nih.gov/compound/60961)) in Si Miao pills. (AD) Oleanolic Acid (CID:[10494](https://pubchem.ncbi.nlm.nih.gov/compound/10494)) in Si Miao pills. (AE) Chlorogenic Acid (CID:[1794427](https://pubchem.ncbi.nlm.nih.gov/compound/1794427)) in Si Miao pills. (AF) Stigmasterol (CID:[5280794](https://pubchem.ncbi.nlm.nih.gov/compound/5280794)) in Ziyin Guben Granule. (AG) Monotropein (CID:[73466](https://pubchem.ncbi.nlm.nih.gov/compound/73466)) in Dihuang Yinzi decoction. (AH) Desacetyl asperulosidic acid (CID:[12315348](https://pubchem.ncbi.nlm.nih.gov/compound/12315348)) in Dihuang Yinzi decoction. (AI) [Cyclic Adenosine Monophosphate](https://www.ncbi.nlm.nih.gov/pcsubstance/?term=) (CID:[6076](https://pubchem.ncbi.nlm.nih.gov/compound/6076)) in Dihuang Yinzi decoction. (AJ) Gallic Acid (CID:[370](https://pubchem.ncbi.nlm.nih.gov/compound/370)) in Dihuang Yinzi decoction. (AK) Rehmannioside A (CID:[6325881](https://pubchem.ncbi.nlm.nih.gov/compound/6325881)) in Dihuang Yinzi decoction. (AL) Leonuride (CID:[6325127](https://pubchem.ncbi.nlm.nih.gov/compound/6325127)) in Dihuang Yinzi decoction. (AM) 8-Epiloganic Acid (CID:[158144](https://pubchem.ncbi.nlm.nih.gov/compound/158144)) in Dihuang Yinzi decoction. (AN) Loganic Acid (CID:[89640](https://pubchem.ncbi.nlm.nih.gov/compound/89640)) in Dihuang Yinzi decoction. (AO) Loganin (CID:[87691](https://pubchem.ncbi.nlm.nih.gov/compound/87691)) in Dihuang Yinzi decoction. (AP) Morroniside (CID:[11228693](https://pubchem.ncbi.nlm.nih.gov/compound/11228693)) in Dihuang Yinzi decoction. (AQ) Sibiricose A5 (CID:[6326020](https://pubchem.ncbi.nlm.nih.gov/compound/6326020)) in Dihuang Yinzi decoction. (AR) Sibiricose A6 (CID:[6326021](https://pubchem.ncbi.nlm.nih.gov/compound/6326021)) in Dihuang Yinzi decoction. (AS) Procyanidin B1 (CID:[11250133](https://pubchem.ncbi.nlm.nih.gov/compound/11250133)) in Dihuang Yinzi decoction. (AT) Dehydrodiconiferyl alcohol (CID:[5372367](https://pubchem.ncbi.nlm.nih.gov/compound/5372367)) in Dihuang Yinzi decoction. (AU) Magnoflorine (CID:[73337](https://pubchem.ncbi.nlm.nih.gov/compound/73337)) in Dihuang Yinzi decoction. (AV) Sibiricaxanthone A (CID:[21581292](https://pubchem.ncbi.nlm.nih.gov/compound/21581292)) in Dihuang Yinzi decoction. (AW) Glomeratose A (CID:[11972358](https://pubchem.ncbi.nlm.nih.gov/compound/11972358)) in Dihuang Yinzi decoction. (AX) Rutin (CID:[5280805](https://pubchem.ncbi.nlm.nih.gov/compound/5280805)) in Dihuang Yinzi decoction. (AY) Kankanoside O (CID:[49788111](https://pubchem.ncbi.nlm.nih.gov/compound/49788111)) in Dihuang Yinzi decoction. (AZ) Myricetin-3-O-rutinoside (CID:[21577860](https://pubchem.ncbi.nlm.nih.gov/compound/21577860)) in Dihuang Yinzi decoction. (BA) Laurolitsine (CID:[22179](https://pubchem.ncbi.nlm.nih.gov/compound/22179)) in Dihuang Yinzi decoction. (BB) Cornuside (CID:[131348](https://pubchem.ncbi.nlm.nih.gov/compound/131348)) in Dihuang Yinzi decoction. (BC) Hesperidin (CID:[10621](https://pubchem.ncbi.nlm.nih.gov/compound/10621)) in Dihuang Yinzi decoction. (BD) 3',6-Disinapoylsucrose (CID:[11968389](https://pubchem.ncbi.nlm.nih.gov/compound/11968389)) in Dihuang Yinzi decoction. (BE) Rosmarinic Acid (CID:[5281792](https://pubchem.ncbi.nlm.nih.gov/compound/5281792)) in Dihuang Yinzi decoction. (BF) Arillanin A (CID:[11968790](https://pubchem.ncbi.nlm.nih.gov/compound/11968790)) in Dihuang Yinzi decoction. (BG) Benzoylmesaconine (CID:[24832659](https://pubchem.ncbi.nlm.nih.gov/compound/24832659)) in Dihuang Yinzi decoction. (BH) Polygalaxanthone III (CID:[11169063](https://pubchem.ncbi.nlm.nih.gov/compound/11169063)) in Dihuang Yinzi decoction. (BI) Tenuifoliose J (CID:[145865805](https://pubchem.ncbi.nlm.nih.gov/compound/145865805)) in Dihuang Yinzi decoction. (BJ) Tenuifoliose B (CID:[163191789](https://pubchem.ncbi.nlm.nih.gov/compound/163191789)) in Dihuang Yinzi decoction. (BK) Tenuifoliose H (CID:[10329080](https://pubchem.ncbi.nlm.nih.gov/compound/10329080)) in Dihuang Yinzi decoction. (BL) Tenuifoliose A (CID:[145865806](https://pubchem.ncbi.nlm.nih.gov/compound/145865806)) in Dihuang Yinzi decoction. (BM) Schisandrin (CID:[3001664](https://pubchem.ncbi.nlm.nih.gov/compound/3001664)) in Dihuang Yinzi decoction. (BN) Asarone (CID:[636822](https://pubchem.ncbi.nlm.nih.gov/compound/636822)) in Dihuang Yinzi decoction. (BO) Astragaloside IV (CID:[13943297](https://pubchem.ncbi.nlm.nih.gov/compound/13943297)) in Yisui granules. (BP) Ginsenosides (CID:[3086007](https://pubchem.ncbi.nlm.nih.gov/compound/3086007)) in Guilu Yisui capsules.

**Table 1.** Multiherbal

| **Study** | **Species, source, concentration** | **Quality control reported? (Y/N)** | **Chemical analysis reported? (Y/N)** |
| --- | --- | --- | --- |
| Ayoobi et al., 2019 | - Achillea millefolium L. [Asteraceae] - Drug Specifications: 250/500mg per capsule - Isfahan Botany Herbarium (specimen no. 9757) | Y-Prepared according to aqueous extract (Iran) | Y- HPLC |
| Bi, 2015 | - Astragalus mongholicus Bunge [Fabaceae;Astragali radix] 120g - Pseudostellaria heterophylla (Miq.) Pax [Caryophyllaceae;Pseudostellariae radix] 80g - Prunus persica (L.) Batsch [Rosaceae; Persicae semen] 40g - Carthamus tinctorius L. [Asteraceae;Carthami flos] 48g - Angelica sinensis (Oliv.) Diels [Apiaceae;Angelicae sinensis radix] 40g - Paeonia veitchii Lynch [Paeoniaceae;Paeoniae radix rubra] 48g - Conioselinum anthriscoides 'Chuanxiong' [Apiaceae;Chuanxiong rhizoma] 40g - Scrophularia ningpoensis Hemsl. [Scrophulariaceae;Scrophulariae radix] 48g - Haliotis diversicolor Reeve [Haliotidae; Haliotidis concha] 48g - Uncaria rhynchophylla (Miq.) Miq. ex Havil. [Rubiaceae; Uncariae ramulus cum uncis] 48 g - Ophiopogon japonicus (Thunb.) Ker Gawl. [Asparagaceae; Ophiopogonis radix] 40 g - Achyranthes bidentata Blume [Amaranthaceae; Achyranthis bidentatae radix] 40 g - Rheum palmatum L. [Polygonaceae; Rhei radix et rhizoma] 40 g - Forsythia suspensa (Thunb.) Vahl [Oleaceae; Forsythiae fructus] 48 g - Lumbricus [Oligochaeta; Lumbrici] 40 g - Whitmania pigra Whitman [Hirudinidae; Hirudo] 40 g - Arisaema amurense Maxim. [Araceae;[arisaema cum bile](https://mpns.science.kew.org/mpns-portal/drugDetail?drugName=arisaema+cum+bile&query=Arisaema+cum+Bile&filter=&fuzzy=false&nameType=all)] 60g - Pinellia ternata (Thunb.) Makino [Araceae; Pinelliae rhizoma praeparatum] 32g - Buthus martensii Karsch [Scorpionidae; Scorpio] 24 g - Specification: 0.4g per capsule, equivalent to 3g of crude herb - Chinese Materia Medica Department, Cangzhou Hospital of Integrated TCM-WM | Y-Prepared according to Chinese pharmacopeia | Y- HPLC-ELSD |
| Chen and Fan, 2016 | - Rehmannia glutinosa (Gaertn.) DC. [Orobanchaceae; Rehmanniae radix praeparata] 15 g - Rehmannia glutinosa (Gaertn.) DC. [Orobanchaceae; Rehmanniae radix] 15 g - Rheum palmatum L. or Rheum officinale Baill. [Polygonaceae; Rhei radix et rhizoma] 3 g - Polygonum multiflorum Thunb. [Polygonaceae; Polygoni multiflori radix] 12 g - Fritillaria thunbergii Miq. [Liliaceae; Fritillariae thunbergii bulbus] 9 g - Whitmania pigra Whitman [Hirudinidae; Hirudo] 3 g - Buthus martensii Karsch [Scorpionidae; Scorpio] 3 g - Gastrodia elata Blume [Orchidaceae; Gastrodiae rhizoma] 5 g - Forsythia suspensa (Thunb.) Vahl [Oleaceae; Forsythiae fructus] 9 g - Leonurus japonicus Houtt. [Lamiaceae; Leonuri herba] 15 g - Hospital preparation of Beijing Tiantan Hospital, Capital Medical University | Y-Prepared according to Chinese pharmacopeia | Y-HPLC |
| Dong, 2014 | - Astragalus mongholicus Bunge [Fabaceae; Astragali radix] 30 g - Epimedium brevicornum Maxim. [Berberidaceae; Epimedii herba] 30 g - Morinda officinalis How [Rubiaceae; Morindae officinalis radix] 30 g - Acorus tatarinowii Schott [Acoraceae; Acori tatarinowii rhizoma] 30 g - Paris polyphylla Smith [Melanthiaceae; Paridis rhizoma] 30 g - Rehmannia glutinosa (Gaertn.) DC. [Orobanchaceae; Rehmanniae radix praeparata] 25 g - Ligustrum lucidum W.T.Aiton [Oleaceae; Ligustri lucidi fructus] 25 g - Cuscuta chinensis Lam. [Convolvulaceae; Cuscutae semen] 20 g - Codonopsis pilosula (Franch.) Nannf. [Campanulaceae; Codonopsis radix] 20 g - Bombyx batryticatus [Bombycidae; Bombyx batryticatus] 20 g - Isatis indigotica Fort. [Brassicaceae; Isatidis folium] 20 g - Serissa japonica (Thunb.) Thunb. ex Tanaka [Rubiaceae; Serissae herba] 20 g - Whitmania pigra Whitman [Hirudinidae; Hirudo] 8 g - Funing County Hospital, Hebei Qinhuangdao | Y-Prepared according to Chinese pharmacopeia | Y-HPLC |
| Duan, 2013 | - Achyranthes bidentata Blume [Amaranthaceae; Achyranthis bidentatae radix] 12 g - Astragalus mongholicus Bunge [Fabaceae; Astragali radix] 25 g - Pinellia ternata (Thunb.) Makino [Araceae; Pinelliae rhizoma praeparatum] 12 g - Citrus reticulata Blanco [Rutaceae; Citri reticulatae pericarpium] 20 g - Arisaema cum bile [Araceae; Arisaematis cum bile tuber] 15 g - Bombyx batryticatus [Bombycidae; Bombyx batryticatus] 5 g - Angelica sinensis (Oliv.) Diels [Apiaceae; Angelicae sinensis radix] 10 g - Paeonia veitchii Lynch [Paeoniaceae;Paeoniae radix rubra] 10 g - Conioselinum anthriscoides 'Chuanxiong' [Apiaceae;Chuanxiong rhizoma] 5 g - Coix lacryma-jobi L. var. ma-yuen (Roman.) Stapf [Poaceae; Coicis semen] 15 g - Glycyrrhiza uralensis Fisch. [Fabaceae; Glycyrrhizae radix et rhizoma] 10 g - Lumbricus [Oligochaeta; Lumbrici] 10 g - Poria cocos (Schw.) Wolf [Polyporaceae; Poria] 15 g - Bambusa tuldoides Munro [Poaceae; Bambusae caulis in taeniam] 15 g - Prunus persica (L.) Batsch [Rosaceae; Persicae semen] 10 g | Y-Prepared according to Chinese pharmacopeia | Y-HPLC |
| Fan et al., 2006 | - Rehmannia glutinosa (Gaertn.) DC. [Orobanchaceae; Rehmanniae radix praeparata] 15 g - Rehmannia glutinosa (Gaertn.) DC. [Orobanchaceae; Rehmanniae radix] 15 g - Rheum palmatum L. or Rheum officinale Baill. [Polygonaceae; Rhei radix et rhizoma] 3 g - Polygonum multiflorum Thunb. [Polygonaceae; Polygoni multiflori radix] 12 g - Fritillaria thunbergii Miq. [Liliaceae; Fritillariae thunbergii bulbus] 9 g - Whitmania pigra Whitman [Hirudinidae; Hirudo] 3 g - Buthus martensii Karsch [Scorpionidae; Scorpio] 3 g - Gastrodia elata Blume [Orchidaceae; Gastrodiae rhizoma] 5 g - Forsythia suspensa (Thunb.) Vahl [Oleaceae; Forsythiae fructus] 9 g - Leonurus japonicus Houtt. [Lamiaceae; Leonuri herba] 15 g | Y-Prepared according to Chinese pharmacopeia | Y-UPLC-LTQ-Orbitrap-MS^n^ and UPLC-QTOF-MS/MS |
| Fan et al., 2018 | - Rehmannia glutinosa (Gaertn.) DC. [Orobanchaceae; Rehmanniae radix praeparata] 15 g - Rehmannia glutinosa (Gaertn.) DC. [Orobanchaceae; Rehmanniae radix] 15 g - Rheum palmatum L. or Rheum officinale Baill. [Polygonaceae; Rhei radix et rhizoma] 3 g - Polygonum multiflorum Thunb. [Polygonaceae; Polygoni multiflori radix] 12 g - Fritillaria thunbergii Miq. [Liliaceae; Fritillariae thunbergii bulbus] 9 g - Whitmania pigra Whitman [Hirudinidae; Hirudo] 3 g - Buthus martensii Karsch [Scorpionidae; Scorpio] 3 g - Gastrodia elata Blume [Orchidaceae; Gastrodiae rhizoma] 5 g - Forsythia suspensa (Thunb.) Vahl [Oleaceae; Forsythiae fructus] 9 g - Leonurus japonicus Houtt. [Lamiaceae; Leonuri herba] 15 g - Specification: 0.5g/capsule | Y-Prepared according to Chinese pharmacopeia | Y-UPLC-LTQ-Orbitrap-MS^n^ and UPLC-QTOF-MS/MS |
| Gao et al., 2008 | - Rehmannia glutinosa (Gaertn.) DC. [Orobanchaceae; Rehmanniae radix praeparata] - Cornus officinalis Siebold & Zucc. [Cornaceae; Corni fructus] - Ophiopogon japonicus (Thunb.) Ker Gawl. [Asparagaceae; Ophiopogonis radix] - Cistanche deserticola Y.C.Ma [Orobanchaceae; Cistanches herba] - Paeonia lactiflora Pall. [Paeoniaceae; Paeoniae radix alba] - Acorus tatarinowii Schott [Acoraceae; Acori tatarinowii rhizoma] - Arisaema cum bile [Araceae; Arisaematis cum bile tuber] - Lumbricus [Oligochaeta; Lumbrici] - Curcuma aromatica Salisb. or Curcuma wenyujin Y.H.Chen & C.Ling [Zingiberaceae; Curcumae radix] - Bombyx batryticatus [Bombycidae; Bombyx batryticatus] | Y-Prepared according to Chinese pharmacopeia | Y-HPLC |
| Li and Yan, 2017 | - Glycyrrhiza uralensis Fisch. [Fabaceae; Glycyrrhizae radix et rhizoma] 6 g - Codonopsis pilosula (Franch.) Nannf. [Campanulaceae; Codonopsis radix] 30 g - Atractylodes macrocephala Koidz. [Asteraceae; Atractylodis macrocephalae rhizoma] 15 g - Cistanche deserticola Y.C.Ma [Orobanchaceae; Cistanches herba] 20 g - Epimedium brevicornum Maxim. [Berberidaceae; Epimedii herba] 20 g - Rehmannia glutinosa (Gaertn.) DC. [Orobanchaceae; Rehmanniae radix] 15 g - Salvia miltiorrhiza Bunge [Lamiaceae; Salviae miltiorrhizae radix et rhizoma] 15 g - Poria cocos (Schw.) Wolf [Polyporaceae; Poria] 15 g - Glycyrrhiza uralensis Fisch. [Fabaceae; Glycyrrhizae radix et rhizoma] 6 g - Alpinia oxyphylla Miq. [Zingiberaceae; Alpiniae oxyphyllae fructus] 20 g - Curcuma aromatica Salisb. or Curcuma wenyujin Y.H.Chen & C.Ling [Zingiberaceae; Curcumae radix] 15 g - Ligusticum chuanxiong Hort. [Apiaceae; Chuanxiong rhizoma] 15 g | Y-Prepared according to Chinese pharmacopeia | UV-Vis |
| Li et al., 2019 | - Astragalus mongholicus Bunge [Fabaceae; Astragali radix] 30 g - Epimedium brevicornum Maxim. [Berberidaceae; Epimedii herba] 30 g - Coix lacryma-jobi L. var. ma-yuen (Roman.) Stapf [Poaceae; Coicis semen] 25 g - Alisma plantago-aquatica subsp. orientale (Sam.) Sam. [Alismataceae; Alismatis rhizoma] 25 g - Cuscuta chinensis Lam. [Convolvulaceae; Cuscutae semen] 20 g - Codonopsis pilosula (Franch.) Nannf. [Campanulaceae; Codonopsis radix] 20 g - Rehmannia glutinosa (Gaertn.) DC. [Orobanchaceae; Rehmanniae radix praeparata] 20 g - Paeonia lactiflora Pall. [Paeoniaceae; Paeoniae radix rubra] 20 g - Curcuma zedoaria (Christm.) Roscoe [Zingiberaceae; Curcumae rhizoma] 20 g - Hedyotis diffusa Willd. [Rubiaceae; Hedyotis diffusa herba] 15 g - Ligusticum chuanxiong Hort. [Apiaceae; Chuanxiong rhizoma] 15 g - Paris polyphylla Smith [Melanthiaceae; Paridis rhizoma] 15 g - Sparganium stoloniferum Buch.-Ham. [Typhaceae; Sparganii rhizoma] 10 g - Bombyx batryticatus [Bombycidae; Bombyx batryticatus] 10 g - Buthus martensii Karsch [Scorpionidae; Scorpio] 10 g - Glycyrrhiza uralensis Fisch. [Fabaceae; Glycyrrhizae radix et rhizoma] 6 g | Y-Prepared according to Chinese pharmacopeia | Y-HPLC |
| Li et al., 2024 | - Phellodendron amurense Rupr.[Rutaceae; Phellodendri cortex] - Atractylodes lancea (Thunb.) DC.[Asteraceae; Atractylodis rhizoma] - Coix lacryma-jobi L. var. ma-yuen (Roman.) Stapf [Poaceae; Coicis semen] - Achyranthes bidentata Blume [Amaranthaceae; Achyranthis bidentatae radix] | Y-Prepared according to Chinese pharmacopeia | GC-MS and HS-SPME |
| Lv et al., 2015 | - Rehmannia glutinosa (Gaertn.) DC. [Orobanchaceae; Rehmanniae radix praeparata] 25 g - Rehmannia glutinosa (Gaertn.) DC. [Orobanchaceae; Rehmanniae radix] 25 g - Poria cocos (Schw.) Wolf [Polyporaceae; Poria] 20 g - Paeonia suffruticosa Andrews [Paeoniaceae; Moutan cortex] 10 g - Aconitum carmichaelii Debeaux [Ranunculaceae; Aconiti lateralis radix praeparata] 5 g - Epimedium brevicornum Maxim. [Berberidaceae; Epimedii herba] 30 g - Cistanche deserticola Y.C.Ma [Orobanchaceae; Cistanches herba] 15 g - Achyranthes bidentata Blume [Amaranthaceae; Achyranthis bidentatae radix] 20 g - Astragalus mongholicus Bunge [Fabaceae; Astragali radix] 30 g - Pueraria montana var. lobata (Willd.) Maesen & S.M.Almeida [Fabaceae; Puerariae lobatae radix] 15 g - Ligusticum chuanxiong Hort. [Apiaceae; Chuanxiong rhizoma] 15 g - Cuscuta chinensis Lam. [Convolvulaceae; Cuscutae semen] 20 g - Lumbricus [Oligochaeta; Lumbrici] 8 g - Bombyx batryticatus [Bombycidae; Bombyx batryticatus] 20 g | Y-Prepared according to Chinese pharmacopeia | Y-HPLC |
| Qian and Wang, 2020 | - Rehmannia glutinosa (Gaertn.) DC. [Orobanchaceae; Rehmanniae radix praeparata] - Rehmannia glutinosa (Gaertn.) DC. [Orobanchaceae; Rehmanniae radix] - Angelica sinensis (Oliv.) Diels [Apiaceae; Angelicae sinensis radix] - Chinemys reevesii (Gray) [Geoemydidae; Testudinis plastra] - Paeonia lactiflora Pall. [Paeoniaceae; Paeoniae radix alba] - Adenophora stricta Miq. or Adenophora triphylla (Thunb.) A.DC. [Campanulaceae; Adenophorae radix] - Ophiopogon japonicus (Thunb.) Ker Gawl. [Asparagaceae; Ophiopogonis radix] - Lycium barbarum L. [Solanaceae; Lycii fructus] - Citrus reticulata Blanco [Rutaceae; Citri reticulatae pericarpium] - Zingiber officinale Roscoe [Zingiberaceae; Zingiberis rhizoma] - Phellodendron amurense Rupr. or Phellodendron chinense C.K.Schneid. [Rutaceae; Phellodendri cortex] - Anemarrhena asphodeloides Bunge [Asparagaceae; Anemarrhenae rhizoma] - Cynomorium songaricum Rupr. [Cynomoriaceae; Cynomorii herba] - Achyranthes bidentata Blume [Amaranthaceae; Achyranthis bidentatae radix] - Melia toosendan Siebold & Zucc. [Meliaceae; Toosendan fructus] - Department of Pharmacy, The First Affiliated Hospital of Henan University of Chinese Medicine (Zhengzhou, China) | Y-Prepared according to Chinese pharmacopeia | Y-HPLC |
| Wang et al., 2012 | - Rehmannia glutinosa (Gaertn.) DC. [Orobanchaceae; Rehmanniae radix praeparata] 20 g - Morinda officinalis How [Rubiaceae; Morindae officinalis radix] 10 g - Cornus officinalis Sieb. et Zucc. [Cornaceae; Corni fructus] 12 g - Dendrobium nobile Lindl. [Orchidaceae; Dendrobii caulis] 9 g - Cistanche deserticola Y.C.Ma [Orobanchaceae; Cistanches herba] 10 g - Aconitum carmichaelii Debeaux [Ranunculaceae; Aconiti lateralis radix praeparata] 6 g - Schisandra chinensis (Turcz.) Baill. [Schisandraceae; Schisandrae chinensis fructus] 9 g - Cinnamomum cassia (L.) J.Presl [Lauraceae; Cinnamomi cortex] 3 g - Poria cocos (Schw.) Wolf [Polyporaceae; Poria] 12 g - Ophiopogon japonicus (Thunb.) Ker Gawl. [Asparagaceae; Ophiopogonis radix] 9 g - Acorus tatarinowii Schott [Acoraceae; Acori tatarinowii rhizoma] 30 g - Polygala tenuifolia Willd. [Polygalaceae; Polygalae radix] 10 g - Pharmacy Department, China-Japan Friendship Hospital | Y-Prepared according to Chinese pharmacopeia | Y-UHPLC-Q-TOF-MS/MS |
| Wu et al., 2020 | - Eucommia ulmoides Oliv. [Eucommiaceae; Eucommiae cortex] 15 g - Dioscorea opposita Thunb. [Dioscoreaceae; Dioscoreae rhizoma] 15 g - Poria cocos (Schw.) Wolf [Polyporaceae; Poria] 15 g - Cornus officinalis Sieb. et Zucc. [Cornaceae; Corni fructus] 15 g - Phellodendron chinense Schneid. [Rutaceae; Phellodendri chinensis cortex] 8 g - Cervus elaphus Linnaeus [Cervidae; Cervi cornu colla] 15 g - Epimedium brevicornum Maxim. [Berberidaceae; Epimedii herba] 12 g - Boswellia sacra Flueck. [Burseraceae; Olibanum] 3 g - Commiphora myrrha (Nees) Engl. [Burseraceae; Myrrha] 3 g - Strychnos nux-vomica L. [Loganiaceae; Strychni semen praeparata] 0.1 g - Department of Chinese Materia Medica, The First Affiliated Hospital of Guangxi University of Chinese Medicine | Y-Prepared according to Chinese pharmacopeia | Y-HPLC |
| Yuan et al., 2025 | - Ephedra sinica Stapf [Ephedraceae; Ephedrae Herba] 6g - Cinnamomum cassia (L.) J. Presl [Lauraceae; Cinnamomi cassiae cortex] 10g - Angelica sinensis (Oliv.) Diels [Apiaceae; Angelicae sinensis radix] 10g - Panax ginseng C.A. Meyer [Araliaceae; Ginseng radix] 6g - Gypsum Fibrosum [—; Gypsum Fibrosum] 20g - Zingiber officinale Roscoe [Zingiberaceae; Zingiberis rhizoma] 9g - Glycyrrhiza uralensis Fisch. ex DC. [Fabaceae; Liquiritiae radix] 9g - Ligusticum chuanxiong Hort. [Apiaceae; Chuanxiong rhizoma] 9g - Prunus armeniaca L. (seed) [Rosaceae; Armeniacae semen] 10g - Phyllostachys nigra (Lodd. ex Lindl.) Munro [Poaceae; Bambusae caulis in taeniam] 20mL | Y-Prepared according to Chinese pharmacopeia | GC-MS |
| Zhao et al., 2023 | - Rehmannia glutinosa (Gaertn.) DC. [Orobanchaceae; Rehmanniae radix praeparata] - Dioscorea hypoglauca Palib. [Dioscoreaceae; Dioscoreae hypoglaucae rhizoma] - Aconitum carmichaelii Debeaux [Ranunculaceae; Aconiti lateralis radix praeparata] - Notopterygium incisum Ting ex H.T.Chang [Apiaceae; Notopterygii rhizoma et radix] - Angelica sinensis (Oliv.) Diels [Apiaceae; Angelicae sinensis radix] - Anemarrhena asphodeloides Bunge [Asparagaceae; Anemarrhenae rhizoma] - Curcuma aromatica Salisb. [Zingiberaceae; Curcumae radix] - Cervus elaphus Linnaeus [Cervidae; Cervi cornu colla] - Acorus tatarinowii Schott [Acoraceae; Acori tatarinowii rhizoma] - Gardenia jasminoides Ellis [Rubiaceae; Gardeniae fructus] - Siegesbeckia orientalis L. [Asteraceae; Siegesbeckiae herba] - Glycyrrhiza uralensis Fisch. [Fabaceae; Glycyrrhizae radix et rhizoma] - Baoding Second Central Hospital, Hebei Province, China | Y-Prepared according to Chinese pharmacopeia | Y-HPLC |

**Table 2.** Commercial Chinese polyherbal preparations

| **Study** | **Formulation** | **Source** | **Species, concentration** | **Quality control reported? (Y/N)** | **Chemical anaysis reported? (Y/N)** |
| --- | --- | --- | --- | --- | --- |
| Chen et al., 2006 | *Guilu Yisui* capsule | Shijiazhuang Yiling Pharmaceutical Co., Ltd | - Panax ginseng C.A.Mey. [Araliaceae; Ginseng radix et rhizoma] - ·Cervus nippon Temminck or Cervus elaphus Linnaeus [Cervidae; Cervi cornu pantotrichum] - Chinemys reevesii (Gray) [Geoemydidae; Testudinis plastra] - Cuscuta chinensis Lam. [Convolvulaceae; Cuscutae semen] - Polygonum multiflorum Thunb. [Polygonaceae; Polygoni multiflori radix] - Lycium barbarum L. [Solanaceae; Lycii fructus] - Buthus martensii Karsch [Scorpionidae; Scorpio] - Spatholobus suberectus Dunn [Fabaceae; Spatholobi caulis] | Y-Prepared according to Chinese pharmacopeia | Y-UPLC |
| Jie, 2024 | *Yisui* granules | Xi'an Renren Pharmaceutical Co., Ltd. | - Rehmannia glutinosa (Gaertn.) DC. [Orobanchaceae; Rehmanniae radix praeparata] - Lycium barbarum L. [Solanaceae; Lycii fructus] - Salvia miltiorrhiza Bunge [Lamiaceae; Salviae miltiorrhizae radix et rhizoma] - Morinda officinalis How [Rubiaceae; Morindae officinalis radix] - Cornus officinalis Siebold & Zucc. [Cornaceae; Corni fructus] - Paeonia suffruticosa Andrews [Paeoniaceae; Moutan cortex] - Astragalus mongholicus Bunge [Fabaceae; Astragali radix] - Cercis chinensis Bunge [Fabaceae; Cercidis chinensis flos] - Strychnos nux-vomica L. [Loganiaceae; Strychni semen pulvis] - Cordyceps sinensis (Berk.) Sacc. [Clavicipitaceae; Cordyceps] - Angelica sinensis (Oliv.) Diels [Apiaceae; Angelicae sinensis radix] - Ligusticum chuanxiong Hort. [Apiaceae; Chuanxiong rhizoma] - Cervus nippon Temminck or Cervus elaphus Linnaeus [Cervidae; Cervi cornu pantotrichum] - Polygonatum sibiricum Redouté [Asparagaceae; Polygonati rhizoma] - Dioscorea oppositifolia L. or Dioscorea opposita Thunb. [Dioscoreaceae; Dioscoreae rhizoma] - Spatholobus suberectus Dunn [Fabaceae; Spatholobi caulis] - Panax ginseng C.A.Mey. [Araliaceae; Ginseng radix et rhizoma] - Specification: 7.5g per bag | Y-Prepared according to Chinese pharmacopeia | Y-HPLC-ELSD |
| Li, 2017 | *Tripterygium Wilfordii Polyglycosides* | Hunan Qianjin Xieli Pharmaceutical Co., Ltd. | Tripterygium wilfordii Hook.f. [Celastraceae; Tripterygii wilfordii radix] | Y-Prepared according to Chinese pharmacopeia | Y-UPLC-MS |
| Zhang, 2021 | *Yisui* granules | Changchun Xinyu Pharmaceutical Co., Ltd. | - Rehmannia glutinosa (Gaertn.) DC. [Orobanchaceae; Rehmanniae radix praeparata] - Lycium barbarum L. [Solanaceae; Lycii fructus] - Salvia miltiorrhiza Bunge [Lamiaceae; Salviae miltiorrhizae radix et rhizoma] - Morinda officinalis How [Rubiaceae; Morindae officinalis radix] - Cornus officinalis Siebold & Zucc. [Cornaceae; Corni fructus] - Paeonia suffruticosa Andrews [Paeoniaceae; Moutan cortex] - Astragalus mongholicus Bunge [Fabaceae; Astragali radix] - Cercis chinensis Bunge [Fabaceae; Cercidis chinensis flos] - Strychnos nux-vomica L. [Loganiaceae; Strychni semen pulvis] - Cordyceps sinensis (Berk.) Sacc. [Clavicipitaceae; Cordyceps] - Angelica sinensis (Oliv.) Diels [Apiaceae; Angelicae sinensis radix] - Ligusticum chuanxiong Hort. [Apiaceae; Chuanxiong rhizoma] - Cervus nippon Temminck or Cervus elaphus Linnaeus [Cervidae; Cervi cornu pantotrichum] - Polygonatum sibiricum Redouté [Asparagaceae; Polygonati rhizoma] - Dioscorea oppositifolia L. or Dioscorea opposita Thunb. [Dioscoreaceae; Dioscoreae rhizoma] - Spatholobus suberectus Dunn [Fabaceae; Spatholobi caulis] - Panax ginseng C.A.Mey. [Araliaceae; Ginseng radix et rhizoma] - Specification: 15g per bag | Y-Prepared according to Chinese pharmacopeia | Y-HPLC-ELSD |
| Li et al., 2025 | *Simiao Pills* | Jilin Zixin Pharmaceutical Industrial Co., Ltd. | - Phellodendron amurense Rupr.[Rutaceae; Phellodendri cortex] - Atractylodes lancea (Thunb.) DC.[Asteraceae; Atractylodis rhizoma] - Coix lacryma-jobi L. var. ma-yuen (Roman.) Stapf [Poaceae; Coicis semen] - Achyranthes bidentata Blume [Amaranthaceae; Achyranthis bidentatae radix] - Specification: 6g per bag | Y-Prepared according to Chinese pharmacopeia | GC-MS and HS-SPME |
